# Molecular signature of eutopic endometrium in endometriosis based on the multi-omics integrative synthesis

**DOI:** 10.1007/s10815-020-01833-3

**Published:** 2020-05-30

**Authors:** Erika Prašnikar, Jure Knez, Borut Kovačič, Tanja Kunej

**Affiliations:** 1 Department of Reproductive Medicine and Gynecological Endocrinology, University Medical Centre Maribor, 2000 Maribor, Slovenia; 2 Department of Gynecological and Breast Oncology, University Medical Centre Maribor, 2000 Maribor, Slovenia; 3 Biotechnical Faculty, Department of Animal Science, University of Ljubljana, 1000 Ljubljana, Slovenia

**Keywords:** Endometriosis, Expression signature, Gene set enrichment analysis (GSEA), Genome-wide study, Multi-omics data integration

## Abstract

**Purpose:**

To synthesise data from genome-wide studies reporting molecular signature of eutopic endometrium through the phases of the menstrual cycle in endometriosis.

**Methods:**

Extraction of data from publications reporting genetic signatures characterising endometrium associated with endometriosis. The nomenclature of extracted differentially expressed transcripts and proteins was adopted according to the HUGO Gene Nomenclature Committee (HGNC). Loci were further sorted according to the different phases of the menstrual cycle, i.e. menstrual (M), proliferative (P), secretory (S), early-secretory (ES), mid-secretory (MS), late-secretory (LS), and not specified (N/S) if the endometrial dating was not available. Enrichment analysis was performed using the DAVID bioinformatics tool.

**Results:**

Altered molecular changes were reported by 21 studies, including 13 performed at the transcriptomic, 6 at proteomic, and 2 at epigenomic level. Extracted data resulted in a catalogue of total 670 genetic causes with available 591 official gene symbols, i.e. M = 3, P = 188, S = 81, ES = 82, MS = 173, LS = 36, and N/S = 28. Enriched pathways included *oestrogen signalling pathway*, *extracellular matrix organization*, and *endothelial cell chemotaxis*. Our study revealed that knowledge of endometrium biology in endometriosis is fragmented due to heterogeneity of published data. However, 15 genes reported as dysregulated by at least two studies within the same phase and 33 significantly enriched GO-BP terms/KEGG pathways associated with different phases of the menstrual cycle were identified.

**Conclusions:**

A multi-omics insight into molecular patterns underlying endometriosis could contribute towards identification of endometrial pathological mechanisms that impact fertility capacities of women with endometriosis.

**Electronic supplementary material:**

The online version of this article (10.1007/s10815-020-01833-3) contains supplementary material, which is available to authorized users.

## Introduction

Endometriosis is a common disease where tissue similar to normal endometrium (ectopic endometrium) grows outside of the uterus. Most commonly affected sites are the pelvic peritoneum, ovaries, uterosacral ligaments, pouch of Douglas, and the rectovaginal septum [1]. Endometriosis affects approximately 10% of women in their reproductive age. The condition is more common in women suffering with chronic pelvic pain and infertility. Although associated with several shortcomings, the most commonly used classification system is the revised American Society for Reproductive Medicine classification [2]. According to this, endometriosis is classified into sub-phenotypic stages from I to IV (minimal–severe), based on lesion number and size, presence of adhesions, and ovarian vs peritoneal involvement [3]. The diagnosis is made by direct surgical visualisation with histological confirmation of the endometrial tissue in biopsied lesions [4]. The nature of disease is often progressive, with gradually worsening pain which can lead to absence from social and work obligations [5]. This can result in significant burden to healthcare systems [6]. Non-invasive diagnostic testing could provide earlier diagnosis and improve disease management for these women [7]. Measurable biologic markers (biomarkers) from eutopic endometrium (an innermost lining layer of the uterus) and body fluids are currently the most promising non-invasive diagnostic approaches [8].

The most widely accepted aetiology of endometriosis is Sampson theory [9], whereby retrograde menstrual bleeding from the Fallopian tubes to the peritoneal cavity leads to migration, attachment, and growth of endometrial tissues at ectopic sites. However, retrograde menstrual bleeding by itself does not always lead to development of endometriosis, and multiple dysregulated mechanisms in the eutopic endometrium potentially govern pathophysiology in endometriosis. These include altered processes of apoptosis, immunosurveillance, adhesion, steroid responsiveness, tissue remodelling, neovascularisation, and enhanced inflammatory response [10], thus providing a source for biomarker identification [11].

The endometrium periodically undergoes morphological changes caused by fluctuations in the ovarian steroid hormones. Each menstrual cycle starts with the M-phase (days 1–4 in a normal 28-day cycle), characterised by shedding of the endometrial tissue. This is followed by the P-phase (days 4–14 in a normal 28-day cycle), during which higher levels of oestrogen stimulate the proliferation of the stroma and glands, resulting in thickening of the endometrium. After ovulation, progesterone levels start to rise in the ES-phase (days 15–20 in a normal 28-day cycle), stimulating the glands to secret glycogen and mucus. In the MS-phase (days 21–24 in a normal 28-day cycle), the window of implantation (WOI) opens, representing the optimal time for blastocyst implantation. This coincides with the differentiation of the endometrial stromal cells into decidual cells, known as decidualisation. In the absence of pregnancy, the endometrial tissue starts to degrade in the LS-phase (days 25–28 in a normal 28-day cycle), [12–14]. Therefore, changes in the endometrium through the menstrual cycle phases are reflected in the changes in transcriptome [15–17] and regulation patterns [18]. Consequently, differentially expressed transcripts or proteins may shed light on the important biological processes occurring at the time endometrial biopsy samples are collected. Integrating multi-omics data can provide insights into the underlying physiological and pathophysiological mechanisms [19].

Due to endometrial periodical transitions through the cycle, endometrial dating of tissue biopsies from women with and without endometriosis may play an important role in identification of the molecular patterns specific for endometriosis [20]. Although candidate endometrial biomarkers from genome-wide studies associated with endometriosis were reviewed [21], dysregulated molecular patterns of eutopic endometrium throughout the menstrual cycle among studies have not yet been integrated. Data synthesis of differentially expressed transcripts and proteins may provide an additional insight into endometrial molecular signature in women with endometriosis.

The aim of this study was therefore to (1) screen for published genome-wide studies reporting genetic causes distinguishing eutopic endometrium between women with and without endometriosis, (2) develop a catalogue of genetic causes (mRNAs, ncRNAs, and proteins) associated with altered molecular patterns in eutopic endometrium in women with endometriosis, (3) modify the gene nomenclature of extracted loci according to the HGNC system, (4) sort genes according to the phases of the menstrual cycle, and (5) perform gene set enrichment analysis (GSEA) associated with each phase of the menstrual cycle.

## Material and methods

**Fig. 1:**
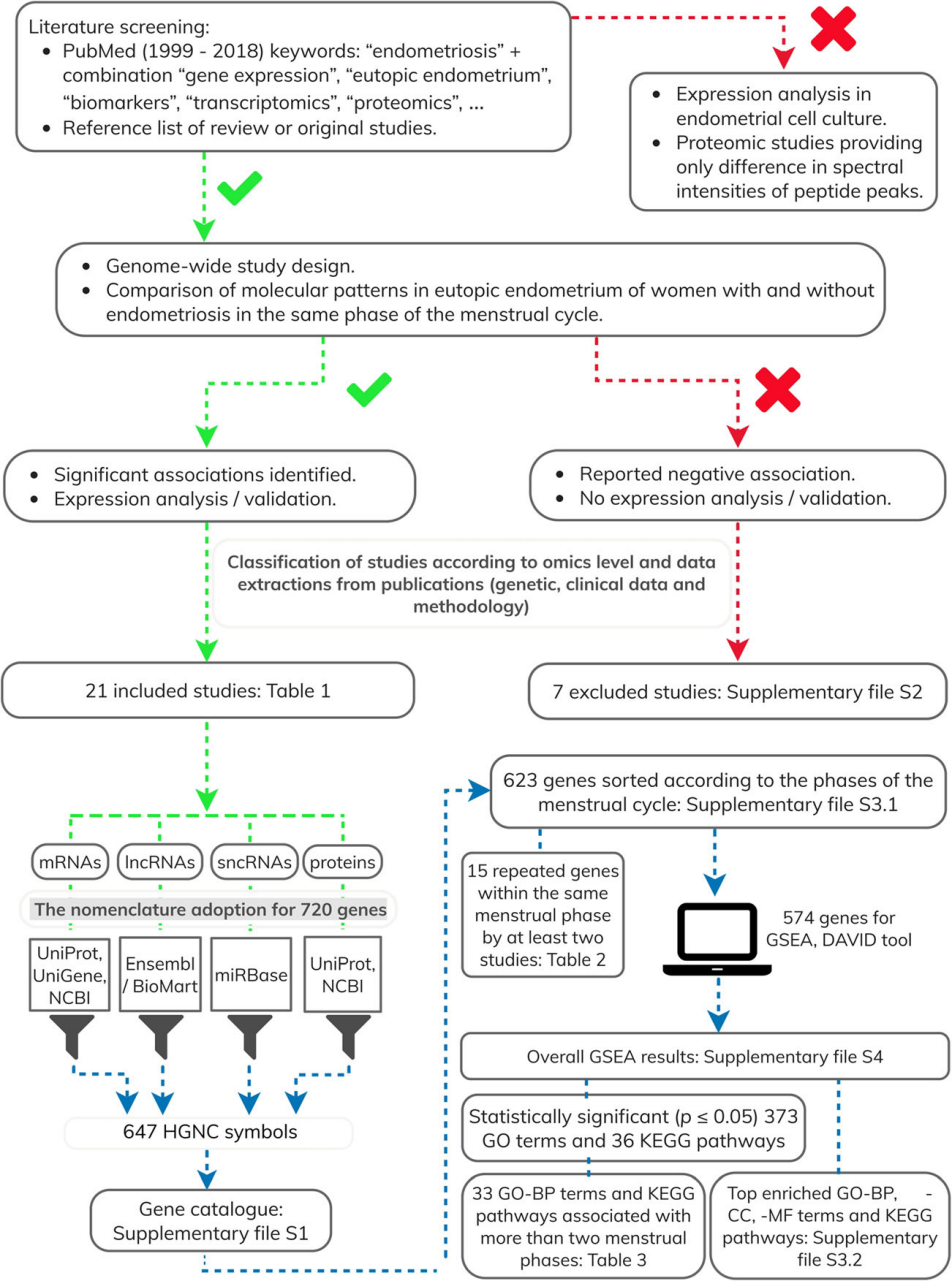
Workflow of the study

Workflow of the study is presented in Fig. 1**.**

### Literature screening and data extraction

Original publications with genome-wide approach were retrieved from PubMed database [22] using keyword “endometriosis” with combination of terms including “eutopic endometrium”, “molecular dysregulation”, “gene expression”, “transcriptomics”, “omics”, “proteomics”, “epigenomics”, “genomics”, and “biomarkers”. The time span of the literature screening was set from 1999 to December 2018. Some studies were additionally identified using the reference list of review or retrieved articles. We performed literature screening for studies that compared eutopic endometrium of women with and without endometriosis in the same phase of the menstrual cycle. We screened for studies performed at the genomic (DNA), transcriptomic (mRNAs, miRNAs, lncRNAs), proteomic, and epigenomic levels. Proteomic studies including the measuring of different spectral intensities of the peptide peaks and the peaks which could not be associated with candidate proteins were excluded. High-throughput data obtained from in vitro experiments using treated primary human endometrial cells were also excluded. Only studies published in English language were included.

The following data was extracted from publications: indications for endometrial tissue sample collection for case and control study groups, stage and type of endometriosis and other uterine/pelvis pathologies of the case group, gynaecological condition of women in the control group, clinical symptoms of pelvic pain and/or infertility for both groups, age of participants, endometrial dating (phase of the menstrual cycle) and number of endometrial tissue samples used for genome-wide analysis, procedures for processing of endometrial tissue samples (collection, storage, and nucleic acid/protein isolation), platform used for genome-wide analysis, cut-off values for expression/methylation change identification, genetic causes associated with endometriosis, determined expression/methylation fold change of transcripts/proteins, and corresponding significance values.

### Adoption of HGNC gene nomenclature for the development of the catalogue

The gene catalogue was developed from the extracted data of published studies associated with altered expression at the RNA and protein levels in eutopic endometrium of endometriosis. Up to 15 differentially expressed (either up- and down-regulated) transcripts (mRNAs, lncRNAs, and sncRNs) and proteins associated with specific phase of the menstrual cycle, fold change of expression with statistical significance value if available were extracted from publications.

The nomenclature of genes coding for transcripts and proteins reported to be dysregulated in endometriosis was adopted according to the HGNC nomenclature system (version updated February, 25th 2019). HGNC database (https://www.genenames.org/) is the resource for approved human gene nomenclature [23]. In addition, corresponding Gene ID numbers were obtained from the National Centre for Biotechnology Information (NCBI) database, release 230. NCBI is the resource that provides biological information and data [22]. Workflow of nomenclature editing for each type of extracted loci is described further. The identification name or synonym for each extracted mRNA transcript was entered into the UniProt Knowledgebase (UniProtKB), release 2019_2, and/or HGNC database. UniProtKB (https://www.uniprot.org/) is a source of sequences and annotations for over 120 million proteins [24] which also provide web links to HGNC database. Gene symbols for transcripts marked with the expression sequence tag (EST) clusters with prefix “Hs.” (*Homo sapiens*) were retrieved from NCBI’s UniGene database. In July 2019, UniGene web pages retired, but UniGene cluster numbers are matched with gene records. For example, NCBI Gene database (https://www.ncbi.nlm.nih.gov/gene) provided *BPIFB1* for “Hs.65551” that was extracted from the study performed by Burney et al. [25]. To obtain gene symbols for lncRNAs, the SeqName IDs of lncRNA transcripts were uploaded into the Ensembl/BioMart tool (version 96) (https://www.ensembl.org/biomart/martview/). BioMart is a web-based tool that provides access to the gene annotation of Ensembl data [26]. For example, Ensembl/BioMart returned *MAP4K3-DT* gene for the transcript “ENST00000451547”, obtained from the study performed by Wang et al. [27]. To obtain the official gene symbols for miRNAs, the MiRBase MIMAT accession number or miRNAs strand-specifying -3p and -5p suffixes of each miRNA transcript was entered into the miRBase database, release 22. The miRBase database (http://www.mirbase.org/) provides microRNA sequences and annotation [28]. In cases where the mature miRNA without an available ID number was obtained from the reference source, gene names for both stem loop sequences were included in the gene catalogue as they result in the mature miRNA with the same sequences. For example, extracted transcript “hsa-miR-138-5p” from the study performed by Zhou et al. [29] provided *MIR138-1* and *MIR138-2* genes by the miRBase. Synonyms, names, or UniProt accession IDs of proteins were entered into the UniProtKB database to retrieve the gene symbol. When GenInfo Identifier or “GI” number was available, then the NCBI protein database was used to obtain gene symbol or Gene ID number. For example, extracted “gi|825,671” from the study performed by Rai et al. [30] was entered into NCBI protein database, which further provided web link to the HGNC database from where *NPM1* gene was obtained.

### Gene set enrichment analysis

All genes from developed catalogue regardless of clinical and experimental characteristics from extracted studies were further sorted according to the phases of the menstrual cycle (M, P, S, ES, MS, and LS) and N/S, when information regarding endometrial dating was missing. Extracted transcripts and proteins with no confirmed differences in the expression levels after validation of the source references were excluded from gene sorting. Gene set enrichment analysis (also named functional enrichment analysis) was performed for each list of genes associated with specific phases of the menstrual cycle using the Database for Annotation, Visualization and Integrated Discovery (DAVID) Bioinformatics Resource (release 6.8). DAVID is a bioinformatics tool that accepts gene list and performs functional analysis using background algorithms and knowledge of annotation databases, including Kyoto Encyclopedia of Genes and Genomes (KEGG), BioCarta, Enzyme nomenclature database and Reactome, to understand biological meaning behind the genes of interest [31]. In the present GSEA, Gene Ontology (GO) terms such as biological process (BP), cellular component (CC), molecular function (MF), and KEGG pathways specific to *Homo sapiens* with a *p* ≤ 0.05 were considered as statistically significant. GO [32] and KEGG [33] are databases that collect knowledge regarding the function of gene products and their roles in the biological system and phenotypes.

## Results

**Fig. 2:**
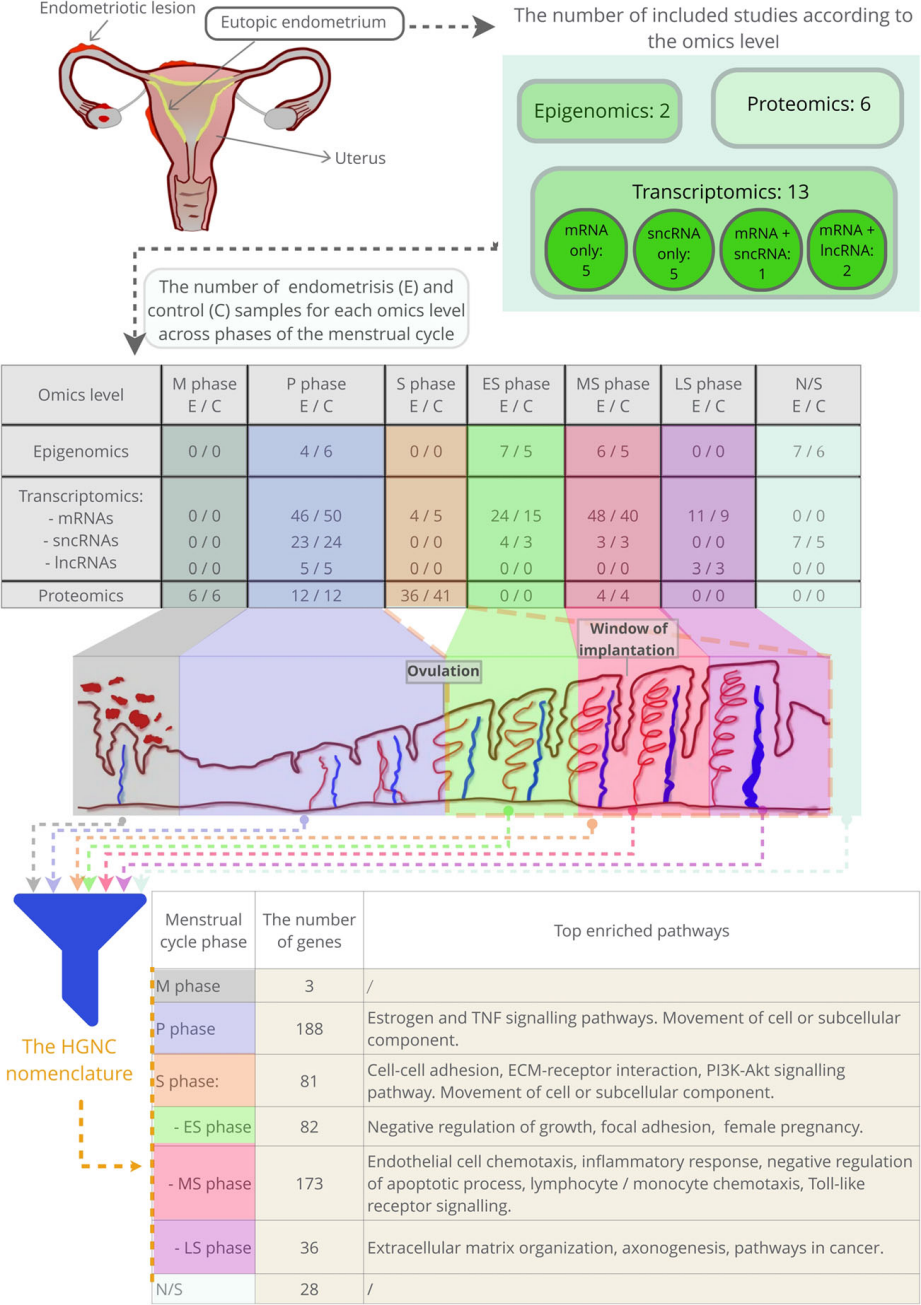
Study overview and main results. Different omics levels of studies that analysed eutopic endometrium in women with and without endometriosis. Identified enriched pathways associated with the phases of the menstrual cycle characterising eutopic endometrium of endometriosis

The main findings of the present study are overviewed in Fig. 2.

### Classification of retrieved studies

**Fig. 3:**
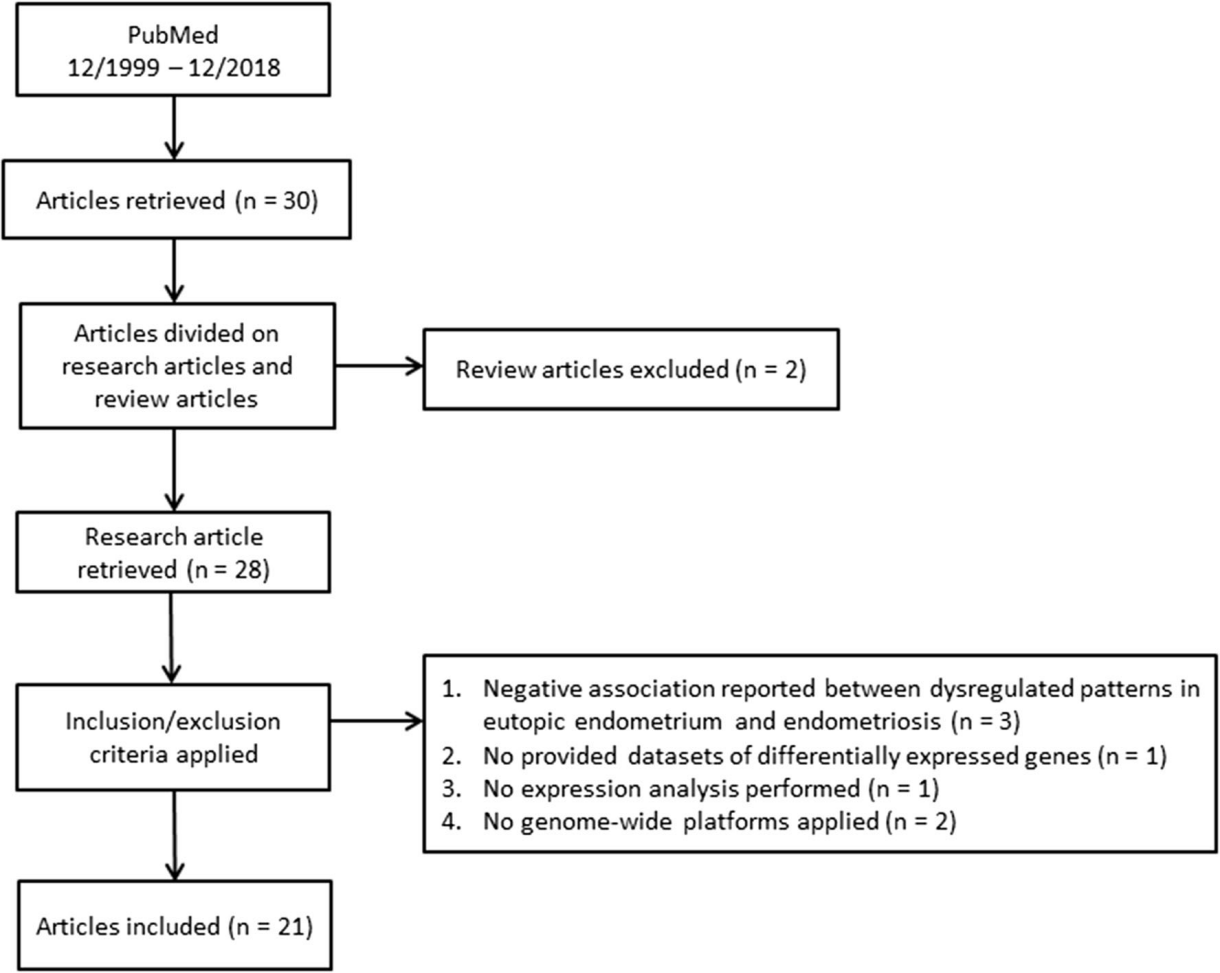
Workflow of the database mining

The database mining (Fig. 3) provided 28 genome-wide studies that analysed the eutopic endometrium in women with and without endometriosis. The analysis of the obtained data revealed that datasets are heterogeneous and included dysregulation of expression patterns at the transcriptomics and proteomics levels. Additionally, some of the transcriptomics studies reported expression of both mRNAs and ncRNAs (sncRNAs or lncRNAs); therefore, these datasets were considered as transcriptomics and ncRNomics studies. Some retrieved studies reported dysregulation of sncRNAs only, and therefore these studies fell into both the transcriptomics and ncRNomics levels. Some studies also overlapped with epigenomics, because they reported aberrant DNA methylome and associated gene expression levels.

**Fig. 4:**
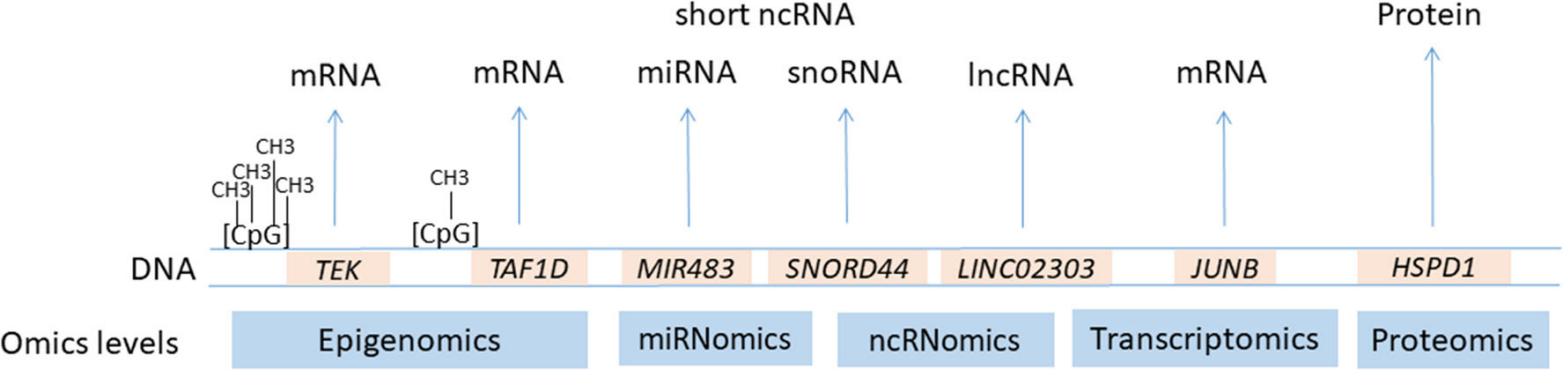
Omics levels of extracted data used for the development of the gene catalogue. CpG islands present sites of methylation. mRNA, messenger RNA; miRNA, micro RNA; snoRNA, small nucleolar RNA; lncRNA, long non-coding RNA

Out of 28, 21 studies that reported an association of altered molecular patterns related to eutopic endometrium with endometriosis were further used for the development of the catalogue. Eight out of 21 studies were performed at the transcriptomics level: five [25, 34–37] profiled only mRNAs, two [27, 38] profiled mRNAs and lncRNAs, and one [29] reported dysregulation of mRNAs and sncRNAs (miRNAs and snoRNAs). Five additional studies analysed dysregulation of sncRNAs only [39–43]. Six [30, 44–48] out of 21 studies were performed at the proteomics level. Additionally, we also included differentially expressed protein-coding genes from two epigenomics studies [49, 50], which reported altered mRNA expression levels of differentially methylated genes. Studies included in the present analysis are listed in Table 1**.** Figure 4 presents data extraction (differentially expressed transcripts and proteins) for multi-omics integration.

**Table 1:** Clinical and experimental characteristics of included studies

Reference	Indication for endometrial tissue sample collection	Number of endometriosis (E) samples used for genome-wide analysis in studied phase of the cycle. Stage and type of E	Number of control samples used for genome-wide analysis in studied phase of the cycle. Phenotype	-Endometrial tissue collection -Sample preservation -Nucleic acid/protein isolation	Platform for genome-wide analysis	Cut-off for fold change (FC) or *p* value and genetic causes associated with E
Epigenomic level						
[49]	Laparoscopy	N/S = 7 in reproductive age. Stage E: N/S	N/S = 6 in reproductive age	-N/S -RNAlater -Genomic DNA: Qiagen DNeasy Blood & Tissue Kit. Total RNA: TRIzol Reagent, purified by the Qiagen RNeasy MinElute Cleanup Kit	-Illumina Infinium Human Methylation 27 K RevB Beadchip -qRT-PCR (validation)	FC ≥ 1.5: 59 hypermeth. genes and 61 hypometh. genes *p* < 0.05 (validation)
[50]	Laparoscopy for endometriosis-related pain and/or infertility (cases). Endometrial biopsy, hysterectomy, or gynaecologic surgery for benign condition (controls)	P = 4 (39 ± 9 years), ES = 7 (35 ± 3 years), MS = 6 (34 ± 10 years). Stage IV*E	P = 6 (43 ± 1 years), ES = 5 (41 ± 3 years), MS = 5 (46 ± 1 years)	-N/S -N/S -Genomic DNA: NucleoSpin Tissue Kit. Total RNA: DNase treatment by Qiagen RNeasy Plus Kit	-Illumina Infinium Human-Methylation27K -Affymetrix HU133 Plus 2.0 (validation)	*p* < 0.05: P: 58 DMCs corresponding to 58 loci. ES: 39 DMCs corresponding to 36 loci. MS: 137 DMCs corresponding to 125 loci. Spearman correlation (validation)
Transcriptomics level (only protein-coding mRNAs)						
[25]	Normally cycling women underwent laparoscopy (cases), hysterectomy, or endometrial biopsy (controls)	P = 6 (35 ± 5 years), ES = 6 (32 ± 6 years), MS = 9 (35 ± 6 years). Stage III/IV. Surgery and histologically confirmed E: ovarian/peritoneal E (*n* = 5), ovarian/peritoneal E+ leiomyoma (*n* = 2), peritoneal E (*n* = 1), peritoneal + liver E (*n* = 1), rectovaginal/ovarian/peritoneal E (*n* = 4), rectovaginal/ovarian/peritoneal E + leiomyoma (n = 1), rectovaginal/peritoneal E (*n* = 3), rectovaginal/peritoneal E + leiomyoma (*n* = 4). Many infertile with previous failed ART treatments	P = 5 (37 ± 7 years), ES = 3 (46 ± 2 years), MS = 8 (37 ± 8 years). Uterine prolapse (n = 3), uterine leiomyomata (n = 5), pelvic pain (n = 1), and normal volunteers (*n* = 7). Laparoscopy proven without E	-Pipelle catheter or uterine curetting -Liquid N_2_ -Total RNA: TRIzol reagent, DNase treatment, and purified by the Qiagen RNeasy Kit	Affymetrix HU133 Plus 2.0	FC > 1.5, *p* < 0.05: P: 278↑/461↓ ES: 986↑/2321↓ MS: 430↑/315↓
[34]	Endometrial biopsy or hysterectomy	P = 6, S = 4. Aged 19–48 years. Stage: N/S	P = 5, S = 5. Aged 19–48 years.	-N/S -Collected in Moscona solution, frozen in liquid N_2_ -Total RNA: a Qiagen RNeasy Kit	Affymetrix Human Genome U95A	FC ≥ 1.5: P: 29↓/9↑ S: 35↓/23↑
[36]	Normally cycling women underwent endometrial biopsy	LS = 8. Stage I/II (*n* = 4) and III/IV (*n* = 4). Laparoscopically proven endometriosis	LS = 6. Laparoscopically proven without E	-Pipelle catheter -Liquid N_2_ -Total RNA: TRIzol reagent	Microarray Core Facility custom made array	FC >1.75, *p* < 0.01: 8↑ / 1↓
[37]	Archived endometrial samples provided by UCSF Human Endometrial Tissue Bank from normally cycling women underwent procedures for diagnosis and treatments of pelvic pain, infertility or benign gynaecologic conditions, and normal volunteers	Minimal/Mild E: P = 11 (37 ± 5 years), ES = 6 (37 ± 6 years), MS = 10 (36 ± 8 years). Stage I/II. Peritoneal endometriosis concurrent with fibroids (*n* = 7) and/or adhesions (*n* = 2), chronic pelvic pain (*n* = 21), dysmenorrhea (*n* = 3), and/or infertility (*n* = 4) Moderate/Severe E: P = 18 (36 ± 7 years), ES = 12 (35 ± 6 years), MS = 18 (34 ± 7 years),“indeterminate” cycle phase = 1. Stage III/IV. Extensive endometriosis with concurrent adhesions (*n* = 25), fibroids (*n* = 10) chronic pelvic pain (*n* = 36), adenomyosis (*n* = 1) and/or infertility (*n* = 6)	Group “healthy non-E”: P = 20 (32 ± 5 years), ES = 6 (32 ± 3 years), MS = 8 (33 ± 4 years). Healthy with abdominal pain/pyloric stenosis (*n* = 2), desired (*n* = 9) / undesired (*n* = 7) future fertility, unexplained pelvic pain (*n* = 1) or infertility (*n* = 3), normal volunteer (*n* = 7), egg donor in natural cycle (*n* = 5). Group “non-E with other uterine/pelvic pathology”: P = 15 (43 ± 5 years), ES = 6 (42 ± 6 years), MS = 14 (43 ± 6 years). Symptomatic uterine fibroids (*n* = 16), adenomyosis (*n* = 3), chronic pelvic pain (*n* = 3), uterine (*n* = 3)/uterovaginal (*n* = 1)/pelvic organ (*n* = 3)/prolapse, adhesions (*n* = 2), dysmenorrhea (*n* = 4), endometrial polyp (*n* = 1), cystocele (*n* = 3), stress urinary incontinence (*n* = 3), menorrhagia (*n* = 2) or benign ovarian cyst (*n* = 1)	-Pipelle catheter or uterine curetting -Liquid N_2_ -Total RNA: TRIzol reagent	Affymetrix HU133 Plus 2.0	FC > 1.5: “Min./Mild+Mode./Severe E” vs. healthy non-E”: P: 7573↑/11,866↓. ES: 2905↑/7966↓. MS: 4020↑/6438↓. “Min./Mild+Mode./Severe E” vs. non-E + uterine/pelvic pathology: P: 2↑ / 85↓. ES: 12↑ / 55↓. MS: 31↑ / 74↓.
[35]	Normal cycling women underwent endometrial biopsy	MS aged 28–39 years (LH + 6–LH + 10 timed to expected WOI) = 8. Stage II/III. Surgically confirmed pelvic E	MS aged 28–39 years (LH + 6–LH + 10 timed to expected WOI) = 7. Surgically confirmed without E	-Pipelle catheter -Liquid N_2_ -Poly (A) + RNA: Oligotex Direct mRNA isolation kit	Affymetrix Genechip Hu95A	FC ≥ 2, *p* < 0.05: 91↑/115↓
Transcriptomics (protein-coding genes and ncRNAs)						
[27]	Normal cycling women underwent total hysterectomy	LS = 3. Stage: N/S.	LS = 3. Normal endometrium without oestrogen-dependent disease	-N/S -Snap-frozen in liquid N_2_, stored at -80 °C -Total RNA: TRIzol reagent	Human lncRA Expression Microarray V3.0	FC > 2.0: 578↑/638↓ (mRNAs) 488↑/789↓ (lncRNAs)
[38]	Normal cycling women underwent eutopic endometrial samples collection	P = 5. Stage III/IV. Laparoscopically diagnosed E	P = 5. Without visible E	-N/S -N/S -RNA: TRIzol reagent	Illumina HiSeq 2500 with 150 bp paired-end reads provided 1,045,089,518 clean reads	*p* < 0.05: 753↑/475↓ (mRNAs) 33↑/53↓ (lncRNAs)
[29]	Normal cycling women underwent hysteroscopy.	MS = 3 aged 20–35 years. Stage I/II. Laparoscopically confirmed.	MS = 3 aged 20–35 years. Laparoscopically proven without E.	-N/S -Liquid N_2_ -Total RNA: TRIzol reagent	-Agilent Human 4 × 44 K -miRCURY LNA microRNA array	FC > 2.0: 224↑/133↓ (mRNAs) FC ≥ 2.0: 54↑/12↓ (miRNAs)
Transcriptomics (only ncRNAs)						
[42]	Laparoscopy for non-malignant ovarian lesions (cases) or infertility work-up or removal of simple ovarian cysts (controls)	P = 10 aged 20–35 years. Stage III/IV. Ovarian E confirmed by laparoscopy and histopathology with some concurrent primary/secondary infertility	P = 10 aged 20–35 years. Primary/secondary infertility or with simple ovarian cysts	-Pipelle suction catheter -RNAlater, stored at −80 °C -Total RNA: mirVana miRNA Isolation Kit	TaqMan Array Human MicroRNA A v2.1 + B v2.0 Cards	FC ≥ 2.0 or *p* ≤ 0.1: 2↑/13↓
[41]	Normal cycling women underwent laparoscopy for adnexal mass or infertility	Mean age 31.4 ± 0.9 years of a total 21 women. P = 10. Stage III/IV. Ovarian E confirmed by laparoscopy and histopathology with some concurred primary/secondary infertility	Mean age 30.7 ± 0.9 years of a total 25 women. P = 11. Primary/secondary infertility or with simple ovarian cysts	-Pipelle catheter -RNA later, stored at −80 °C -Total RNA: mirVana miRNA Isolation Kit	Exiqon’s miRCURY LNA microRNA Array 7th	Adjusted *p* values < 0.05: 1198 dysregulated miRNAs
[40]	Normal cycling women underwent laparoscopy for endometriosis treatment and hysterectomy for uterine leiomyomata	ES = 4 (28 ± 7 years). Stage III/IV. E confirmed by laparoscopy and histology: rectovaginal/peritoneal E + leiomyomata (*n* = 1), ovarian/peritoneal/rectovaginal E (*n* = 2), ovarian/peritoneal E (*n* = 1)	ES = 3 (45 ± 3 years). Uterine leiomyomata, none of which submucosal in location. No pathological evidence of inflammation in sampled endometrium	-Pipelle catheter or uterine curettage -N/S -Total RNA: TRIzol reagent	Exiqon’s miRCURY LNA array v. 10.0	FC > 1.5 and FDR < 0.05: 6↓
[43]	Pre-menopausal women underwent laparoscopy.	P = 3 (37 ± 5 years). Stage E: N/S.	P = 3 (39 ± 5 years)	-Uterine curettage -N/S -Total RNA: TRIzol reagent	Exiqon’s miRCURY LNA microRNA array v. 14.0	FC > 2.0: 36↓
[39]	Laparoscopy for endometriosis treatments due to abdominal pain (74.5%) and sterility (25%), or tubal sterilisation (controls)	N/S = 7 (aged 20–45 years). Stage E: N/S. E confirmed by laparoscopy and histology. No concurrent other pelvic pathology.	N/S = 5 (aged 27–45 years). Surgical confirmed without E or negative diagnosis of biopsies of suspicious areas of endometriosis.	-Suction cannula -Rinsed in PBS, stored in liquid N_2_ -Total RNA: mirVana miRNA isolation kit	Affymetrix GeneChip miRNA 2.0. Array	p < 0.05: 11↑ / 9↓
Proteomics level						
[48]	Laparoscopy	S = 6 in reproductive age. Stage II (*n* = 2), stage III (*n* = 2), stage IV (*n* = 2). E confirmed by laparoscopy and pathology	S = 6 in reproductive age	-N/S -Washed in PBS, grounded into powders in liquid N_2_ -Benzonase DNase for DNA and RNA removal, the homogenates centrifuged, the supernatant precipitated with acetone and re-suspended	2-DE IEF, SDS-PAGE + MALDI-TOF-MS	FC ≥ 3.0: 11 DEPs
[46]	Laparoscopic sterilisation or assessment of tubal patency	MS = 4 in reproductive age. Stage I (*n* = 2), stage II (*n* = 1), stage IV (*n* = 1)	MS = 4 in reproductive age. Healthy with unknown medical condition	-Uterine curettage -Snap frozen on dry ice, stored at −80 °C -Homogenised, pelleted and re-homogenised. Proteins precipitated with acetone	2D-DIGE + MALDI- TOF-MS, MS/MS	p < 0.05: 20 DEPs
[30]	Diagnostic procedure for infertility, tubal re-enastomosis, or pelvic pain	Mean age 27.5 ± 4.4 years of a total 57 women. P = 6: only stage IV. S = 18: Stage II (*n* = 6), stage III (*n* = 6), and stage IV (*n* = 6). Laparoscopically proven different types of E: pelvic lesions, adhesions or endometrioma	Mean age 26.7 ± 3.9 years of a total 59 women. P = 6, S = 18. Normal menstrual cycle and hormone profiles, without uterine abnormalities	-N/S -Frozen in liquid N_2_, stored at −80 °C -Lysed, homogenised, and supernatant precipitated with trichloroacetic acid /acetone. Pellet re-suspended in lysis buffer	2DE-PAGE + MALDI MS and/or MS/MS	FC ≥ 1.2, *p* < 0.05: 48 DEPs
[47]	Routine laparoscopy for unexplained infertility, tubal re-enastomisis or pelvic pain	S = 6 (40 ± 5 years). Stage II. Laparoscopically and histologically diagnosed E for the first time or receiving recurrent treatment for pre-existing E. Concurrent with amenorrhea and pelvic pain (*n* = 1), infertility and removal of uterine septum (*n* = 1), previous dermoid cyst (*n* = 1), hysterectomy due to fibroid and menorrhagia (*n* = 1), infertility (*n* = 1), infertility, bowel symptoms and dysmenorrhoea (*n* = 1)	S = 11 (33 ± 7 years). Asherman’s syndrome (*n* = 1), menorrhagia and fibroids (*n* = 1), dysmenorrhoea (*n* = 1), dermoid cyst (*n* = 1), intramenstrual bleeding (*n* = 1), pelvic pain (*n* = 1), and unknown symptoms (*n* = 5)	-Uterine curettage -Frozen in liquid N_2_, stored in −80 °C. -Powder of tissue lysed, sonicated and centrifuged to discard the pellet. Supernatant precipitated with acetone and pellet further re-suspended	SDS-PAGE + MALDI-TOF-MS	*p* < 0.05: 21 DEPs
[44]	Laparoscopy for diagnostic purpose, including infertility, treatment of endometriosis or elective tubal sterilisation	P = 6 (27 ± 3 years), S = 6 (30 ± 4 years). Stage: N/S. Laparoscopically confirmed E with no coexistent condition or endometrial pathology.	P = 6 (29 ± 5 years), S = 6 (28 ± 3 years). Healthy.	-Pipelle catheter -Washed in isotonic PBS, frozen in liquid N_2_, stored in −80 °C -Lysed, sonicated, and centrifuged to remove insoluble material	2-DE-IPG, PAGE + MALDI-TOF-MS	FC ≥ 3.0: P: 38↑/13↓ S: 60↑/32↓
[45]	Collection of menstrual blood	M = 6 (aged 25–40 years). Stage: N/S. Laparoscopic diagnosis of advanced E	M = 6 (aged 25–40 years) No E, adenomyosis or leiomyoma	-Suction catheter -Cultured in DMEM and FBS for 2 days at 37 °C, 5% CO2. Washed 3-times with PBS, re-suspended in DMEM with 10% FBS, after 2 weeks collected endometrial cells -Cell lysis, centrifugation for supernatant.	2-DE + ESI-Q-TOF/MS	FC ≥ 3.0: 3↓

*Endometriosis severity stages I–IV was determined according to the revised classification by American Fertility Society (AFS) and American Society for Reproductive Medicine (ASRM)

*Hypometh*, hypomethylated; *Hypermeth*, hypermethylated; *DMCs*, differentially methylated CpGs; *N/S*, not specified in the source reference; ↑, up-regulated; ↓, down-regulated; *N*_*2*_, nitrogen; *E*, endometriosis; *C*, controls; *P*, proliferative; *S*, secretory; *ES*, early-secretory; *MS*, mid-secretory; *LS*, late-secretory; *PBS*, phosphate-buffered saline; *MALDI-TOF-MS*, matrix-assisted laser desorption ionisation/time-of-flight-mass spectrometry; *SELDI-TOF-MS*, surface enhanced laser desorption/ionisation time-of-flight-mass spectrometry; *2D DIGE*, two dimensional-differential in gel electrophoresis; *IEF*, isoelectric focusing; *IPG*, immobilised pH gradient; *SDS-PAGE*, sodium dodecylsulfate-polyacrylamide gel electrophoresis; *DEPs,* differentially expressed proteins

Out of 28, seven studies were not included in the catalogue development because they did not meet the inclusion criteria of the present study (no expression analysis or data not available, expression analysis performed using separated cell types, platforms with limited number of target genes, and negative association reported), [51–57]. Although not included in the developed catalogue, these studies contribute in understanding of endometrial biology in endometriosis and are therefore summarised in Supplementary file S2.

### Description of the gene catalogue

From 21 publications, 760 dysregulated transcripts (mRNAs, lncRNAs, and sncRNAs) and proteins in endometriosis were extracted and were used for the gene catalogue development. Among 760 genetic causes, 37, 425, 169, and 129 were associated with endometriosis at the epigenomics, transcriptomics, ncRNomics, and proteomics level, respectively. The gene catalogue is provided in Supplementary file S1. After nomenclature editing according to HGNC database, 647 official gene symbols were retrieved. The official gene coding for 113 dysregulated transcripts/proteins is currently not available. Among 647, 24 genes were excluded since additional validation analysis using qPCR, immunohistochemistry, or western blot did not confirm genome-wide screening findings. Therefore, the final set of genes included 623 genes: 33, 368, 103, and 119 genes associated with endometriosis at the epigenomics, transcriptomics, ncRNomics, and proteomics level, respectively. Sorting of these 623 genes according to phases of the menstrual cycle resulted in 3, 200, 87, 87, 181, 37, and 28 genes associated with M-, P-, S-, ES-, MS-, LS-phase, and N/S, respectively. Analysis of the catalogue revealed that same genes were associated with different menstrual phases or were associated with the same phase in different studies. Among 623 genes, 72 genes were repeated 2 times (55 genes) or at least 3 times (17 genes) in the same or in different phases of the menstrual cycle. For example, *FOS* was associated with the MS-phase by two different studies [29, 37] as well as with S- [34], P-, and ES-phases [37]. Eleven genes were repeated within the same menstrual phase in the same study. For example, two transcripts of *SNORD3A* gene were reported by Cui et al. [38] and two isoforms of the VIM protein were shown to be up- and down-regulated [30]. Among 72 repeated genes, there are 61 genes that were repeated in different phases of the menstrual cycle. For example, down-regulated *MIR374B* was associated with P-phase [43] and with MS-phase [29]. A list of 72 repeated genes with associated phase of the menstrual cycle, omics level, and source reference are presented in Supplementary file S5. Table 2 includes 15 genes associated with the same phase of the menstrual cycle by more than one study, for example up-regulated *CCN1* in the MS-phase [29, 37]. Removing duplicated genes from the same phase of the menstrual cycle resulted in 591 unique phase-specific genes, i.e. M = 3, P = 188, S = 81, ES = 82, MS = 173, LS = 36, and N/S = 28 (Supplementary file S3.1).

**Table 2:** Genes associated with the same phase of the menstrual cycle in at least two studies

The phase of the menstrual cycle	P-phase	S-phase	ES-phase	MS-phase	LS-phase
Repeated gene symbols and source references	*ACTB* [30, 44],	*ACTB* [30, 44],	*ANLN* [25, 37].	*CCN1* [37, 29],	/
	*ANXA4* [30, 34],	*HSP90AB1* [44, 47],	/	*CRISP3* [25, 37],	/
	*BPIFB1* [25, 38],	*VIM* [30, 47, 44].	/	*EGR1* [37, 29],	/
	*EPHX1* [34, 37],	/	/	*FOS* [29, 37],	/
	*MUC5B* [34, 37, 38],	/	/	*FOSB* [29, 37],	/
	*PRDX2* [30, 44],	/	/	*TRPM6* [25, 37].	/
	*VIM* [30, 44].	/	/	/	/

### Gene set enrichment analysis

A total of 591 unique genes sorted according to the phases of the menstrual cycle were used for GSEA. A set of 574 genes was identified by DAVID bioinformatics tool. The number of genes included in the GSEA and results of functional analysis are summarised in Table 3 (GO-BP, -CC, -MF terms, and KEGG pathways). The highest number of statistically significant enriched terms was obtained by GO-BP analysis (207), followed by GO-CC (93), GO-MF (73), and KEGG (36), however the majority of identified enriched pathways do not reach the Bonferroni correction value. The top 10 enriched GO-BP, -CC, -MF, and KEGG pathways terms with corresponding annotated genes ranked by lowest *p* values and Bonferroni correction for each phase of the menstrual cycle is listed in the Supplementary file S3.2, while a complete list of GSEA results is provided in the Supplementary file S4. The pathway most significantly associated with P- (*p =* 5.25E-07) and S- (*p =* 8.11E-08) phases was *movement of cell or subcellular component*. In addition, *oestrogen signalling pathway* (*p* = 0.03) and *TNF signalling pathway* (*p* = 0.04) were associated with P-phase. *PI3K-Akt signalling pathway* (*p =* 1.47E-05) was enriched by genes associated with the S-phase. *Negative regulation of growth* (*p* = 6.41E-05) was associated with the ES-phase. *Endothelial cell chemotaxis* (*p* = 3.85E-05), *inflammatory response* (*p* = 4.50E-05), and *chemokine-mediated signalling pathway* (*p* = 2.39E-04) were associated with the MS-phase. *Extracellular matrix organization* (*p* = 1.88E-04) and *pathways in cancer* (*p* = 0.03) were enriched by LS-phase gene list. The study also revealed that same pathways were associated with different phases of the menstrual cycle (Table 4). Among 243 statistically significant enriched GO-BP and KEGG pathways, 30 GO-BP and 3 KEGG pathways were associated with at least two phases. For example, *negative regulation of apoptotic process* was associated with four phases, i.e. P (*p* = 0.0004), S (*p* = 0.0001), ES (*p* = 0.0411), and MS (*p* = 0.0003).

**Table 3:** A summary of the gene catalogue and GSEA results for each phase of the menstrual cycle

	The gene catalogue			Number of genes included in the analysis and result of total and statistically significantly (*p* ≤ 0.05) enriched GO terms and KEGG pathways												
Phase of the menstrual cycle	1	2	3	4	5	6		7	8		9	10		11	12	
	All genes	Unique genes	Repeated genes in the same phase	Number of genes for DAVID analysis*	Genes included in GO-BP analysis	The number of enriched GO-BP terms	Significantly associated GO-BP terms (*p* ≤ 0.05)	Genes included in GO-CC analysis	The number of enriched GO-CC terms	Significantly associated GO-CC terms (*p* ≤ 0.05)	Genes included in GO-MF analysis	The number of enriched GO-MF analysis	Significantly associated GO-MF terms (*p* ≤ 0.05)	Genes included in KEGG analysis	The number of enriched KEGG pathways	Significantly associated KEGG pathways (*p* ≤ 0.05)
M-phase	3	3	0	0	0	0	0	3	3	3	0	0	0	0	0	0
P-phase	200	188	9	185	92	100	64	117	43	33	113	32	19	31	11	3
S-phase	87	81	4	77	60	81	57	72	49	39	71	38	30	30	15	13
ES-phase	87	82	5	81	40	40	23	69	11	8	18	8	3	13	4	2
MS-phase	181	173	8	170	90	88	55	114	18	5	109	27	16	35	18	15
LS-phase	37	36	1	33	13	11	8	18	7	5	21	7	5	7	5	3
N/S	28	28	0	28	0	0	0	0	0	0	0	0	0	0	0	0
Total	623	591	27	574	/	320	207	/	131	93	/	112	73	/	53	36

Column 1, the number of obtained gene symbols for extracted loci; Column 2, the number of unique gene symbols after removing of duplicates in the same phase; Column 3, the number of repeated genes in the same phase (some genes repeated in the same phase 2 or > 2 times); Column 4, the number of genes included in GSEA analysis by DAVID bioinformatics tool

*DAVID tool enabled analysis of all provided genes. Columns 5, 7, 9, and 11 refer to the number of genes included in enrichment analyses Columns 6, 8, 10, and 12 refer to the number of all identified enriched terms by DAVID bioinformatics tool

*GO*, Gene Ontology; *BP*, biological process; *CC*, cellular component; *MF*, molecular function; *KEGG*, Kyoto Encyclopedia of Genes and Genomes; *DAVID*, Database for Annotation, Visualization and Integrated Discovery

**Table 4:** Biological pathways associated with in at least two phases of the menstrual cycle. Only pathways with *p* ≤ 0.05 values are shown

Enriched GO-BP and KEGG pathways term	*p* values				
	P-phase	S-phase	ES-phase	MS-phase	LS-phase
GO:0001816~cytokine production	0.0178	/	/	0.0163	/
GO:0006457~protein folding	0.0037	9.9496E-07	/	/	/
GO:0006928~movement of cell or subcellular component	5.2510E-07	8.1149E-08	/	/	/
GO:0006986~response to unfolded protein	0.0466	3.0744E-05	/	/	/
GO:0007267~cell-cell signalling	0.0186	/	/	0.0152	/
GO:0007565~female pregnancy	/	/	0.0005	0.0331	/
GO:0007568~ageing	/	/	0.0050	0.0413	/
GO:0009409~response to cold	0.0352	0.0104	/	/	/
GO:0030049~muscle filament sliding	0.0389	0.0115	/	/	/
GO:0030198~extracellular matrix organization	/	2.0573E-05	/	/	0.0002
GO:0032496~response to lipopolysaccharide	6.3165E-05	0.0334	/	/	/
GO:0032570~response to progesterone	0.0408		0.0115	0.0376	/
GO:0034599~cellular response to oxidative stress	0.0157	0.0308	/	/	/
GO:0035914~skeletal muscle cell differentiation	/	0.0187	/	0.0006	/
GO:0042060~wound healing	/	0.0462	/	0.0252	/
GO:0042493~response to drug	0.0037	0.0098	0.0003	0.0330	/
GO:0043066~negative regulation of apoptotic process	0.0004	0.0001	0.0411	0.0003	/
GO:0043154~negative regulation of cysteine-type endopeptidase activity involved in apoptotic process		0.0032	/	0.0171	/
GO:0043627~response to oestrogen	0.0164	0.0317	/	/	/
GO:0045454~cell redox homeostasis	0.0004	0.0432	/	/	/
GO:0045944~positive regulation of transcription from RNA polymerase II promoter	0.0310	/	/	0.0007	/
GO:0050729~positive regulation of inflammatory response	0.0222	/	/	0.0003	/
GO:0050819~negative regulation of coagulation	0.0404	0.0213	/		/
GO:0050821~protein stabilisation	0.0009	0.0003	/		/
GO:0051591~response to cAMP	0.0064	0.0166	/	0.0056	/
GO:0051607~defence response to virus	0.0473	/	/	0.0413	/
GO:0051881~regulation of mitochondrial membrane potential	0.0220	0.0064	/		/
GO:0070527~platelet aggregation	0.0046	2.7906E-05	/		/
GO:0071356~cellular response to tumour necrosis factor	0.0129	/	/	0.0018	/
GO:1901998~toxin transport	0.0352	0.0104	/	/	/
hsa04668:TNF signalling pathway	0.0399	/	/	0.0083	/
hsa04915:Oestrogen signalling pathway	0.0312	0.0297	/	/	/
hsa05200:Pathways in cancer	/	0.0047	/	0.0164	0.0345

/, pathway not enriched; *P*, proliferative; *S*, secretory; *ES*, early-secretory; *MS*, mid-secretory, *LS*, late-secretory; *GO-BP*, Gene Ontology Biological Processes; *KEGG*, Kyoto Encyclopedia of Genes and Genomes

## Discussion

In the present study, we developed a catalogue of genes reported to have altered molecular patterns in eutopic endometrium in endometriosis. The analysis of 21 studies including 39 women with endometriosis and 236 women without endometriosis revealed that the reported datasets are heterogeneous. Obtained data from publications included dysregulated molecular patterns at diverse omics levels, i.e. transcriptomics (mRNAs, sncRNAs, and lncRNAs), proteomics, and epigenomics (dysregulated expressed genes associated with altered methylation level). The nomenclature editing of extracted data was beyond of a single omics level, therefore, the official HGNC nomenclature system for human genes was adopted which enabled downstream GSEA. Sorting of the obtained 591 unique genes resulted in 7 groups according to the phases of the menstrual cycle and further enrichment functional analysis was performed.

### The nomenclature editing and analysis of the gene catalogue

From the literature survey, we gathered mRNA and ncRNA transcripts and proteins, and developed the catalogue by manually editing their gene nomenclature. Menstrual phase–specific sorting of 623 genes revealed that 15 genes were repeated within the same phase of the menstrual cycle since dysregulated expression was reported by at least two studies (Table 2), therefore, could present stronger candidate biomarkers associated with affected endometrial function in endometriosis. Repeated genes associated with P-phase (*ACTB*, *ANXA4*, *BPIFB1*, *EPHX1*, *MUC5B*, *PRDX2*, and *VIM*) could indicate stronger genetic causes associated with pathophysiology of endometriosis. Repeated genes from the MS-phase (*CCN1*, *CRISP3*, *EGR1*, *FOS*, *FOSB*, and *TRPM6*) could be associated with affected receptivity in endometriosis. However, independent validation studies are now needed to verify these hypotheses. Most of the data in the catalogue included dysregulated mRNA transcripts which were supplemented with approved HGNC gene symbols. However, extracted data from publication also included proteins and genes coding for epigenetic regulators (sncRNA and lncRNAs) which were also supplemented by the corresponding HGNC gene symbols. In case of epigenetics marks (altered DNA methylation), gene symbol of the associated differentially expressed gene was added to the catalogue. Adopted gene nomenclature of reported RNA transcripts and proteins with altered expression levels in endometriosis at various omics levels enabled downstream analysis, since most bioinformatics tools require the input of official gene symbols. LncRNAs whose official gene symbols are not yet available, were also listed in the gene catalogue. Since the HGNC nomenclature is continuously updated, this catalogue will enable re-analysis of the gene list in the future.

### Types of omics data in the gene catalogue

Five genes (*ANXA4*, *CDA*, *CDK10*, *DST*, and *HSP90AB1*) from the catalogue were reported to be associated with endometriosis at two omics levels, for example *ANXA4* gene at transcriptomics [34] and ANXA4 at proteomics level [30]. As reported previously, there is no complete correlation between transcriptome (expressed portion of the genome) and proteome (expressed protein set from genome). This is because post-transcriptional mechanisms such as miRNA-mediated regulation impact gene expression by degrading their target mRNAs or/and inhibiting their further translation [58]. Additionally, alternative splicing of precursor mRNA [59] and post-translational modifications [60] result in several protein isoforms. The potential role of epigenetic mechanisms in endometriosis should be further investigated, since it may provide an insight into the molecular basis of altered expression. Wang et al. [27] reported an association between the altered expression levels of AC002454.1 antisense lncRNA and target *CDK6* mRNA in endometriosis. In addition, combined analyses of both, mRNAs and ncRNAs by Zhou et al. [29] and Cui et al. [38], reported an association of differentially expressed sncRNAs and lncRNAs with their putative mRNA targets. Epigenomics studies by Naqvi et al. [49] and Houshdaran et al. [50] also provided molecular explanation for observed difference in gene expression levels due to the associated aberrant DNA methylation status.

### Functional enrichment analysis by synthesised data

Functional enrichment analysis using genes sorted according to the phases of the menstrual cycle identified GO terms and KEGG pathways, potentially related with pathogenesis of endometriosis as well with affected physiological processes required for normal endometrial function and receptivity. Among top significantly enriched pathways, *oestrogen signalling pathway* was associated with P-phase. This is in correlation with Makieva et al. [14] who reviewed the important contribution of the oestradiol to the downstream pathways which enhance mitotic activity causing the thickening of the functional layer in P-phase of normo-ovulatory women [14]. Further, *negative regulation of growth* and *G1/S transition of the mitotic cell cycle* associated with the ES-phase and *MAPK* (mitogen activated protein kinase) *signalling pathway* associated by the S-phase gene set could indicate endometrial dysfunction in endometriosis. This is in accordance with the published studies [61, 62]. Velarde et al. demonstrated increased MAPK and ERK kinase 1/2 signalling cascade that inhibited cAMP-dependent cell cycle regulation in endometrial stromal fibroblasts from women with endometriosis which was further associated with potential persistence of endometrial cell proliferation from P- to S-phase [61]. Similarly, Yotova et al. have shown association of higher Ras/B-Raf/MAPK signalling activity with increased proliferation and migration rates in primary eutopic endometrial stromal cells of patients with endometriosis [62].

*Extracellular matrix organization* (ECM), *ECM-receptor interaction*, and *focal adhesion* pathways that were found to be associated with the S-phase gene list could also be related with pathophysiological mechanisms in endometriosis. In the literature, increased levels of metalloproteinases with a role in rearrangement of the ECM [63] and up-regulated levels of combined adhesion molecule ITGAV/ITGB3 integrin [64] were associated with greater invasiveness and susceptibility of sloughed menstrual cells being implanted at ectopic sites in endometriosis. In the present study, the *pathways in cancer* was associated with S-, MS-, and LS-phases. Although not statistically significant, the *MicroRNAs in cancer* was enriched with P-phase sub-group of genes. Sapalidies et al. reviewed genetic and epigenetic interactions that may contribute to the rare event of malignant transformation of the endometriosis lesions [65]. In the present analysis, also the *PI3K-Akt signalling pathway* was found to be associated with the S-phase group of genes. This is in accordance with the study by Kim et al. [66] who found that increased activation of AKT pathway in *Pten*^*f/+*^ and *PR*^*cre/+*^*Pten*^*f/+*^ mice with autologous implantation of human endometrial tissue promoted development of ectopic lesions. Identified *antigen processing and presentation* pathway associated with the S-phase in the present study may indicate an association of endometriosis with another pathogenetic mechanisms. Matarese et al. [67] reviewed endometriosis as a chronic inflammatory disease where the immune system induces autoimmunity which favours endometrioitic lesions formation.

In the present study, *cell-cell adhesion* pathway enriched by S-phase gene list may characterise affected endometrial receptivity in endometriosis. Khorram and Lessey [68] associated decreased expression levels of ITGA5/ITGB3 integrin during the WOI in endometriosis women with an unfavourable environment for embryo implantation. Furthermore, *endothelial cell chemotaxis*, *inflammatory response*, *monocyte chemotaxis*, and *lymphocyte chemotaxis* pathways associated with the MS-phase may also characterise affected receptivity. This is in accordance with the study by Lee et al. [69] where the role of chemokines and cytokines in recruitment of innate and adaptive immune cells for successful embryo implantation was reviewed.

Some pathways overlapped across different phases of the menstrual cycle. A total of 33 GO-BP and KEGG pathway terms (Table 4) were found to be associated in different phase-specific gene lists. For example, *oestrogen signalling pathway* was associated with P- and S-phases, *negative regulation of apoptotic process* was associated with P-, S-, ES-, and MS-phases, *cell-cell signalling* was associated with P- and MS-phases, and *female pregnancy* was associated with ES- and MS-phases. These pathways could indicate stronger potential for pathway-based identification of biomarkers associated with endometrial function in endometriosis.

### The heterogeneity of retrieved studies

Studies that were included in the present integrative analysis exhibit heterogeneity at various levels, i.e. study design, recruitment criteria for participating women, sample size, procedures of endometrial tissue processing, analysed omics level, platforms for genome-wide profiling, and data presentation. Different techniques for endometrial tissue sampling, preservation, and extraction protocols were used. Also, the number of used endometrial samples and applied platforms for genome-wide profiling varies across the studies. Studies had heterogeneous enrolment criteria for participating women, i.e. different types and stages of endometriosis often with coexisted uterus/pelvic pathologies, different gynaecological conditions of control women as well as symptoms of chronic pelvic pain and/or infertility. In addition, indications for endometrium tissue sample collection were heterogeneous, including endometrial biopsy, laparoscopy, hysteroscopy, or hysterectomy.

In the present GSEA, all extracted genes across 21 studies were included, regardless of the type and stage of endometriosis, coexisting uterine/pelvic pathologies and symptoms of chronic pelvic pain and/or infertility. Therefore, identified enriched terms should be interpreted with caution. It was suggested by Painter et al. [70] that severe endometriosis may have different genetic origin compared with mild endometriosis, because most significant single nucleotide polymorphisms (SNPs) were associated with stage III/IV endometriosis in genome-wide association study (GWAS). In addition, transcriptomics studies [37, 71] distinguished endometrial expression signature between mild and severe endometriosis. Some studies recruited women with endometriosis complicated with additional uterine/pelvic pathologies and/or infertility in case group [25, 37, 41, 47]. It has been reported [72, 73] that non-endometriosis gynaecological pathologies and infertility impact endometrial expression patterns as well. Hever et al. [72] distinguished endometrial transcriptome signatures according to the presence of adenomyosis and fibroids, while Koot et al. [73] observed unique endometrium gene expression signature during the MS-phase in women with recurrent implantation failure (RIF) and associated endometrium dysfunction as one of the determining factors of infertility in reproductive technique (ART) treatments. Burney et al. [40] segregated endometriosis cases and control women with leiomyomas on the basis of endometrial miRNAs expression patterns when applied unsupervised hierarchical clustering. However, one endometriosis case was clustered together with controls since this patient had coexisting leiomyoma [40].

Some studies recruited women with leiomyomas, adenomyosis, uterine adhesions, menorrhagia, dysmenorrhoea, chronic pelvic pain, and/or infertility in the control group, which could limit identification of loci specific for endometriosis [25, 37, 45, 47, 41]. Tamaresis et al. demonstrated that different uterine/pelvic pathologies leave their own fingerprints in the endometrial transcriptome signature when comparing four different groups of women, i.e. mild-, severe endometriosis, healthy women, and women with non-endometriosis uterine/pelvic pathologies, including leiomyomas, adenomyosis, benign ovarian cysts, and endometrial polyps [37].

The catalogue developed in our study including assembled genetic data (Supplementary file S1) with characteristics of corresponding extracted studies (Table 1) now enables researchers to perform additional downstream bioinformatics analyses according to different sorting criteria (experimental characteristics and clinical data). For example, data sorting can be performed according to stage or type of endometriosis or coexisting gynaecological conditions.

### Guidelines for study design harmonisation

World Endometriosis Research Foundation (WERF) Endometriosis Phenome and Biobanking Harmonisation Project (EPHect) tends to overcome study design variabilities in endometriosis research. Harmonisation of standard operating procedures (SOPs) for sampling, processing, and storaging of endometrium tissue biopsies from participating women would reduce biases and measurement errors, providing detailed surgical characterisation, including determination of the menstrual phase at the time of eutopic endometrium sample collection from women with and without endometriosis, surgical and clinical phenotypic presentation of lesions [20]. In addition, documented nonsurgical aspects, including information on chronic pelvic pain, subfertility, reproductive history, menstrual history and hormone therapies used, medical and surgical history, and personal information would improve comparison across studies and enable large-scale collaborative research [74]. While WERF EPHect provides directions for harmonised workflow across studies to identify reliable loci specific for sub-types of patients with endometriosis, it is recommended for reporting to use standardised HGNC nomenclature for gene symbols and names in publications which will facilitate data integration across studies and data exchange among researchers.

### Limitations of the study

Besides contributing to the research field, the present study also has some limitations: (1) The gene catalogue was constructed from the top differentially expressed transcripts and proteins measured by high-throughput methodologies; thus, most of them were not further validated, and may therefore suffer from biases that could impact GSEA. (2) An unequal distribution of studies performed across omics levels, higher number of transcriptomics studies in comparison with epigenomics and proteomics studies. (3) An unequal distribution of studies performed across phases of the menstrual cycle is observed. For example, low ncRNomics types of studies performed through the S-phase consequently provided no enriched pathways associated with miRNA categories in specific sub-phases of S-phase. (4) The phase determination of the menstrual cycle can be subjective, which may lead to incorrect sorting into phase-specific gene lists and influence the GSEA. Endometrial dating from extracted publications was mainly determined by histological examination of an endometrial biopsy or was categorised based on self-reported date of last menstrual period and cycle day at the time of sampling. In addition, some publications provided stage of the menstrual cycle with general S-phase, while others provided specific sub-phases of S-phase (ES-, MS-, or LS-phase). (5) Variability in indications for endometrial tissue sample collection in participating women often complicated with non-endometriosis uterine/pelvic pathologies and/or infertility may impact molecular patterns in endometriosis cases. (6) Some of the articles which do not include a term “genome-wide study” in the title or keywords might have been missed from our literature screening and should be included in the next update study.

## Conclusions

This study presents the first multi-omics data synthesis of reported altered molecular patterns associated with eutopic endometrium in endometriosis. Editing of heterogeneous nomenclature of reported genetic information resulted in the gene catalogue, which was further sorted according to the phases of the menstrual cycle. Functional enrichment analysis was applied to study the role of obtained genes in eutopic endometrium of endometriosis. The findings present a source of stronger candidate genes and pathways for further experiments in endometriosis. It is expected that current gene catalogue of endometriosis will expand by future studies. All reported data from additional omics levels (e.g. interactomics, microbiomics) should be captured to deepen insight into endometrial organisation in endometriosis. Identified pathophysiological and physiological mechanisms in eutopic endometrium of endometriosis could contribute to better diagnosis and treatment of women with endometriosis, and could increase the chances of successful pregnancy in infertile women seeking ART treatments.

## Electronic supplementary material

ESM 1 (DOCX 158 kb).

ESM 2 (DOCX 32 kb).

ESM 3 (DOCX 59 kb).

ESM 4 (XLSX 114 kb).

ESM 5 (DOCX 141 kb).

## Abbreviations

ASRM: American Society for Reproductive Medicine
GSEA: Gene set enrichment analysis
HGNC: HUGO Gene Nomenclature Committee
NCBI: National Centre for Biotechnology Information
DAVID: Database for Annotation, Visualization and Integrated Discovery
KEGG: Kyoto Encyclopedia of Genes and Genomes
GO: Gene Ontology
mRNA: Messenger RNA
lncRNA: Long non-coding RNA
sncRNA: Small non-coding RNA
snoRNA: Small nucleolar RNA
miRNA: Micro RNA
M: The menstrual phase of the menstrual cycle
P: The proliferative phase of the menstrual cycle
S: The secretory phase of the menstrual cycle
ES: The early-secretory phase of the menstrual cycle
MS: The mid-secretory phase of the menstrual cycle
LS: The late-secretory phase of the menstrual cycle
WOI: Window of implantation
N/S: Not specified
ART: Assisted reproductive techniques
