# Supplementary material for: Molecular signature of eutopic endometrium in endometriosis based on the multi-omics integrative synthesis
Source: J Assist Reprod Genet. 2020 May 30;37(7):1593–611. doi: 10.1007/s10815-020-01833-3 (PMC7376782; doi:10.1007/s10815-020-01833-3)
Supplement: Supplementary file 1 — (DOCX 158 kb). [file 10815_2020_1833_MOESM1_ESM.docx]

Supplementary file S1: The catalogue of genes associated with altered molecular patterns in eutopic endometrium of endometriosis. Extracted were highest differentially expressed transcripts and proteins from transcriptomic and proteomic studies, respectively, and differentially expressed genes associated with altered methylation level from epigenomic studies. Gene nomenclature was edited according to the HGNC database with corresponding Gene ID numbers.

| **Extraced differentially expressed transcripts/proteins with name or symbol used in source reference** | **Gene ID from NCBI** | **Approved gene name according to the HGNC nomenclature** | **Gene symbol according to the HGNC nomenclature** | **Reported dysregulation: up (↑) or down (↓) gene expression or hypo-/hypo-methlated levels in E cases** | **Reported fold change ratio when E *vs*.C** | **Reported significance** | **Phase of the cycle; other observation** | **Reference; omics level** |
| --- | --- | --- | --- | --- | --- | --- | --- | --- |
| 0-6-methylguanine-DNA methyltransferase | 4255 | 0-6-methylguanine-DNA methyltransferase | *MGMT* | (↓) (qPCR validated) (hypermeth) | 0.3 (expre.)  2.22 (methy.) | 0.015 (p values for expre.);  0.0194 (p values for methy.) | N/S* phase | [49]  Epigenomic level with expression levels of associated genes |
| dual specificity phosphatase 22 | 56940 | dual specificity phosphatase 22 | *DUSP22* | ↓ (qPCR validated) (hypermeth) | 0.3 (expre.) 2.11 (methy.) | 0.039 (expre.); 1.449E-34 (methy.) |  |  |
| cell division cycle associated 2 | 157313 | cell division cycle associated 2 | *CDCA2* | ↓ (qPCR validated) (hypermeth) | 0.06 (expre.) 1.69 (methy.) | 0.03 (expre.); 0.0207 (methy.) |  |  |
| Inhibitor of DNA binding 2 | 3298 | inhibitor of DNA binding 2 | *ID2* | ↓ (qPCR validated) (hypermeth) | 0.4 (expre.) 1.62 (methy.) | 0.004 (expre.); 3.259E-06 (methy.) |  |  |
| retinoblastoma binding protein 7 | (5931) | RB binding protein 7, chromatin remodelling factor | *(RBBP7)* | ↓ (ns* by qPCR validation)  (hypermeth) | 0.3 (expre.) 1.65 (methy.) | 0.069 (expre.);  0.007 (methy.) |  |  |
| tumor necrosis factor receptor 1B | (7133) | TNF receptor superfamily member 1B | *(TNFRSF1B)* | ↑ (ns by qPCR validation) (hypometh) | 1.3 (expre.)  -2.53 (methy.) | p >0.05 (expre.); 1.162E-09 (methy.) |  |  |
| bone morphogenetic protein receptor, type 1B | 658 | Bone morphogenetic protein receptor, type 1B | *(BMPR1B)* | ↑ (qPCR validated) (hypometh) | 2.49 (expre.)  -2.53 (methy.) | p > 0.05 (expre.); 0.0271 (methy.) |  |  |
| zinc finger protein receptor 681 | 148213 | Zinc finger protein receptor 681 | *ZNF681* | ↑ (qPCR validated) (hypometh) | 4.8 (expre.)  -1.65 (methy.) | 0.003 (expre.); 3.209E-09 (methy.) |  |  |
| immunoglobulin superfamily, member 21 | 84966 | Immunoglobulin superfamily member 21 | *IGSF21* | ↓ (qPCR validated) (hypometh) | 0.002(expre.)  -1.72 (methy.) | 0.03 (expre.);  -1.723 (methy.) |  |  |
| tumor protein 73 | (7161) | tumor protein 73 | *(TP73)* | ↓ (ns by qPCR validation) (hypometh) | 0.35 (expre.)  -1.89(methy.) | p > 0.05 (expre.); 0.0008 (methy.) |  |  |
| TATA-box-binding protein-associated factor, RNA polymerase I, subunit D | 79101 | TATA-box-binding protein associated factor, RNA polymerase I subunit D | *TAF1D* | ↑ in expression (hypometh) | 1.8 (expre.)  -0.15 (methy.) | -0.4 (Spearman rho) | (P) phase | [50]  Epigenomics level with expression levels of associated genes |
| glutathione S-transferase M5 | 2949 | glutathione S-transferase mu 5 | *GSTM5* | ↓ (hypermeth) | -1.2 (expre.) 0.17 (methy.) | -0.5 |  |  |
| TEK tyrosine kinase, endothelial | 7010 | TEK receptor tyrosine kinase | *TEK* | ↓ (hypermeth) | -1.4 (expre.) 0.16 (methy.) | -0.1 |  |  |
| glutathione S-transferase M1 isoform 1 | 2944 | glutathione S-transferase mu 1 | *GSTM1* | ↓ (hypermeth) | -1.5 (expre.) 0.25(methy.) | -0.6 |  |  |
| neutrotrimin | 50863 | neutrotrimin | *NTM* | ↓ (hypometh) | -2.0 (expre.) -0.16 (methy.) | 0.6 |  |  |
| Fas apoptotic inhibitory molecule 2 | 23017 | fas apoptotic inhibitory molecule 2 | *FAIM2* | ↓ (hypermeth) | -2.2 (expre.) 0.17 (methy.) | -0.6 |  |  |
| homeobox A5 | 3202 | homeobox A5 | *HOXA5* | ↓ (hypermethy) | -3.3 (expre.) 0.17 (methy.) | -0.8 |  |  |
| A-kinase anchor protein 12 isoform 1 | 9590 | A-kinase anchoring protein 12 | *AKAP12* | ↓ (hypermeth) | -1.9 (expre.)  0.14 (methy.) | -0.7 |  |  |
| potassium voltage-gated channel; shaker-related subfamily; beta member 1 isoform 2 | 7881 | Potassium voltage-gated channel subfamily A member regulatory beta subunit 1 | *KCNAB1* | ↑ (hypometh) | 1.9 (expre.)  -0.19 (methy.) | -0.5 | (ES) phase |  |
| frizzled 2 | 2535 | frizzled class receptor 2 | *FZD2* | ↓  (hypometh) | -4.0 (expre.) -0.15 (methy.) | 0.3 |  |  |
| glycosyltransferase 28 domain-containing 1 | 79868 | ALG13 UDP-N-acetylglucosaminyltransferase subunit | *ALG13* | ↑  (hypometh) | 4.3 (expre.)  -0.15 (methy.) | -0.8 |  |  |
| WNK lysine-deficient protein kinase 2 | 65268 | WNK lysine deficient protein kinase 2 | *WNK2* | ↓  (hypometh) | -1.7 (expre.) -0.14 (methy.) | 0.4 |  |  |
| transducer of ERBB2; 1 | 10140 | Transducer of ERBB2, 1 | *TOB1* | ↑  (hypermeth) | 6.7 (expre.) 0.15 (methy.) | 0.9) |  |  |
| hypothetical protein LOC114035 (C21orf81) | 391267 | Ankyrin repeat domain 20 family member A11, pseudogene | *ANKRD20A11P* | ↑  (hypermeth) | 1.8 (expre.) 0.15 (methy.) | 0.8 |  |  |
| 3-hydroxybutyrate dehydrogenase; type 2 | 56898 | 3-Hydroxybutyrate dehydrogenase 2 | *BDH2* | ↑  (hypermeth) | 1.6 (expre.) 0.16 (methy.) | 0.4 |  |  |
| pleckstrin | 5341 | pleckstrin | *PLEK* | ↑  (hypometh) | 1.6 (expre.)  -0.21 (methy.) | -0.8 | (MS) phase |  |
| homeobox protein A9 isoform a | 3205 | homeobox A9 | *HOXA9* | ↑  (hypometh) | 1.5 (expre.)  -0.21 (methy.) | -0.3 |  |  |
| laminin alpha 3 subunit isoform 2 | 3909 | Laminin subunit alpha 3 | *LAMA3* | ↑  (hypometh) | 3.5 (expre.)  -0.18 (methy.) | -0.8 |  |  |
| bone marrow stromal cell antigen 2 | 684 | Bone marrow stromal cell antigen 2 | *BST2* | ↑  (hypometh) | 1.3 (expre.)  -0.15 (methy.) | -0.7 |  |  |
| discs large homolog 5 | 9231 | Discs large MAGUK scaffold protein 5 | *DLG5* | ↓  (hypometh) | -1.6 (expre.) -0.14 (methy.) | 0.9 |  |  |
| Src-like adaptor | 6503 | Src like adaptor | *SLA* | ↑  (hypometh) | 2.1 (expre.);  -0.14 (methy.) | -0.8 |  |  |
| alpha 1 type XII collagen short isoform precursor | 1303 | Collagen type XII alpha 1 chain | *COL12A1* | ↑  (hypermeth) | 2.6 (expre.) 0.14 (methy.) | 0.5 |  |  |
| period 1 | 5187 | Period circadian regulator 1 | *PER1* | ↓  (hypermeth) | -2.2 (expre.) 0.15 (methy.) | -0.6 |  |  |
| CTL2 protein | 57153 | Solute carrier family 44 member 2 | *SLC44A2* | ↓  (hypermeth) | -1.4 (expre.) 0.15 (methy.) | -0.4 |  |  |
| C1q and tumor necrosis factor-related protein 6 | 114904 | C1q and TNF related 6 | *C1QTNF6* | ↑  (hypermeth) | 3.2 (expre.) 0.16 (methy.) | 0.8 |  |  |
| cyclin-dependent kinase 5; regulatory subunit 1 | 8851 | Cyclin dependent kinase 5 regulatory subunit 1 | *CDK5R1* | ↑  (hypermeth) | 1.4 (expre.) 0.18 (methy.) | 0.6 |  |  |
| endothelin receptor type B isoform 2 | 1910 | Endothelin receptor type B | *EDNRB* | ↑  (hypermeth) | 2.2 (expre.) 0.21 (methy.) | 0.4 |  |  |
| topoisomerase (DNA) Iiα, 170 kDa | 7153 | DNA topoisomerase II alpha | *TOP2A* | ↑ (qPCR validated) | 7.59 | 0.0003 (p values) | (ES) phase | [25]  Transcriptomic level |
| hypothetical protein FLJ23468 | / | / | */* | ↑ | 5.38 | 0.0011 |  |  |
| UG* = Hs.62180 (UniGene cluster ID number), gb*: AK023208.1 (GenBank accession number) | / | / (unspecific) | */* | ↑ | 5.32 | 0.0032 |  |  |
| UG = Hs.8895.0, gb: AA147933 | / | / (retired) | */* | ↑ | 5.23 | 0.0180 |  |  |
| RAB6 interacting, kinesin-like (RAB6KIFL) | 10112 | Kinesin family member 20A | *KIF20A* | ↑ | 5.19 | 0.0015 |  |  |
| apolipoprotein B mRNA editing enzyme, catalytic polypeptide-like 3B | 9582 | Apolipoprotein B mRNA editing enzyme catalytic subunit 3B | *APOBEC3B* | ↑ | 5.12 | 0.0011 |  |  |
| T-LAK cell-originated protein kinase (TOPK) | 55872 | PDZ binding kinase | *PBK* | ↑ | 5.05 | 0.0021 |  |  |
| ribonucleotide reductase M2 polypeptide | 6241 | ribonucleotide reductase regulatory subunit M2 | *RRM2* | ↑ | 4.95 | 0.0009 |  |  |
| secreted frizzled-related protein 4 | 6224 | Secreted frizzled related protein 4 | *SFRP4* | ↑ | 4.94 | 0.0431 |  |  |
| UG = Hs.62180, gb: NM_018685.1 | 54443 | Anillin actin binding protein | *ANLN* | ↑ | 4.58 | 0.0023 |  |  |
| thymidylate synthetase | 7298 | thymidylate synthetase | *TYMS* | ↑ | 4.52 | 0.0039 |  |  |
| ribonucleotide reductase M2 polypeptide | 6241 | Ribonucleotide reductase regulatory subunit M2 | *RRM2* | ↑ | 4.39 | 0.0054 |  |  |
| cell division cycle 2, G1 to S and G2 to M (CDC2) | 983 | Cyclin dependent kinase 1 | *CDK1* | ↑ | 4.33 | 0.0003 |  |  |
| synonyms: MCPH5, FLJ10517, FLJ10549; microcephaly, primary autosomal recessive 5; Homo sapiens asp (abnormal spindle)-like, microcephaly associated (Drosophila) (ASPM), mRNA. | 259266 | Abnormal spindle microtubule assembly | *ASPM* | ↑ | 4.33 | 0.0036 |  |  |
| metallothionein 1Y (MT1Y) | / | / (unspecific) | */* | ↓ | 0.09 | 0.0079 |  |  |
| gb:AF350881.1 /DB_XREF=gi:13569704 /GEN=CHAK2 /FEA=FLmRNA /CNT=1 /TID=HsAffx.900638.455 /TIER=FL /STK=0 /DEF=Homo sapiens channel kinase 2 (CHAK2) mRNA, | 140803 | Transient receptor potential cation channel subfamily M member 6 | *TRPM6* | ↓ | 0.11 | 0.0285 |  |  |
| CXCL13:chemokine ligand 13 (B-cell chemoattractant) (SCYB13) | 10563 | C-X-C motif chemokine ligand 13 | *CXCL13* | ↓ | 0.11 | 0.0018 |  |  |
| cytochrome P450, subfamily XXVIA | (1592) | cytochrome P450 family 26 subfamily A member 1 | *(CYP26A1)* | ↓ (ns by qPCR validation) | 0.12 | 0.0306 |  |  |
| secretoglobin, family 2A, member 2 | 4250 | secretoglobin family 2A member 2 | *SCGB2A2* | ↓ (qPCR validated) | 0.13 | 0.0203 |  |  |
| UG = Hs.435476, gb: AL049245.1 | / | / (retired) | */* | ↓ | 0.13 | 0.0342 |  |  |
| metallothionein 1G | 4495 | metallothionein 1G | *MT1G* | ↓ | 0.14 | 0.0054 |  |  |
| metallothionein 1X | 4501 | metallothionein 1X | *MT1X* | ↓ | 0.14 | 0.0046 |  |  |
| metallothionein 1F (functional) | 4494 | Metallothionein 1F | *MT1F* | ↓ | 0.15 | 0.0064 |  |  |
| UG = Hs.301743, gb: R00975 | / | / (retired) | */* | ↓ | 0.15 | 0.0117 |  |  |
| MT1F: Metallothionein 1F (functional) | 4494 | Metallothionein 1F | *MT1F* | ↓ | 0.15 | 0.0073 |  |  |
| cytochrome P450, family 4, subfamily B, polypeptide 1 | 1580 | cytochrome P450 family 4 subfamily B polypeptide 1 | *CYP4B1* | ↓ | 0.15 | 0.0005 |  |  |
| UG = Hs.104433, gb: AI763426 | / | / | */* | ↓ | 0.16 | 0.0004 |  |  |
| calpain 6 | 827 | Calpain 6 | *CAPN6* | ↓ | 0.16 | 0.0018 |  |  |
| metallothionein 1L | 4500 | Metallothionein 1L, pseudogene | *MT1L* | ↓ | 0.16 | 0.0050 |  |  |
| S100 calcium binding protein A8 | 6279 | S100 calcium binding protein A8 | *S100A8* | ↑ (qPCR validated) | 2.11 | 0.0156 | (MS) phase |  |
| human 18S rRNA gene, complete | / | / | */* | ↑ | 2.03 | 0.0028 |  |  |
| leukotriene B4 receptor BLTR2 (BLTR2) | 56413 | leukotriene B4 receptor 2 | *LTB4R2* | ↑ | 2.02 | 0.0005 |  |  |
| UG = Hs.221703, gb:AW291714 | / | / | */* | ↑ | 1.98 | 9.88E-06 |  |  |
| UG = Hs2.380402.1, gb: BC013935.1 | / | / (retired) | */* | ↑ | 1.92 | 4.62E-07 |  |  |
| platelet-activating factor receptor | 5724 | platelet activating factor receptor | *PTAFR* | ↑ | 1.91 | 0.00198 |  |  |
| UG = Hs.7991, gb:AA206763 | / | / | */* | ↑ | 1.91 | 3.86E-07 |  |  |
| UG = Hs.182416.0, gb: AI872284 | / | / (retired) | */* | ↑ | 1.87 | 7.84E-06 |  |  |
| granzyme A | 3001 | granzyme A | *GZMA* | ↑ | 1.87 | 0.0286 |  |  |
| UG = Hs.127680.0, gb: AW025183 | / | / Uncharacterized LOC389332 (LOC389332) | */* | ↑ | 1.84 | 2.99E-05 |  |  |
| UG = Hs.38750.0, gb: NM_024632.1 | 79685 | SAP30 like | *SAP30L* | ↑ | 1.83 | 0.0081 |  |  |
| PDK2: Pyruvate dehydrogenase kinase, isoenzyme 2 (AL134453) | / | / | */* | ↑ | 1.83 | 4.30E-05 |  |  |
| UG = Hs.289053, gb:AA521111 | / | / retired | */* | ↑ | 1.82 | 0.0013 |  |  |
| alkaline phosphatase, intestinal | 248 | alkaline phosphatase, intestinal | *ALPI* | ↑ | 1.82 | 3.06E-06 |  |  |
| tripartite motif-containing 15 | 89870 | Tripartite motif containing 15 | *TRIM15* | ↑ | 1.81 | 9.00E-07 |  |  |
| secretoglobin, family 2A, member 2 | 4250 | secretoglobin family 2A member 2 | *SCGB2A2* | ↓ (qPCR validated) | 0.30 | 0.0447 |  |  |
| /GEN=CHAK2 /FEA=FLmRNA /CNT=1 /TID=HsAffx.900638.455 /TIER=FL /STK=0 /DEF=Homo sapiens channel kinase 2 (CHAK2) mRNA, complete cds. (AF350881.1) | 140803 | Transient receptor potential cation channel subfamily M member 6 | *TRPM6* | ↓ | 0.36 | 0.0208 |  |  |
| POM (POM21 homolog, rat) and ZP3 fusion | 22932 | POM21 and ZP3 fusion | *POMZP3* | ↓ | 0.39 | 0.0020 |  |  |
| UG = Hs.201589, gb: AK026433.1 | / | / (retired) | */* | ↓ | 0.40 | 5.98E-06 |  |  |
| chromosome 10 open reading frame 10 (DEPP/C10orf10) | 11067 | DEPP1 autophagy regulator | *DEPP1* | ↓ | 0.42 | 0.0129 |  |  |
| UG = Hs.279789, gb: AI291123 | / | / (retired) | */* | ↓ | 0.43 | 0.0053 |  |  |
| UG = Hs.88651, gb: AI683621 | / | / | */* | ↓ | 0.44 | 0.0389 |  |  |
| zu43a04.s1 Soares ovary tumor NbHOT Homo sapiens cDNA clone IMAGE:740718 3' similar to contains Alu repetitive element;contains element PTR5 repetitive element mRNA sequence | / | / | */* | ↓ | 0.44 | 0.0162 |  |  |
| UG = Hs.298506, gb: AK022100.1 | / | / | */* | ↓ | 0.45 | 1.76E-06 |  |  |
| POM (POM121 homolog, rat) and ZP3 fusion | 22932 | POM21 and ZP3 fusion | *POMZP3* | ↓ | 0.45 | 0.0065 |  |  |
| UG = Hs.24139, gb: W93847 | / | / | */* | ↓ | 0.45 | 0.0061 |  |  |
| UG = Hs.250655, gb: AI110886 | / | / | */* | ↓ | 0.45 | 0.0482 |  |  |
| sema domain, immunoglobulin domain (Ig), short basic domain, secreted, (semaphorin) 3C | 10512 | Semaphorin 3C | *SEMA3C* | ↓ (qPCR validated) | 0.46 | 0.0094 |  |  |
| CRISP3: Cysteine-rich secretory protein 3 (NM_006061) | 10321 | cysteine rich secretory protein 3 | *CRISP3* | ↓ | 0.47 | 0.0031 |  |  |
| UG = Hs.32794.0, gb:AA017245 | / | / (retired) | */* | ↓ | 0.48 | 0.0027 |  |  |
| S100 calcium binding protein A8 | 6279 | S100 calcium binding protein A8 | *S100A8* | ↑ (qPCR validated) | 4.95 | 0.0111 | (P) phase |  |
| UG = Hs.65551, gb: AL355392 | 92747 | BPI fold containing family B member 1 | *BPIFB1* | ↑ | 4.74 | 0.0253 |  |  |
| putative translation initiation factor (SUI1) | 10209 | eukaryotic translation initiation factor 1 | *EIF1* | ↑ (qPCR validated) | 3.74 | 0.0136 |  |  |
| lactotransferrin | 4057 | lactotransferrin | *LTF* | ↑ (qPCR validated) | 3.51 | 0.0357 |  |  |
| human 18S rRNA gene, complete (M10098) | / | / (unspecific) | */* | ↑ | 3.20 | 0.0064 |  |  |
| UG = Hs.331099, gb: AI684439 | 10750 | GRB2 related adaptor protein | *GRAP* | ↑ | 2.90 | 0.0125 |  |  |
| cartilage linking protein 1 | 1404 | hyaluronan and proteoglycan link protein 1 | *HAPLN1* | ↑ | 2.64 | 0.0314 |  |  |
| CD163 antigen | 9332 | CD163 molecule | *CD163* | ↑ | 2.63 | 0.0038 |  |  |
| lipocalin 2 (oncogene 24p3) | 3934 | lipocalin 2 | *LCN2* | ↑ | 2.48 | 0.0124 |  |  |
| RIO kinase 3 (yeast) (SUDD) | 8780 | RIO kinase 3 | *RIOK3* | ↑ | 2.47 | 7.71E-05 |  |  |
| ceruloplasmin (ferroxidase) | 1356 | ceruloplasmin | *CP* | ↑ | 2.38 | 0.0252 |  |  |
| UG = Hs.296666, gb:AU146017 | / | / (retired) | */* | ↑ | 2.34 | 0.0216 |  |  |
| tudor repeat associator with PCTAIRE 2 (PCTAIRE2BP) | 23424 | Tudor domain containing 7 | *TDRD7* | ↑ | 2.24 | 0.0255 |  |  |
| hemoglobin, beta | 3043 | Haemoglobin subunit beta | *HBB* | ↑ | 2.20 | 0.0034 |  |  |
| S100 calcium binding protein A9 | 6280 | S100 calcium binding protein A9 | *S100A9* | ↑ | 2.20 | 0.0095 |  |  |
| UG = Hs.201589, gb: AK026433.1 | / | / (retired) | */* | ↓ | 0.38 | 9.57E-07 |  |  |
| DEAD (Asp-Glu-Ala-Asp) box polypeptide (Hs.349121) | 10521 | DEAD-box helicase 17 | *DDX17* | ↓ | 0.38 | 0.0015 |  |  |
| UG = Hs.146046, gb: AI378893 | / | / | */* | ↓ | 0.43 | 0.0235 |  |  |
| oviductal glycoprotein 1 | 5016 | oviductal glycoprotein 1 | *OVGP1* | ↓ (qPCR validated) | 0.43 | 0.0362 |  |  |
| UG = Hs.62661, gb: AW014593 | 2633 | Guanylate binding protein 1 | *GBP1* | ↓ | 0.44 | 0.0239 |  |  |
| WAP four-disulfide core domain 1 | 58189 | WAP four-disulfide core domain 1 | *WFDC1* | ↓ | 0.45 | 0.0281 |  |  |
| UG = Hs.326234, gb: AI083506 | / | / | */* | ↓ | 0.45 | 0.0013 |  |  |
| G antigen 3 (GAGE3) (NM_001473) | / | / (record withdrawn by NCBI staf) |  | ↓ | 0.45 | 4.78E-06 |  |  |
| UG = Hs.38173, gb :N71063 | / | / (retired) | */* | ↓ | 0.46 | 0.0359 |  |  |
| UG = Hs.190785, gb: BF347859 | / | / | */* | ↓ | 0.49 | 0.00012 |  |  |
| Patched homolog (Drosophila) | 5727 | Patched 1 | *PTCH1* | ↓ (qPCR validated) | 0.50 | 0.0180 |  |  |
| UG = Hs.150120, gb: BE467322 | 222256 | Cadherin related family member 3 | *CDHR3* | ↓ | 0.50 | 3.47E-07 |  |  |
| UG=Hs.190125, gb:BF196935 | / | / | */* | ↓ | 0.50 | 0.0496 |  |  |
| zinc finger protein 36, C3H type-like 2 | 678 | ZFP36 ring finger protein like 2 | *ZFP36L2* | ↓ | 0.51 | 0.0070 |  |  |
| human mRNA for fibronectin (FN precursor) | 2335 | Fibronectin 1 | *FN1* | ↓ | 0 up / 16 down | N/S*  Up- and down- regulation in E determined by 20 compariosn of E *vs*. C samples.  Fold change in expression provided only for up-regulated genes in secretory phase | (S) phase | [34]  Transcriptomics level. |
| human receptor tyrosine kinase (DTK) | 7301 | TYRO3 protein tyrosine kinase | *TYRO3* | ↓ | 0 / 15 |  |  |  |
| insulin-like growth factor binding protein-2 | 3485 | insulin like growth factor binding protein 2 | *IGFBP2* | ↓ | 1 / 15 |  |  |  |
| human transmembrane receptor precursor (PTK7) | 5754 | protein tyrosine kinase 7 (inactive) | *PTK7* | ↓ | 0 / 14 |  |  |  |
| human platelet-derived growth factor receptor alpha | 5156 | platelet derived growth factor receptor alpha | *PDGFRA* | ↓ | 0 / 13 |  |  |  |
| human 65-kilodalton phosphoprotein (p65) mRNA | / | / (unspecific) | */* | ↓ | 0 / 13 |  |  |  |
| human collagen type XVII alpha 1 (COL18A1) mRNA | 80781 | collagen type XVIII alpha 1 chain | *COL18A1* | ↓ | 0 / 13 |  |  |  |
| human subtilisin-like protein (PACE4) mRNA | 5046 | proprotein convertase subtilisin/kexin type 6 | *PCSK6* | ↓ | 1 / 13 |  |  |  |
| H. sapiens mRNA for laminin M chain (merosin) | 3908 | laminin subunit alpha 2 | *LAMA2* | ↓ | 1 / 13 |  |  |  |
| H.sapiens mRNA for ribosomal protein S26 | 6231 | ribosomal protein S26 | *RPS26* | ↓ | 1 / 13 |  |  |  |
| elastin, alt.splice 2 | 2006 | elastin | *ELN* | ↓ | 0 / 12 |  |  |  |
| human alpha-2 type V collagen gene | 1290 | collagen type V alpha 2 chain | *COL5A2* | ↓ | 0 / 12 |  |  |  |
| human mRNA for type IV collagen alpha-2 chain | 1284 | collagen type IV alpha 2 chain | *COL4A2* | ↓ | 0 / 12 |  |  |  |
| human S-adenosylhomocysteine hydrolase (AHCY) | 191 | adenosylhomocysteinase | *AHCY* | ↓ | 0 / 12 |  |  |  |
| H.sapiens p27 mRNA | 3429 | interferon alpha inducible protein 27 | *IFI27* | ↓ | 1 / 12 |  |  |  |
| cysteine-rich 61 (Cyr61) | 3491 | cellular communication network factor 1 | *CCN1* | ↑ (qPCR validated) | 5.0-fold change (18 up / 0 down) |  |  |  |
| zinc finger transcriptional regulator | / | / (unspecific) | */* | ↑ | 4.0 (16 / 0) |  |  |  |
| human AMY2B gene for alpha-amylase | 280 | amylase alpha 2B (pancreatic) | *AMY2B* | ↑ | 2.7 (16 / 0) |  |  |  |
| TR3 orphan receptor mRNA | 3164 | nuclear receptor subfamily 4 group A member 1 | *NR4A1* | ↑ | 2.2 (16 / 0) |  |  |  |
| mRNA for integrin beta 4 | 3691 | integrin subunit beta 4 | *ITGB4* | ↑ | 2.1 (15 / 0) |  |  |  |
| 3,4-catechol estrogen UDP-glucuronosyltransferase | 7364 | UDP glucuronosyltransferase family 2 member B7 | *UGT2B7* | ↑ | 2.4 (15 / 0) |  |  |  |
| H.sapiens CL 100 mRNA for protein tyrosine phosphatase | 1843 | dual specificity phosphatase 1 | *DUSP1* | ↑ | 4.8 (14 / 0) |  |  |  |
| connective tissue growth factor (CTGF) | 1490 | cellular communication network factor 2 | *CCN2* | ↑ | 1.8 (13 / 0) |  |  |  |
| H.sapiens protein tyrosine phosphatase (PAC-1) (GenBank: L11329) | 1844 | dual specificity phosphatase 2 | *DUSP2* | ↑ | 6.3 (13 / 1) |  |  |  |
| human transcription factor junB (junB) gene | 3726 | JunB proto-oncogene, AP-1 transcription factor subunit | *JUNB* | ↑ | 3.7 (13 / 1) |  |  |  |
| human clone YDD19 mRNA sequence (GenBank:U82319) | 11049 | NUS1 pseudogene 3 | *NUS1P3* | ↑ | 2.4 (13 / 1) |  |  |  |
| human protein tyrosine phosphatase PTPCAAX1 | 7803 | protein tyrosine phosphatase 4A1 | *PTP4A1* | ↑ | 2.1 (13 / 1) |  |  |  |
| mRNA for early growth response protein 1 | 1958 | early growth response 1 | *EGR1* | ↑ | 3.8 (12 / 0) |  |  |  |
| cellular oncogene c-fos | 2353 | Fos proto-oncogene, AP-1 transcription factor subunit | *FOS* | ↑ | 3.3 (12 / 0) |  |  |  |
| H.sapiens Wee1 hu gene | 7465 | WEE1 G2 checkpoint kinase | *WEE1* | ↑ | 2.0 (12 / 0) |  |  |  |
| H.sapiens PISSLRE mRNA | 8558 | cyclin dependent kinase 10 | *CDK10* | ↓ | 1 up / 26 down | N/S | (P) phase |  |
| homo sapiens zeta-crystallin/quinone reductase mRNA | 1429 | crystallin zeta | *CRYZ* | ↓ | 0 / 22 |  |  |  |
| human annexin IV (ANX4) mRNA | 307 | annexin A4 | *ANXA4* | ↓ | 0 / 22 |  |  |  |
| human mRNA for phosphatidylinositol-glycan-class C (PIG-C) (GenBank:D85418) | 5279 | phosphatidylinositol glycan anchor biosynthesis class C | *PIGC* | ↓ | 0 / 20 |  |  |  |
| human calmodulin mRNA | 801 | calmodulin 1 | *CALM1* | ↓ | 0 / 20 |  |  |  |
| H.sapiens, gene for membrane cofactor protein | 4179 | CD46 molecule | *CD46* | ↓ | 0 / 19 |  |  |  |
| human X104 mRNA, complete cds | 9414 | tight junction protein 2 | *TJP2* | ↓ | 0 / 19 |  |  |  |
| human mRNA for KIAA0037 gene | 113 | adenylate cyclase 7 | *ADCY7* | ↓ | 0 / 19 |  |  |  |
| L-arginine:glycine amidinotransferase | 2628 | glycine amidinotransferase | *GATM* | ↓ | 1 / 19 |  |  |  |
| HG3484-HT3678 protein kinase (GenBank: M59287) | / | / unspecific | */* | ↓ | 0 / 18 |  |  |  |
| human nuclear orphan receptor LXR-alpha mRNA | 10062 | nuclear receptor subfamily 1 group H member 3 | *NR1H3* | ↓ | 0 / 18 |  |  |  |
| H.sapiens mRNA for metabotropic glutamate receptor type 4 | 2914 | glutamate metabotropic receptor 4 | *GRM4* | ↓ | 0 / 18 |  |  |  |
| human alcohol dehydrogenase chi polypeptide (ADH5) gene | 128 | alcohol dehydrogenase 5 (class III), chi polypeptide | *ADH5* | ↓ | 0 / 17 |  |  |  |
| H.sapiens mRNA for lung amiloride sensitive Na+ channel protein | 6337 | sodium channel epithelial 1 alpha subunit | *SCNN1A* | ↓ | 0 / 17 |  |  |  |
| homo sapiens p33/HEH epoxide hydrolase (EPHX) mRNA | 2052 | epoxide hydrolase 1 | *EPHX1* | ↓ | 0 / 17 |  |  |  |
| human omega light chain protein 14.1 (Ig lambda chain related) gene (GenBank: M34516) | / | / | */* | ↑ | 27 up / 0 down |  |  |  |
| human Ig rearranged gamma chain mRNA, V-J-C region (GenBAnk: M63438) | / | / | */* | ↑ | 20 / 1 |  |  |  |
| human microfibril-associated glycoprotein 4 (MFAP4) mRNA | 4239 | microfibril associated protein 4 | *MFAP4* | ↑ | 17 / 0 |  |  |  |
| human mRNA for unknown product, complete cds (GenBank: D28124) | / | / | */* | ↑ | 17 / 0 |  |  |  |
| human thymosin beta-4 mRNA | 7114 | thymosin beta 4 X-linked | *TMSB4X* | ↑ | 16 / 0 |  |  |  |
| H.sapiens S100A2 gene, exon 1,2 and 3 | 6273 | S100 calcium binding protein A2 | *S100A2* | ↑ | 15 / 0 |  |  |  |
| homo sapiens gallbladder mucin MUC5B mRNA | 727897 | mucin 5B, oligomeric mucus/gel-forming | *MUC5B* | ↑ | 15 / 0 |  |  |  |
| human phorbolin I mRNA | 200315 | apolipoprotein B mRNA editing enzyme catalytic subunit 3A | *APOBEC3A* | ↑ | 15 / 0 |  |  |  |
| osteoglycin | 4969 | osteoglycin | *OGN* | ↓ | 0.53 | 0.00004 (p values) | (LS) phase | [36]  Transcriptomics level |
| protein tyrosin phosphatase, receptor type, R | 5801 | protein tyrosine phosphatase receptor type R | *PTPRR* | ↑ (qPCR validated) | 2.17 | 0.0024 |  |  |
| protocadherin 17 | 27253 | protocadherin 17 | *PCDH17* | ↑ (qPCR validated) | 2.11 | 0.0091 |  |  |
| BAI1-associated protein 2 | 10458 | BAI1 associated protein 2 | *BAIAP2* | ↑ | 1.85 | 0.0036 |  |  |
| fibronectin 1 | 2335 | fibronectin 1 | *FN1* | ↑ | 1.78 | 0.0061 |  |  |
| synuclein, gamma | 6623 | synuclein gamma | *SNCG* | ↑ | 1.78 | 0.0026 |  |  |
| carboxypeptidase E | 1363 | carboxypeptidase E | *CPE* | ↑ | 1.77 | 0.0057 |  |  |
| cytrochrome P450, Family 2, Subfamily J, polypeptide 2 | 1573 | cytochrome P450 family 2 subfamily J member 2 | *CYP2J2* | ↑ | 1.76 | 0.0051 |  |  |
| interleukin-6 signal transducer | 3572 | interleukin 6 signal transducer | *IL6ST* | ↑ (qPCR validated) | 1.75 | 0.0028 |  |  |
| KMO | 8564 | kynurenine 3-monooxygenase | *KMO* | ↑ | 13.76 | 0.0276 (p value) | (LS) phase | [27]  Transcriptomics level  (Only top 10 genes provided in the article) |
| FAM118A | 55007 | family with sequence similarity 118 member A | *FAM118A* | ↑ | 13.63 | 0.0339 |  |  |
| LRRN1 | 57633 | leucine rich repeat neuronal 1 | *LRRN1* | ↑ | 13.03 | 0.0034 |  |  |
| ITGA2B | 3674 | integrin subunit alpha 2b | *ITGA2B* | ↑ | 8.32 | 0.0001 |  |  |
| RDM1 | 201299 | RAD52 motif containing 1 | *RDM1* | ↑ | 8.32 | 0.0348 |  |  |
| BHLHE40 | 8553 | basic helix-loop-helix family member e40 | *BHLHE40* | ↑ | 8.29 | 0.0066 |  |  |
| GLI1 | 2735 | GLI family zinc finger 1 | *GLI1* | ↑ | 8.09 | 0.0117 |  |  |
| CDK6 | 1021 | cyclin dependent kinase 6 | *CDK6* | ↑ (qPCR validated) | 7.70 | 0.0107 |  |  |
| SHISA6 | 388336 | shisa family member 6 | *SHISA6* | ↑ | 7.58 | 0.0015 |  |  |
| HPSE2 | 60495 | heparanase 2 (inactive) | *HPSE2* | ↑ | 7.34 | 0.0429 |  |  |
| SCGB2A2 | 4250 | Secretoglobin family 2A member 2 | *SCGB2A2* | ↓ | 178.58 | 0.00000992 |  |  |
| DEFB1 | 1672 | defensin beta 1 | *DEFB1* | ↓ | 69.08 | 0.0006 |  |  |
| ORM1 | 5004 | orosomucoid 1 | *ORM1* | ↓ | 35.74 | 0.00003 |  |  |
| STC1 | 6781 | stanniocalcin 1 | *STC1* | ↓ | 30.31 | 0.0005 |  |  |
| HPSE | 10855 | heparanase | *HPSE* | ↓ | 20.13 | 0.0028 |  |  |
| S100P | 6286 | S100 calcium binding protein P | *S100P* | ↓ | 18.72 | 0.0015 |  |  |
| SCGB1D4 | 404552 | Secretoglobin family 1D member 4 | *SCGB1D4* | ↓ | 16.91 | 0.0453 |  |  |
| RXFP1 | 59350 | Relaxin family peptide receptor 1 | *RXFP1* | ↓ | 16.63 | 0.0137 |  |  |
| SCGB2A1 | 4246 | Secretoglobin family 2A member 1 | *SCGB2A1* | ↓ | 13.93 | 0.0412 |  |  |
| JAKMIP1 | 152789 | Jenus kinase and microtubule interacting protein 1 | *JAKMIP1* | ↓ | 13.89 | 0.0001 |  |  |
| ribosomal protein S17 | 6218 | ribosomal protein S17 | *RPS17* | ↑ | 1.60 | N/S | (P) phase  Comparison between endometriosis cases *vs.* non-endometriosis, but with other uterine/pelvic pathologies controls  (E *vs*. NE.UPP) | [37]  Transcriptomics level |
| polyglutamine binding protein 1 | 10084 | polyglutamine binding protein 1 | *PQBP1* | ↑ | 1.54 |  |  |  |
| cysteine-rich, angiogenic inducer, 61, CYR61 | 3491 | cellular communication network factor 1 | *CCN1* | ↓ | -6.33 |  |  |  |
| matrix Gla protein | 4256 | matrix Gla protein | *MGP* | ↓ | -5.64 |  |  |  |
| endothelin 1 | 1906 | endothelin 1 | *EDN1* | ↓ | -3.50 |  |  |  |
| proprotein convertase subtilisin/kexin type 5 | 5125 | proprotein convertase subtilisin/kexin type 5 | *PCSK5* | ↓ | -3.09 |  |  |  |
| actin, alpha 2, smooth muscle, aorta | 59 | actin alpha 2, smooth muscle | *ACTA2* | ↓ | -3.00 |  |  |  |
| hypothetical LOC284454 | / | / | */* | ↓ | -2.86 |  |  |  |
| ST8 alpha-N-acetyl-neuraminide alpha-2,8-sialyltransferase 2 | 8128 | ST8 alpha-N-acetyl-neuraminide alpha-2,8-sialyltransferase 2 | *ST8SIA2* | ↓ | -2.60 |  |  |  |
| nuclear factor of activated T-cells 5, tonicity-responsive | 10725 | nuclear factor of activated T cells 5 | *NFAT5* | ↓ | -2.59 |  |  |  |
| phosphoprotein associated with glycosphingolipid microdomains 1 | 55824 | phosphoprotein membrane anchor with glycosphingolipid microdomains 1 | *PAG1* | ↓ | -2.57 |  |  |  |
| AF4/FMR2 family, member 4 | 27125 | AF4/FMR2 family member 4 | *AFF4* | ↓ | -2.44 |  |  |  |
| Hypothetical LOC728264 | / | / | */* | ↓ | -2.38 |  |  |  |
| LON peptidase N-terminal domain and ring finger 2 | 164832 | LON peptidase N-terminal domain and ring finger 2 | *LONRF2* | ↓ | -2.27 |  |  |  |
| olfactomedin-like 1 | 283298 | olfactomedin like 1 | *OLFML1* | ↓ | -2.21 |  |  |  |
| myeloid/lymphoid or mixed-lineage leukemia (trithorax homolog, Drosophila); translocated to, 3 | 4300 | MLLT3 super elongation complex subunit | *MLLT3* | ↓ | -2.18 |  |  |  |
| protein phosphatase 1, regulatory (inhibitor) subunit 12B | 4660 | protein phosphatase 1 regulatory subunit 12B | *PPP1R12B* | ↓ | -2.13 |  |  |  |
| tropomyosin 1 (alpha) | 7168 | tropomyosin 1 | *TPM1* | ↓ | -2.08 |  |  |  |
| zinc finger protein 827 | 152485 | zinc finger protein 827 | *ZNF827* | ↓ | -3.25 | N/S | (ES) phase  E*vs*.NE.UPP |  |
| ubiquitin specific peptidase 36 | 57602 | ubiquitin specific peptidase 36 | *USP36* | ↓ | -2.97 |  |  |  |
| non-protein coding RNA 152 | 112597 | cytoskeleton regulator RNA | *CYTOR* | ↓ | -2.95 |  |  |  |
| PTK2 protein tyrosine kinase 2 | 5747 | protein tyrosine kinase 2 | *PTK2* | ↓ | -2.82 |  |  |  |
| hypothetical LOC100126784 | / | / | */* | ↓ | -2.64 |  |  |  |
| U2-associated SR140 protein, SR140 | 23350 | U2 snRNP associated SURP domain containing | *U2SURP* | ↓ | -2.46 |  |  |  |
| parvin, alpha | 55742 | parvin alpha | *PARVA* | ↓ | -2.28 |  |  |  |
| negative regulator of ubiquitin-like proteins 1 | 51667 | negative regulator of ubiquitin like proteins 1 | *NUB1* | ↓ | -2.25 |  |  |  |
| transcription factor 12 | 6938 | transcription factor 12 | *TCF12* | ↓ | -2.19 |  |  |  |
| methylcrotonoyl-CoA carboxylase 2 (beta) | 64087 | methylcrotonoyl-CoA carboxylase 2 | *MCCC2* | ↓ | -2.15 |  |  |  |
| protein phosphatase 1, regulatory (inhibitor) subunit 10 | 5514 | protein phosphatase 1 regulatory subunit 10 | *PPP1R10* | ↓ | -1.20 |  |  |  |
| SEC24 family, member A (S. cerevisiae) | 10802 | SEC24 homolog A, COPII coat complex component | *SEC24A* | ↓ | -1.98 |  |  |  |
| spectrin, alpha, non-erythrocytic 1 (alpha-fodrin) | 6709 | spectrin alpha, non-erythrocytic 1 | *SPTAN1* | ↓ | -1.94 |  |  |  |
| sodium channel, voltage-gated, type XI, alpha subunit | 11280 | sodium voltage-gated channel alpha subunit 11 | *SCN11A* | ↓ | -1.92 |  |  |  |
| mediator complex subunit 12 | 9968 | mediator complex subunit 12 | *MED12* | ↓ | -1.92 |  |  |  |
| transmembrane emp24 protein transport domain containing 4 | 222068 | transmembrane p24 trafficking protein 4 | *TMED4* | ↑ | 3.29 |  |  |  |
| mitochondrial ribosomal protein S18C | 51023 | mitochondrial ribosomal protein S18C | *MRPS18C* | ↑ | 2.36 |  |  |  |
| transmembrane protein 50A | 23585 | transmembrane protein 50A | *TMEM50A* | ↑ | 2.01 |  |  |  |
| ring finger protein 7 | 9616 | ring finger protein 7 | *RNF7* | ↑ | 1.90 |  |  |  |
| Sin3A-associated protein, 18kDa | 10284 | Sin3A associated protein 18 | *SAP18* | ↑ | 1.86 |  |  |  |
| DnaJ (Hsp40) homolog, subfamily C, member 19 | 131118 | DnaJ heat shock protein family (Hsp40) member C19 | *DNAJC19* | ↑ | 1.86 |  |  |  |
| N(alpha)-acetyltransferase 38, NatC auxiliary subunit | 84316 | N(alpha)-acetyltransferase 38, NatC auxiliary subunit | *NAA38* | ↑ | 1.85 |  |  |  |
| mitochondrial ribosomal protein L51 | 51258 | mitochondrial ribosomal protein L51 | *MRPL51* | ↑ | 1.72 |  |  |  |
| serine palmitoyltransferase, long chain base subunit 1 | 10558 | serine palmitoyltransferase long chain base subunit 1 | *SPTLC1* | ↑ | 1.72 |  |  |  |
| multiple C2 domains, transmembrane 2 | 55784 | multiple C2 and transmembrane domain containing 2 | *MCTP2* | ↑ | 1.66 |  |  |  |
| NADH dehydrogenase (ubiquinone) 1 alpha subcomplex, 1, 7.5kDa | 4694 | NADH:ubiquinone oxidoreductase subunit A1 | *NDUFA1* | ↑ | 1.63 |  |  |  |
| stromal cell-derived factor 2 | 6388 | stromal cell derived factor 2 | *SDF2* | ↑ | 1.60 |  |  |  |
| solute carrier family 1 (neuronal/epithelial high affinity glutamate transporter, system Xag), member 1 | 6505 | solute carrier family 1 member 1 | *SLC1A1* | ↑ | 5.61 | N/S | (MS) phase  E *vs*. NE.UPP |  |
| cytochrome P450, family 3, subfamily A, polypeptide 5 | 1577 | cytochrome P450 family 3 subfamily A member 5 | *CYP3A5* | ↑ | 4.88 |  |  |  |
| hypothetical protein LOC201477 | / | / | */* | ↑ | 3.39 |  |  |  |
| eukaryotic translation initiation factor 1 | 10209 | eukaryotic translation initiation factor 1 | *EIF1* | ↑ | 3.04 |  |  |  |
| paired box 8 | 7849 | paired box 8 | *PAX8* | ↑ | 3.01 |  |  |  |
| ATP-binding cassette, sub-family C (CFTR/MRP), member 3 | 8714 | ATP binding cassette subfamily C member 3 | *ABCC3* | ↑ | 2.94 |  |  |  |
| G protein-coupled receptor 110, GPR110 | 266977 | adhesion G protein-coupled receptor F1 | *ADGRF1* | ↑ | 2.66 |  |  |  |
| aminoacyl tRNA synthetase complex-interacting multifunctional protein 1 | 9255 | aminoacyl tRNA synthetase complex interacting multifunctional protein 1 | *AIMP1* | ↑ | 2.58 |  |  |  |
| vascular endothelial growth factor A | 7422 | vascular endothelial growth factor A | *VEGFA* | ↑ | 2.56 |  |  |  |
| solute carrier family 15, member 4 | 121260 | solute carrier family 15 member 4 | *SLC15A4* | ↑ | 2.44 |  |  |  |
| guanylate cyclase 1, soluble, beta 3, GUCY1B3 | 2983 | guanylate cyclase 1 soluble subunit beta 1 | *GUCY1B1* | ↑ | 2.36 |  |  |  |
| hypothetical LOC388692 | / | / | */* | ↑ | 1.89 |  |  |  |
| Y box binding protein 1 | 4904 | Y-box binding protein 1 | *YBX1* | ↑ | 1.76 |  |  |  |
| Y box binding protein 1 pseudogene 2 | 646531 | Y-box binding protein 1 pseudogene 2 | *YBX1P2* | ↑ | 1.76 |  |  |  |
| met proto-oncogene (hepatocyte growth factor receptor) | 4233 | MET proto-oncogene, receptor tyrosine kinase | *MET* | ↑ | 1.75 |  |  |  |
| dystonin | 667 | dystonin | *DST* | ↓ | -3.82 |  |  |  |
| ring finger protein 150 | 57484 | ring finger protein 150 | *RNF150* | ↓ | -3.62 |  |  |  |
| proprotein convertase subtilisin/kexin type 5 | 5125 | proprotein convertase subtilisin/kexin type 5 | *PCSK5* | ↓ | -3.34 |  |  |  |
| HLA-B associated transcript 2-like 2, BAT2L2 | 23215 | proline rich coiled-coil 2C | *PRRC2C* | ↓ | -3.26 |  |  |  |
| SON DNA binding protein | 6651 | SON DNA binding protein | *SON* | ↓ | -3.06 |  |  |  |
| nuclear factor of activated T-cells 5, tonicity-responsive | 10725 | nuclear factor of activated T cells 5 | *NFAT5* | ↓ | -3.01 |  |  |  |
| leucine zipper protein 1 | 7798 | leucine zipper protein 1 | *LUZP1* | ↓ | -2.86 |  |  |  |
| LON peptidase N-terminal domain and ring finger 2 | 164832 | LON peptidase N-terminal domain and ring finger 2 | *LONRF2* | ↓ | -2.61 |  |  |  |
| thyroid hormone receptor associated protein 3 | 9967 | thyroid hormone receptor associated protein 3 | *THRAP3* | ↓ | -2.53 |  |  |  |
| mucin 7, secreted | 4589 | mucin 7, secreted | *MUC7* | ↓ | -2.50 |  |  |  |
| AF4/FMR2 family, member 4 | 27125 | AF4/FMR2 family member 4 | *AFF4* | ↓ | -2.49 |  |  |  |
| RAP1 interacting factor homolog (yeast) | 55183 | replication timing regulatory factor 1 | *RIF1* | ↓ | -2.47 |  |  |  |
| microtubule-associated protein 4 | 4134 | microtubule associated protein 4 | *MAP4* | ↓ | -2.47 |  |  |  |
| pleckstrin homology domain containing, family A (phosphoinositide binding specific) member 2 | 59339 | pleckstrin homology domain containing A2 | *PLEKHA2* | ↓ | -2.46 |  |  |  |
| LIM domain containing preferred translocation partner in lipoma | 4026 | LIM domain containing preferred translocation partner in lipoma | *LPP* | ↓ | -2.46 |  |  |  |
| FBJ murine osteosarcoma viral oncogene homolog B | 2354 | FosB proto-oncogene, AP-1 transcription factor subunit | *FOSB* | ↑ (qPCR validated) | 34.11 | N/S | (P) phase  Comparison between endometriosis cases *vs*. non-endometriosis and free of other uterine/pelvic pathologies controls (healthy)  (E *vs*. NE.NUPP) |  |
| FBJ murine osteosarcoma viral oncogene homolog | 2353 | Fos proto-oncogene, AP-1 transcription factor subunit | *FOS* | ↑ (qPCR validated) | 15.21 |  |  |  |
| early growth response 1 | 1958 | early growth response 1 | *EGR1* | ↑ | 15.21 |  |  |  |
| jun B proto-oncogene | 3726 | JunB proto-oncogene, AP-1 transcription factor subunit | *JUNB* | ↑ | 13.14 |  |  |  |
| metastasis suppressor 1-like | 92154 | MTSS I-BAR domain containing 2 | *MTSS2* | ↑ | 11.39 |  |  |  |
| zinc finger protein 36, C3H type, homolog (mouse) | 7538 | ZFP36 ring finger protein | *ZFP36* | ↑ | 10.12 |  |  |  |
| splicing factor 3a, subunit 2, 66kDa | 8175 | splicing factor 3a subunit 2 | *SF3A2* | ↑ | 9.41 |  |  |  |
| glucose 6 phosphatase, catalytic, 3 | 92579 | glucose-6-phosphatase catalytic subunit 3 | *G6PC3* | ↑ | 9.39 |  |  |  |
| zinc finger protein 580 | 51157 | zinc finger protein 580 | *ZNF580* | ↑ | 8.99 |  |  |  |
| FXYD domain containing ion transport regulator 1 | 5348 | FXYD domain containing ion transport regulator 1 | *FXYD1* | ↑ | 8.89 |  |  |  |
| SRY (sex determining region Y)-box 13 | 9580 | SRY-box 13 | *SOX13* | ↑ | 8.81 |  |  |  |
| epoxide hydrolase 1, microsomal (xenobiotic) | 2052 | epoxide hydrolase 1 | *EPHX1* | ↑ | 8.58 |  |  |  |
| cAMP responsive element binding protein 3-like 1 | 90993 | cAMP responsive element binding protein 3 like 1 | *CREB3L1* | ↑ | 8.35 |  |  |  |
| ribonucleoprotein, PTB-binding 1 | 125950 | ribonucleoprotein, PTB binding 1 | *RAVER1* | ↑ | 8.29 |  |  |  |
| mucin 5B, oligomeric mucus/gel-forming | 727897 | mucin 5B, oligomeric mucus/gel-forming | *MUC5B* | ↑ (qPCR validated) | 8.19 |  |  |  |
| deiodinase, iodothyronine, type II | 1734 | iodothyronine deiodinase 2 | *DIO2* | ↓ (qPCR validated) | -19.34 |  |  |  |
| palladin, cytoskeletal associated protein | 23022 | palladin, cytoskeletal associated protein | *PALLD* | ↓ | -14.31 |  |  |  |
| heat shock protein 90kDa alpha (cytosolic), class B member 1 | 3326 | heat shock protein 90 alpha family class B member 1 | *HSP90AB1* | ↓ | -11.89 |  |  |  |
| structural maintenance of chromosomes 3 | 9126 | structural maintenance of chromosomes 3 | *SMC3* | ↓ | -11.68 |  |  |  |
| plakophilin 4 | 8502 | plakophilin 4 | *PKP4* | ↓ | -10.63 |  |  |  |
| hypothetical LOC647979 | / | / | */* | ↓ | -10.03 |  |  |  |
| zinc finger and BTB domain containing 38 | 253461 | zinc finger and BTB domain containing 38 | *ZBTB38* | ↓ | -9.60 |  |  |  |
| versican | 1462 | versican | *VCAN* | ↓ | -9.33 |  |  |  |
| myosin, heavy chain 10, non-muscle | 4628 | myosin heavy chain 10 | *MYH10* | ↓ | -9.25 |  |  |  |
| family with sequence similarity 76, member B | 143684 | family with sequence similarity 76 member B | *FAM76B* | ↓ | -9.18 |  |  |  |
| topoisomerase (DNA) II alpha 170kDa | 7153 | DNA topoisomerase II alpha | *TOP2A* | ↓ | -9.09 |  |  |  |
| versican | 1462 | versican | *VCAN* | ↓ | -9.04 |  |  |  |
| fibrillin 1 | 2200 | fibrillin 1 | *FBN1* | ↓ | -9.04 |  |  |  |
| dihydrofolate reductase | 1719 | dihydrofolate reductase | *DHFR* | ↓ | -9.00 |  |  |  |
| prenylcysteine oxidase 1 | 51449 | prenylcysteine oxidase 1 | *PCYOX1* | ↓ | -8.89 |  |  |  |
| early growth response 1 | 1958 | early growth response 1 | *EGR1* | ↑ | 31.36 | N/S | (ES) phase  E*vs*.NE.NUPP |  |
| FBJ murine osteosarcoma viral oncogene homolog | 2353 | Fos proto-oncogene, AP-1 transcription factor subunit | *FOS* | ↑ | 29.18 |  |  |  |
| cystatin SN | 1469 | cystatin SN | *CST1* | ↑ | 21.81 |  |  |  |
| FBJ murine osteosarcoma viral oncogene homolog B | 2354 | FosB proto-oncogene, AP-1 transcription factor subunit | *FOSB* | ↑ | 10.26 |  |  |  |
| epoxide hydrolase 1, microsomal (xenobiotic) | 2052 | epoxide hydrolase 1 | *EPHX1* | ↑ | 10.01 |  |  |  |
| hypothetical LOC57235 (KIAA0485) | / | / | */* | ↑ | 9.48 |  |  |  |
| cystatin S | 1472 | cystatin S | *CST4* | ↑ | 8.93 |  |  |  |
| CASP8 and FADD-like apoptosis regulator | 8837 | CASP8 and FADD like apoptosis regulator | *CFLAR* | ↑ | 8.30 |  |  |  |
| metastasis associated lung adenocarcinoma transcript 1 (non-protein coding) | 378938 | metastasis associated lung adenocarcinoma transcript 1 | *MALAT1* | ↑ | 7.88 |  |  |  |
| phosphatase and tensin homolog | 5728 | phosphatase and tensin homolog | *PTEN* | ↑ | 7.72 |  |  |  |
| apolipoprotein D | 347 | apolipoprotein D | *APOD* | ↑ | 7.26 |  |  |  |
| elastin | 2006 | elastin | *ELN* | ↑ | 7.19 |  |  |  |
| nicotinamide phosphoribosyltransferase | 10135 | nicotinamide phosphoribosyltransferase | *NAMPT* | ↑ | 7.19 |  |  |  |
| uncoupling protein 2 (mitochondrial, proton carrier) | 7351 | uncoupling protein 2 | *UCP2* | ↑ | 7.13 |  |  |  |
| spectrin, alpha, non-erythrocytic 1 (alpha-fodrin) | 6709 | spectrin alpha, non-erythrocytic 1 | *SPTAN1* | ↑ | 7.04 |  |  |  |
| catenin (cadherin-associated protein), beta 1, 88kDa | 1499 | catenin beta 1 | *CTNNB1* | ↓ | -9.00 |  |  |  |
| oviductal glycoprotein 1, 120kDa | 5016 | oviductal glycoprotein 1 | *OVGP1* | ↓ | -7.34 |  |  |  |
| sentan, cilia apical structure protein | 132203 | sentan, cilia apical structure protein | *SNTN* | ↓ | -7.11 |  |  |  |
| olfactomedin 4 | 10562 | olfactomedin 4 | *OLFM4* | ↓ | -6.88 |  |  |  |
| N-myristoyltransferase 2 | 9397 | N-myristoyltransferase 2 | *NMT2* | ↓ | -6.49 |  |  |  |
| 3-ketodihydrosphingosine reductase | 2531 | 3-ketodihydrosphingosine reductase | *KDSR* | ↓ | -6.38 |  |  |  |
| serine palmitoyltransferase, long chain base subunit 2 | 9517 | serine palmitoyltransferase long chain base subunit 2 | *SPTLC2* | ↓ | -6.17 |  |  |  |
| carboxypeptidase M | 1368 | carboxypeptidase M | *CPM* | ↓ | -6.09 |  |  |  |
| family with sequence similarity 76, member B | 143684 | family with sequence similarity 76 member B | *FAM76B* | ↓ | -5.83 |  |  |  |
| chromosome 13 open reading frame 30, C13orf30 | 144809 | family with sequence similarity 216 member B | *FAM216B* | ↓ | -5.75 |  |  |  |
| kelch repeat and BTB (POZ) domain containing 6 | 89890 | kelch repeat and BTB domain containing 6 | *KBTBD6* | ↓ | -5.64 |  |  |  |
| SET nuclear oncogene | 6418 | SET nuclear proto-oncogene | *SET* | ↓ | -5.60 |  |  |  |
| NUF2, NDC80 kinetochore complex component, homolog (S. cerevisiae) | 83540 | NUF2 component of NDC80 kinetochore complex | *NUF2* | ↓ | -5.57 |  |  |  |
| family with sequence similarity 175, member A; FAM175A | 84142 | abraxas 1, BRCA1 A complex subunit | *ABRAXAS1* | ↓ | -5.57 |  |  |  |
| anillin, actin binding protein | 54443 | anillin actin binding protein | *ANLN* | ↓ | -5.55 |  |  |  |
| FBJ murine osteosarcoma viral oncogene homolog | 2353 | Fos proto-oncogene, AP-1 transcription factor subunit | *FOS* | ↑ | 33.65 | N/S | (MS) phase E *vs.* NE.NUPP |  |
| FBJ murine osteosarcoma viral oncogene homolog B | 2354 | FosB proto-oncogene, AP-1 transcription factor subunit | *FOSB* | ↑ | 17.93 |  |  |  |
| early growth response 1 | 1958 | early growth response 1 | *EGR1* | ↑ | 15.90 |  |  |  |
| amiloride binding protein 1 (amine oxidase (copper-containing)); ABP1 | 26 | amine oxidase copper containing 1 | *AOC1* | ↑ | 6.65 |  |  |  |
| cathepsin W | 1521 | cathepsin W | *CTSW* | ↑ | 6.58 |  |  |  |
| chemokine (C-C motif) ligand 3 | 6348 | C-C motif chemokine ligand 3 | *CCL3* | ↑ | 6.55 |  |  |  |
| chemokine (C-C motif) ligand 3-like 1 | 6349 | C-C motif chemokine ligand 3 like 1 | *CCL3L1* | ↑ | 6.55 |  |  |  |
| chemokine (C-C motif) ligand 3-like 3 | 414062 | C-C motif chemokine ligand 3 like 3 | *CCL3L3* | ↑ | 6.55 |  |  |  |
| cysteine-rich, angiogenic inducer, 61 | 3491 | cellular communication network factor 1 | *CCN1* | ↑ | 6.32 |  |  |  |
| Duffy blood group, chemokine receptor; DARC | 2532 | atypical chemokine receptor 1 (Duffy blood group) | *ACKR1* | ↑ | 6.08 |  |  |  |
| complement component 1, q subcomponent, A chain | 712 | complement C1q A chain | *C1QA* | ↑ | 5.92 |  |  |  |
| cystatin F (leukocystatin) | 8530 | cystatin F | *CST7* | ↑ | 5.64 |  |  |  |
| zinc finger protein 36, C3H type, homolog (mouse) | 7538 | ZFP36 ring finger protein | *ZFP36* | ↑ | 5.48 |  |  |  |
| jun B proto-oncogene | 3726 | JunB proto-oncogene, AP-1 transcription factor subunit | *JUNB* | ↑ | 5.47 |  |  |  |
| cytidine deaminase | 978 | cytidine deaminase | *CDA* | ↑ | 5.42 |  |  |  |
| chromosome 1 open reading frame 63, C1orf63 | 57035 | arginine and serine rich protein 1 | *RSRP1* | ↓ | -7.71 |  |  |  |
| SMG1 homolog, phosphatidylinositol 3-kinase-related kinase (C. elegans) | 23049 | SMG1 nonsense mediated mRNA decay associated PI3K related kinase | *SMG1* | ↓ | -7.01 |  |  |  |
| nuclear paraspeckle assembly transcript 1 (non-protein coding) | 283131 | nuclear paraspeckle assembly transcript 1 | *NEAT1* | ↓ | -6.69 |  |  |  |
| transient receptor potential cation channel, subfamily M, member 6 | 140803 | transient receptor potential cation channel subfamily M member 6 | *TRPM6* | ↓ | -6.67 |  |  |  |
| matrix metallopeptidase 26 | 56547 | matrix metallopeptidase 26 | *MMP26* | ↓ | -6.46 |  |  |  |
| KRIT1, ankyrin repeat containing | 889 | KRIT1 ankyrin repeat containing | *KRIT1* | ↓ | -6.25 |  |  |  |
| prenylcysteine oxidase 1 | 51449 | prenylcysteine oxidase 1 | *PCYOX1* | ↓ | -6.17 |  |  |  |
| ectonucleotide pyrophosphatase/phosphodiesterase 3 | 5169 | ectonucleotide pyrophosphatase/phosphodiesterase 3 | *ENPP3* | ↓ | -6.12 |  |  |  |
| cysteine-rich secretory protein 3 | 10321 | cysteine rich secretory protein 3 | *CRISP3* | ↓ | -6.06 |  |  |  |
| DEAD (Asp-Glu-Ala-Asp) box polypeptide 17 | 10521 | DEAD-box helicase 17 | *DDX17* | ↓ | -6.02 |  |  |  |
| amylase, alpha 1A (salivary) | 276 | amylase alpha 1A (salivary) | *AMY1A* | ↓ | -6.01 |  |  |  |
| amylase, alpha 2A (pancreatic) | 279 | amylase alpha 2A (pancreatic) | *AMY2A* | ↓ | -6.01 |  |  |  |
| amylase, alpha 2B (pancreatic) | 280 | amylase alpha 2B (pancreatic) | *AMY2B* | ↓ | -6.01 |  |  |  |
| DnaJ (Hsp40) homolog, subfamily C, member 3 | 5611 | DnaJ heat shock protein family (Hsp40) member C3 | *DNAJC3* | ↓ | -5.91 |  |  |  |
| cell wall biogenesis 43 C-terminal homolog (S. cerevisiae) | 80157 | cell wall biogenesis 43 C-terminal homolog | *CWH43* | ↓ | -5.87 |  |  |  |
| cysteine protease; ICErel-III | 838 | caspase 5 | *CASP5* | ↑ | 100 | 0.0469 (p values) | (MS) phase | [35]  Transcriptomics level |
| putative mono-ADP-ribosyltransferase (htMART), GenBank: U47054. | / | / | */* | ↑ | 100 | 0.0156 |  |  |
| RNA-binding protein CUG-BP/hNab50; NAB50 | 10658 | CUGBP Elav-like family member 1 | *CELF1* | ↑ | 100 | 0.0080 |  |  |
| bile salt export pump; BSEP | 8647 | ATP binding cassette subfamily B member 11 | *ABCB11* | ↑ (Northern blot validated) | 100 | 0.0365 |  |  |
| VDAC1 pseudogene | 642585 | voltage dependent anion channel 1 pseudogene 1 | *VDAC1P1* | ↑ | 100 | 0.0156 |  |  |
| ZIC2 protein (ZIC2) | 7546 | Zic family member 2 | *ZIC2* | ↑ | 100 | 0.0280 |  |  |
| carbonic anhydrase I (CAI) | 759 | carbonic anhydrase 1 | *CA1* | ↑ | 100 | 0.0015 |  |  |
| PMS7 mRNA (yeast mismatch repair gene PMS1 homologue) | / | / | */* | ↑ | 100 | 0.0080 |  |  |
| DNA primase (subunit p58) | 5558 | DNA primase subunit 2 | *PRIM2* | ↑ | 100 | 0.0113 |  |  |
| leukocyte (α) interferon | 3452 | interferon alpha 21 | *IFNA21* | ↑ | 100 | 0.0211 |  |  |
| transforming growth factor β-3 | 7043 | transforming growth factor beta 3 | *TGFB3* | ↑ | 100 | 0.0156 |  |  |
| secretogranin II gene | 7857 | secretogranin II | *SCG2* | ↑ | 100 | 0.0113 |  |  |
| von Hippel-Lindau tumor suppressor (VHL) gene | 7428 | von Hippel-Lindau tumor suppressor | *VHL* | ↑ | 100 | 0.0056 |  |  |
| serine/threonine kinase GenBank:tigr: HG2709-HT2805 | / | / | */* | ↑ | 100 | 0.0156 |  |  |
| ELK receptor tyrosine kinase ligand GenBank: L37361 | / | / | */* | ↑ | 100 | 0.0365 |  |  |
| S100E calcium binding protein | 6274 | S100 calcium binding protein A3 | *S100A3* | ↓ | 100 | 0.0365 |  |  |
| Rab9 expressed pseudogene GenBank:U44105 | 9366 | RAB9B, member RAS oncogene family pseudogene 1 | *RAB9BP1* | ↓ | 100 | 0.0365 |  |  |
| PCAF-associated factor 65 α | 10629 | TATA-box binding protein associated factor 6 like | *TAF6L* | ↓ | 100 | 0.0365 |  |  |
| HGF activator-like protein. GenBank: D49742 | / | / | */* | ↓ | 100 | 0.0080 |  |  |
| Cytoplasmic antiproteinase 2 (CAP2) | 5271 | serpin family B member 8 | *SERPINB8* | ↓ | 100 | 0.0024 |  |  |
| GTPase-activating protein (rap1GAP). GenBank: M64788 | / | / | */* | ↓ | 100 | 0.0280 |  |  |
| ras Interactor (RIN1) mRNA | 9610 | Ras and Rab interactor 1 | *RIN1* | ↓ | 100 | 0.0365 |  |  |
| protein kinase (JNK1) | 5599 | mitogen-activated protein kinase 8 | *MAPK8* | ↓ | 100 | 0.0469 |  |  |
| guanylate cyclase GenBank: L13436 | / | / | */* | ↓ | 100 | 0.0211 |  |  |
| T-lymphocyte specific protein tyrosine kinase p56lck (lck) aberrant mRNA. GenBank: U23852 | 3932 | LCK proto-oncogene, Src family tyrosine kinase | *LCK* | ↓ | 100 | 0.0211 |  |  |
| protein tyrosine phosphatase (LPTPase). GenBank: M64322 | / | / | */* | ↓ | 100 | 0.0280 |  |  |
| SHB mRNA | 6461 | SH2 domain containing adaptor protein B | *SHB* | ↓ | 100 | 0.0156 |  |  |
| activating NK-receptor (NK-p46) | 9437 | natural cytotoxicity triggering receptor 1 | *NCR1* | ↓ | 100 | 0.0156 |  |  |
| monocyte chemotactic protein-2 GenBank:Y16645 | 6355 | C-C motif chemokine ligand 8 | *CCL8* | ↓ | 100 | 0.0156 |  |  |
| BENE mRNA | 7851 | mal, T cell differentiation protein like | *MALL* | ↓ | 100 | 0.0211 |  |  |
| FOSB (GenBank Accession: NM_006732) | 2354 | FosB proto-oncogene, AP-1 transcription factor subunit | *FOSB* | ↑ | 44.89 | 0.0052 | (MS) phase | [29] |
| IL6 (NM_000600) | 3569 | interleukin 6 | *IL6* | ↑ | 21.11 | 0.0057 |  |  |
| ATF3 (NM_001040619) | 467 | activating transcription factor 3 | *ATF3* | ↑ | 19.87 | 0.0011 |  |  |
| EGR3 (NM_004430) | 1960 | early growth response 3 | *EGR3* | ↑ | 19.13 | 0.0009 |  |  |
| HPCAL4 (NM_016257) | 51440 | hippocalcin like 4 | *HPCAL4* | ↑ | 18.51 | 0.0169 |  |  |
| RGS1 (NM_002922) | 5996 | regulator of G protein signaling 1 | *RGS1* | ↑ | 16.71 | 0.0006 |  |  |
| CXCL2 (NM_002089) | 2920 | C-X-C motif chemokine ligand 2 | *CXCL2* | ↑ | 14.97 | 0.0002 |  |  |
| FOS (NM_005252) | 2353 | Fos proto-oncogene, AP-1 transcription factor subunit | *FOS* | ↑ | 12.14 | 0.0101 |  |  |
| NR4A3 (NM_173200) | 8013 | nuclear receptor subfamily 4 group A member 3 | *NR4A3* | ↑ | 11.72 | 0.0333 |  |  |
| NR4A1 (NM_002135) | 3164 | nuclear receptor subfamily 4 group A member 1 | *NR4A1* | ↑ | 11.10 | 0.0312 |  |  |
| KRT5 (NM_000424) | 3852 | keratin 5 | *KRT5* | ↑ | 10.54 | 0.0052 |  |  |
| CYR61 (NM_001554) | 3491 | cellular communication network factor 1 | *CCN1* | ↑ | 10.13 | 0.0025 |  |  |
| EGR1 (NM_001964) | 1958 | early growth response 1 | *EGR1* | ↑ | 10.08 | 0.0190 |  |  |
| SOCS3 (NM_003955) | 9021 | suppressor of cytokine signaling 3 | *SOCS3* | ↑ | 9.94 | 0.0001 |  |  |
| EGR2 (NM_000399) | 1959 | early growth response 2 | *EGR2* | ↑ | 8.61 | 0.0007 |  |  |
| CRABP1 (NM_004378) | 1381 | cellular retinoic acid binding protein 1 | *CRABP1* | ↓ | 57.58 | 0.0014 |  |  |
| FMN2 (NM_020066) | 56776 | formin 2 | *FMN2* | ↓ | 16.73 | 0.0353 |  |  |
| GALP (NM_033106) | 85569 | galanin like peptide | *GALP* | ↓ | 15.39 | 0.0054 |  |  |
| PTPLA (NM_014241) | 9200 | 3-hydroxyacyl-CoA dehydratase 1 | *HACD1* | ↓ | 7.32 | 0.0231 |  |  |
| CA12 (NM_001218) | 771 | carbonic anhydrase 12 | *CA12* | ↓ | 6.96 | 0.0180 |  |  |
| SP3P (BC036697) | 160824 | Sp3 transcription factor pseudogene | *SP3P* | ↓ | 6.81 | 0.0335 |  |  |
| (DA825750) | / | / | */* | ↓ | 6.79 | 0.0006 |  |  |
| PITX1 (NM_002653) | 5307 | paired like homeodomain 1 | *PITX1* | ↓ | 6.15 | 0.0337 |  |  |
| AGT(NM_000029) | 183 | angiotensinogen | *AGT* | ↓ | 5.88 | 0.0048 |  |  |
| CCBE1 (NM_133459) | 147372 | collagen and calcium binding EGF domains 1 | *CCBE1* | ↓ | 5.75 | 0.0414 |  |  |
| ENST00000539178 | / | / (novel transcript) | */* | ↓ | 5.72 | 0.0328 |  |  |
| KCNK2 (NM_001017424) | 3776 | potassium two pore domain channel subfamily K member 2 | *KCNK2* | ↓ | 5.37 | 0.0250 |  |  |
| LRRD1 (NM_001161528) | 401387 | leucine rich repeats and death domain containing 1 | *LRRD1* | ↓ | 5.25 | 0.0042 |  |  |
| DDIT4L (NM_145244) | 115265 | DNA damage inducible transcript 4 like | *DDIT4L* | ↓ | 5.17 | 0.0395 |  |  |
| KRTAP19-2 (NM_181608) | 337969 | keratin associated protein 19-2 | *KRTAP19-2* | ↓ | 5.17 | 0.0192 |  |  |
| NEDD1 (Gene ID: ENSG00000139350) | 121441 | NEDD1 gamma-tubulin ring complex targeting factor | *NEDD1* | ↑ | 15.82 | 0.0441 (p values) | (P) phase | [38]  Transcriptomics level |
| PIGP (ENSG00000185808) | 51227 | phosphatidylinositol glycan anchor biosynthesis class P | *PIGP* | ↑ | 12.62 | 0.0219 |  |  |
| HLA-A (ENSG00000206503) | 3105 | major histocompatibility complex, class I, A | *HLA-A* | ↑ | 7.68 | 0.0278 |  |  |
| RPLP2 (ENSG00000177600) | 6181 | ribosomal protein lateral stalk subunit P2 | *RPLP2* | ↑ | 5.84 | 0.0115 |  |  |
| MUC5B (ENSG00000117983) | 727897 | mucin 5B, oligomeric mucus/gel-forming | *MUC5B* | ↑ | 5.53 | 0.0136 |  |  |
| BPIFB1 (ENSG00000125999) | 92747 | BPI fold containing family B member 1 | *BPIFB1* | ↑ | 3.97 | 0.0099 |  |  |
| NKX6-1 (ENSG00000163623) | 4825 | NK6 homeobox 1 | *NKX6-1* | ↑ | 2.98 | 0.0233 |  |  |
| CXCL10 (ENSG00000169245) | 3627 | C-X-C motif chemokine ligand 10 | *CXCL10* | ↑ | 2.92 | 5.00E-05 |  |  |
| IGJ (ENSG00000132465) | 3512 | joining chain of multimeric IgA and IgM | *JCHAIN* | ↑ | 2.89 | 0.0002 |  |  |
| CRISP2 (ENSG00000124490) | 7180 | cysteine rich secretory protein 2 | *CRISP2* | ↑ | 2.68 | 0.0057 |  |  |
| PDE5A (ENSG00000138735) | 8654 | phosphodiesterase 5A | *PDE5A* | ↑ | 2.51 | 0.0085 |  |  |
| CDK19 (ENSG00000155111) | 23097 | cyclin dependent kinase 19 | *CDK19* | ↑ | 2.41 | 0.0336 |  |  |
| NEFM (ENSG00000104722) | 4741 | neurofilament medium | *NEFM* | ↑ | 2.41 | 0.0150 |  |  |
| RSAD2 (ENSG00000134321) | 91543 | radical S-adenosyl methionine domain containing 2 | *RSAD2* | ↑ | 2.41 | 5.00E-05 |  |  |
| CCL19 (ENSG00000172724) | 6363 | C-C motif chemokine ligand 19 | *CCL19* | ↑ | 2.32 | 0.0410 |  |  |
| CHCHD5 (ENSG00000125611) | 84269 | coiled-coil-helix-coiled-coil-helix domain containing 5 | *CHCHD5* | ↓ | -4.56 | 0.0291 |  |  |
| FLRT2 (ENSG00000185070) | 23768 | fibronectin leucine rich transmembrane protein 2 | *FLRT2* | ↓ | -3.59 | 0.0140 |  |  |
| CLEC18B (ENSG00000140839) | 497190 | C-type lectin domain family 18 membrane B | *CLEC18B* | ↓ | -3.29 | 0.0114 |  |  |
| OPRK1 (ENSG00000082556) | 4986 | opioid receptor kappa 1 | *OPRK1* | ↓ | -3.07 | 5.00E-05 |  |  |
| FAM118A (ENSG00000100376) | 55007 | family with sequence similarity 118 member A | *FAM118A* | ↓ | -2.91 | 0.0249 |  |  |
| CALB2 (ENSG00000172137) | 794 | calbindin 2 | *CALB2* | ↓ | -2.31 | 5.00E-05 |  |  |
| KCNK13 (ENSG00000152315) | 56659 | potassium two pore domain channel subfamily K member 13 | *KCNK13* | ↓ | -2.28 | 0.0008 |  |  |
| OR1L4 (ENSG00000136939) | 254973 | olfactory receptor family 1 subfamily L member 4 | *OR1L4* | ↓ | -2.18 | 0.0126 |  |  |
| PENK (ENSG00000181195) | 5179 | proenkephalin | *PENK* | ↓ | -2.12 | 0.0032 |  |  |
| AC104057.1 (ENSG00000269558) | / | / (retired) | */* | ↓ | -2.06 | 0.0227 |  |  |
| SLC26A4 (ENSG00000091137) | 5172 | solute carrier family 26 member 4 | *SLC26A4* | ↓ | -1.94 | 0.0080 |  |  |
| SH3TC1 (ENSG00000125089) | 54436 | SH3 domain and tetratricopeptide repeats 1 | *SH3TC1* | ↓ | -1.91 | 0.0349 |  |  |
| PPFIA4 (ENSG00000143847) | 8497 | PTPRF interacting protein alpha 4 | *PPFIA4* | ↓ | -1.85 | 0.0162 |  |  |
| CDHR5 (ENSG00000099834) | 53841 | cadherin related family member 5 | *CDHR5* | ↓ | -1.78 | 0.0142 |  |  |
| HSD17B2 (ENSG00000086696) | 3294 | hydroxysteroid 17-beta dehydrogenase 2 | *HSD17B2* | ↓ | -1.77 | 0.0109 |  |  |
| ENST00000433673 (SeqName) AC068282.3 (GeneSymbol) | 100506922 | MAP3K2 divergent transcript | *MAP3K2-DT* | ↑ (qPCR validated) | 31.37 | 0.00001 (p values) | (LS) phase | [27]  ncRNomics level  (only top 10 up/down differential lncRNAs available in the article) |
| TCONS_00008629 (XLOC_004134) | / | / | */* | ↑ | 19.50 | 0.0013 |  |  |
| ENST00000513638 (GBP1P1) | 400759 | guanylate binding protein 1 pseudogene 1 | *GBP1P1* | ↑ (qPCR validated) | 19.42 | 0.0036 |  |  |
| ENST00000554711 (RP11-369C8.1) | 105370479 | long intergenic non-protein coding RNA 2303 | *LINC02303* | ↑ (qPCR validated) | 19.39 | 0.0004 |  |  |
| ENST00000554810 (RP11-369C8.1) | 105370479 | long intergenic non-protein coding RNA 2303 | *LINC02303* | ↑ | 19.20 | 0.0012 |  |  |
| uc004bit.1 (AX746484) | / | / | */* | ↑ | 17.32 | 0.0031 |  |  |
| uc001uzl.3 (BC025370) | / | / | */* | ↑ | 11.53 | 0.0378 |  |  |
| ENST00000436680 (BX571672.1) | / | / (retired) | */* | ↑ | 10.41 | 0.0434 |  |  |
| TCONS_00008628 (XLOC_004134) | / | / | */* | ↑ | 9.62 | 0.0469 |  |  |
| ENST00000437859 (RP3-417L20.4) | / | / | */* | ↑ | 9.56 | 0.0094 |  |  |
| ENST00000445708 (RP11-403H13.1) | / | / | */* | ↓ | 44.30 | 0.0403 |  |  |
| ENST00000511064 (RP11-679C8.2) | / | / | */* | ↓ | 26.86 | 0.0016 |  |  |
| ENST00000556770 (RP11-77A13.1) | / | / | */* | ↓ | 20.77 | 0.0366 |  |  |
| ENST00000568243 (RP11-408H20.1) | / | / (retired) | */* | ↓ | 18.37 | 0.00004 |  |  |
| ENST00000428176 (CHRM3-AS2) | 100506915 | CHRM3 antisense RNA 2 | *CHRM3-AS2* | ↓ | 16.88 | 0.0002 |  |  |
| ENST00000451547 (AC007246.3) | 728730 | MAP4K3 divergent transcript | *MAP4K3-DT* | ↓ (qPCR validated) | 15.95 | 0.0021 |  |  |
| ENST00000450063 (AC006159.3) | 100996266 | long intergenic non-protein coding RNA 1510 | *LINC01510* | ↓ | 14.60 | 0.0024 |  |  |
| ENST00000455395 (FTX) | 100302692 | FTX transcript, XIST regulator | *FTX* | ↓ (qPCR validated) | 14.33 | 0.0001 |  |  |
| Uc002rrs.1 (LOC728730) | / | / | */* | ↓ | 13.67 | 0.0015 |  |  |
| ENST00000422736 (RP11-557H15.3) | / | / | */* | ↓ | 13.26 | 0.0001 |  |  |
| TCONS_00006582 (transcript ID), XLOC_013291 (Gene ID) | / | / | */* | ↑ | 3.23 | 0.0443 (p values) | (P) phase | [38]  ncRNomics level |
| ENST00000365494.1, ENSG00000263934.2,  SNORD3A (Gene symbol) | 780851 | small nucleolar RNA, C/D box 3A | *SNORD3A* | ↑ | 3.01 | 5.00E-05 |  |  |
| ENST00000571722.2, ENSG00000262074.3, SNORD3B-2 | 780852 | small nucleolar RNA, C/D box 3B-2 | *SNORD3B-2* | ↑ | 2.88 | 5.00E-05 |  |  |
| ENST00000580729.1, ENSG00000266176.1, RP11-855A2.5 | / | / | */* | ↑ | 2.21 | 0.0332 |  |  |
| ENST00000569473.1 ENSG00000261441.1 RP11-217B1.2 | / | / | */* | ↑ (qPCR validated) | 2.21 | 0.0004 |  |  |
| ENST00000430694.1, ENSG00000231486.3, AC096579.7 | / | / | */* | ↑ | 2.02 | 5.00E-05 |  |  |
| TCONS_01438906, XLOC_414548 | / | / | */* | ↑ | 2.00 | 0.0279 |  |  |
| TCONS_03095195, XLOC_780581 | / | / | */* | ↑ | 1.78 | 5.00E-05 |  |  |
| ENST00000584923.1, ENSG00000263934.2, SNORD3A | 780851 | small nucleolar RNA, C/D box 3A | *SNORD3A* | ↑ | 1.67 | 0.0005 |  |  |
| ENST00000548900.1, ENSG00000257764.2, RP11-1143G9.4 | / | / | */* | ↑ (qPCR validated) | 1.65 | 0.0284 |  |  |
| ENST00000506222.2, ENSG00000249346.2, LINC01016 | 100507584 | LINC01016 | *LINC01016* | ↑ | 1.61 | 0.0159 |  |  |
| ENST00000602478.1, ENSG00000270022.2, RNU12 | 267010 | RNA, U12 small nuclear | *RNU12* | ↑ | 1.57 | 0.0003 |  |  |
| TCONS_00092361, XLOC_011990 | / | / | */* | ↑ | 1.52 | 5.00E-05 |  |  |
| ENST00000557359.1, ENSG00000258548.1, LINC00645 | 100505967 | long intergenic non-protein coding RNA 645 | *LINC00645* | ↑ | 1.51 | 0.0003 |  |  |
| TCONS_02272346; XLOC_631125 | / | / | */* | ↑ | 1.50 | 0.0320 |  |  |
| TCONS_08347373, XLOC_2270304 | / | / | */* | ↓ | -4.11 | 0.0243 |  |  |
| TCONS_08337131, XLOC_2249017 | / | / | */* | ↓ | -3.28 | 0.0012 |  |  |
| TCONS_07594578, XLOC_2090654 | / | / | */* | ↓ | -3.27 | 0.0382 |  |  |
| TCONS_00586906, XLOC_143998 | / | / | */* | ↓ | -2.90 | 0.0465 |  |  |
| ENST00000453660.2, ENSG00000175164.9, ABO | 28 | ABO, alpha 1-3-N-acetylgalactosaminyltransferase and alpha 1-3-galactosyltransferase | *ABO* | ↓ | -1.99 | 0.0282 |  |  |
| ENST00000500447.1, ENSG00000247095.2, MIR210HG | 100506211 | MIR210 host gene | *MIR210HG* | ↓ | -1.86 | 0.0054 |  |  |
| ENST00000555481.1, ENSG00000258642.1, RP11-219E7.2 | / | / | */* | ↓ | -1.49 | 0.0297 |  |  |
| ENST00000570230.1, ENSG00000260528.2, FAM157C | 100996541 | family with sequence similarity 157 member C | *FAM157C* | ↓ | -1.46 | 0.0418 |  |  |
| ENST00000560239.1, ENSG00000259244.1, RP11-182J1.12 | / | / | */* | ↓ (qPCR validated) | -1.43 | 0.0047 |  |  |
| TCONS_06246626, XLOC_1591778 | / | / | */* | ↓ | -1.42 | 0.0447 |  |  |
| TCONS_05992731, XLOC_1591772 | / | / | */* | ↓ | -1.41 | 0.0476 |  |  |
| ENST00000430537.1, ENSG00000227017.1, AC007036.6 | / | / | */* | ↓ | -1.41 | 0.0202 |  |  |
| ENST00000582300.2, ENSG00000265752.2, RP11-403A21.1 | / | / | */* | ↓ | -1.34 | 0.0422 |  |  |
| ENST00000552602.1, ENSG00000244306.5, CTD-2314B22.3 | / | / | */* | ↓ | -1.26 | 0.0089 |  |  |
| TCONS_03829539, XLOC_1065449 | / | / | */* | ↓ | -1.21 | 0.0108 |  |  |
| hsa-miR-885-5p (MIMAT0004947) | (100126334) | microRNA 885 | *(MIR885)* | ↑ (ns by RT-PCR validation) | 2.02 | N/S | (P) phase | [42]  ncRNomics level |
| hsa-miR-24 (Accession number MIMAT0000080) | (407012) | microRNA 24-1 | *(MIR24-1)* | ↑ (ns by RT-PCR validation) | 1.11 |  |  |  |
| hsa-miR-144* (MIMAT0004600) | (406936) | microRNA 144 | *(MIR144)* | ↓ (ns by RT-PCR validation) | -3.31 |  |  |  |
| hsa-miR-106b* (MIMAT0004672) | (406900) | microRNA 106b | *(MIR106B)* | ↓ (ns by RT-PCR after validation) | -2.53 |  |  |  |
| hsa-miR-675 (MIMAT0004284) | (100033819) | microRNA 675 | *(MIR675)* | ↓ (ns by RT-PCR validation) | -2.42 |  |  |  |
| hsa-miR-145* (MIMAT0004601) | (406937) | microRNA 145 | *(MIR145)* | ↓ (ns by RT-PCR validation) | -2.30 |  |  |  |
| hsa-miR-26b (MIMAT0000083) | (407017) | microRNA 26b | *(MIR26B)* | ↓ (ns by RT-PCR validation) | -2.19 |  |  |  |
| hsa-miR-497 (MIMAT0002820) | (574456) | microRNA 497 | *(MIR497)* | ↓ (ns by RT-PCR validation) | -2.12 |  |  |  |
| hsa-miR-222* (MIMAT0004569) | (407007) | microRNA 222 | *(MIR222)* | ↓ (ns by RT-PCR validation) | -2.06 |  |  |  |
| has-miR-29b (MIMAT0000100) | (407024) | microRNA 29b-1 | *(MIR29B1)* | ↓ (ns by RT-PCR validation) | -1.79 |  |  |  |
| hsa-miR-483-5p (MIMAT0004761 | 619552 | microRNA 483 | *MIR483* | ↓ (RT-PCR validated) | -1.77 |  |  |  |
| hsa-miR-142-3p (MIMAT0000434) | (406934) | microRNA 142 | *(MIR142)* | ↓ (ns by RT-PCR validation) | -1.66 |  |  |  |
| hsa-miR-185 (MIMAT0000455) | (406961) | microRNA 185 | *(MIR185)* | ↓ (ns by RT-PCR validation) | -1.54 |  |  |  |
| hsa-let-7b (MIMAT0000063) | (406884) | microRNA let-7b | *(MIRLET7B)* | ↓ (ns by RT-PCR validation) | -1.37 |  |  |  |
| hsa-miR-629* (MIMAT0003298) | 693214 | microRNA 629 | *MIR629* | ↓ (RT-PCR validated) | -1.24 |  |  |  |
| hsa-miR-3685 | 100500802 | microRNA 3685 | *MIR3685* | ↑ | 2.68 | 0.0040 (p values used) | (P) phase | [41]  ncRNomics level |
| RNU5 | / | / (unspecific) | */* | ↑ | 2.31 | 0.0060 |  |  |
| hsa-miR-3667-5p | (100500882) | microRNA 3667 | *(MIR3667)* | ↑ (ns by RT-PCR validation) | 2.30 | 0.0100 |  |  |
| hsa-miR-3156-3p | 100422988 | microRNA 3156-1 | *MIR3156-1* | ↑ | 2.30 | 0.0120 |  |  |
| hsa-miR-4284 | (100422948) | microRNA 4284 | *(MIR4284)* | ↑ (ns by RT-PCR validation) | 2.29 | 0.0150 |  |  |
| hsa-miR-4764-3p | 100616295 | microRNA 4764 | *MIR4764* | ↑ | 2.23 | 0.0260 |  |  |
| SNORD44 | 26806 | small nucleolar RNA, C/D box 44 | *SNORD44* | ↑ | 2.22 | 0.0080 |  |  |
| hsa-miR-3680-5p | (100500917) | microRNA 3680-1 | *(MIR3680-1)* | ↑ (ns by RT-PCR validation) | 2.21 | 0.0070 |  |  |
| hsa-miR-4639-3p | 100616269 | microRNA 4639 | *MIR4639* | ↑ | 2.12 | 0.0050 |  |  |
| hsa-miR-5704 | 100847040 | microRNA 5704 | *MIR5704* | ↑ | 2.11 | 0..0130 |  |  |
| hsa-miR-23b-3p | 407011 | microRNA 23b | *MIR23B* | ↑ | 2.10 | 0.0180 |  |  |
| SNORD38B (small nucleolar RNA, C/D box 38B) | 94163 | small nucleolar RNA, C/D box 38B | *SNORD38B* | ↑ | 2.06 | 0.0040 |  |  |
| hsa-miR-199b-5p | 406978 | microRNA 199b | *MIR199B* | ↑ | 1.98 | 0.0090 |  |  |
| hsa-miR-27a-3p | 407018 | microRNA 27a | *MIR27A* | ↑ | 1.95 | 0.0340 |  |  |
| RNU1 | 26871 | RNA, U1 small nuclear 1 | *RNU1-1* | ↑ | 1.91 | 0.0070 |  |  |
| hsa-miR-4717-3p | 100616241 | microRNA 4717 | *MIR4717* | ↓ | 0.68 | 0.0020 |  |  |
| hsa-miR-4740-3p | 100616294 | microRNA 4740 | *MIR4740* | ↓ | 0.69 | 0.0050 |  |  |
| hsa-miR-5703 | 100847081 | microRNA 5703 | *MIR5703* | ↓ | 0.69 | 0.0020 |  |  |
| hsa-miR-3157-5p | 100422892 | microRNA 3157 | *MIR3157* | ↓ | 0.70 | 0.0020 |  |  |
| hcmv-miR-UL70-3p | / | / | */* | ↓ | 0.70 | 0.0040 |  |  |
| hsa-miR-5581-5p | 100847010 | microRNA 5581 | *MIR5581* | ↓ | 0.71 | 0.0050 |  |  |
| hsa-miR-5188 | 100847004 | microRNA 5188 | *MIR5188* | ↓ | 0.71 | 0.0070 |  |  |
| hsa-miR-3124-5p | 100422879 | microRNA 3124 | *MIR3124* | ↓ | 0.72 | 0.0070 |  |  |
| hsa-miR-548ao-5p | 100847068 | microRNA 548ao | *MIR548AO* | ↓ | 0.73 | 0.0130 |  |  |
| hsa-miR-4690-5p | 100616292 | microRNA 4690 | *MIR4690* | ↓ | 0.73 | 0.0040 |  |  |
| hsa-miR-4730 | 100616359 | microRNA 4730 | *MIR4730* | ↓ | 0.73 | 0.0080 |  |  |
| hsa-miR-548g-3p | 100313938 | microRNA 548g | *MIR548G* | ↓ | 0.74 | 0.0030 |  |  |
| hsa-miR-4689 | 100616421 | microRNA 4689 | *MIR4689* | ↓ | 0.74 | 0.0040 |  |  |
| hsa-miR-549a | 693132 | microRNA 549a | *MIR549A* | ↓ | 0.74 | 0.0070 |  |  |
| hsa-miR-4515 | 100616404 | microRNA 4515 | *MIR4515* | ↓ | 0.74 | 0.0020 |  |  |
| miR-34c-5p (MIMAT0000686) | 407042 | microRNA 34c | *MIR34C* | ↓ (qRT-PCR validated) | -2.96 | 0.015 | (ES) phase | [40]  ncRNomics level |
| miR-34b* (MIMAT0000685) | 407041 | microRNA 34b | *MIR34B* | ↓ (qRT-PCR validated) | -2.84 | 0.019 |  |  |
| miR-34c-3p (MIMAT0004677) | 407042 | microRNA 34c | *MIR34C* | ↓ | -2.54 | 0.025 |  |  |
| miR-9 (MIMAT0000441) | 407046 | microRNA 9-1 | *MIR9-1* | ↓ (qRT-PCR validated) | -1.90 | 0.0032 |  |  |
| miR-9* (MIMAT0000442) | (407046) | microRNA 9-1 | *(MIR9-1)* | ↓ (ns by qRT-PCR validation) | -1.90 | 0.0152 |  |  |
| miRPlus_42780 (unannotated) | / | / | */* | ↓ | -1.79 | 0.038 |  |  |
| hsa-miR-148b* | 442892 | microRNA 148b | *MIR148B* | ↓ | 0.17 | 0.00 | (P) phase | [43]  ncRNomics level |
| hsv1-miR-H4* | / | / | */* | ↓ | 0.18 | 0.01 |  |  |
| hsa-miR-363* | 574031 | mircoRNA 363 | *MIR363* | ↓ | 0.20 | 0.01 |  |  |
| hsa-miRPlus-E1027 | / | / | */* | ↓ | 0.22 | 0.02 |  |  |
| hsa-miR-668 | 768214 | microRNA 668 | *MIR668* | ↓ | 0.22 | 0.00 |  |  |
| hsa-miR-206 | 406989 | microRNA 206 | *MIR206* | ↓ | 0.23 | 0.01 |  |  |
| hsa-miR-922 | 100126321 | microRNA 922 | *MIR922* | ↓ | 0.24 | 0.01 |  |  |
| ebv-miR-BART18-3p | / | / | */* | ↓ | 0.24 | 0.04 |  |  |
| hsa-miR-374b* | 100126317 | microRNA 374b | *MIR374B* | ↓ | 0.26 | 0.00 |  |  |
| hsa-miRPlus-F1212 | / | / | */* | ↓ | 0.26 | 0.03 |  |  |
| hsa-miRPlus-C1100 | / | / | */* | ↓ | 0.27 | 0.02 |  |  |
| ebv-miR-BHRF1-3 | / | / | */* | ↓ | 0.28 | 0.02 |  |  |
| hsa-miRPlus-E1049 | / | / | */* | ↓ | 0.30 | 0.02 |  |  |
| hsa-miRPlus-E1215 | / | / | */* | ↓ | 0.31 | 0.04 |  |  |
| hsa-miRPlus-E1082 | / | / | */* | ↓ | 0.33 | 0.03 |  |  |
| miR-411* | 693121 | microRNA 411 | *MIR411* | ↑ (qRT-PCR validated) | 1.74 | 0.0199 (p values) | N/S phase | [39]  ncRNomics level |
| miR-4289 | 100423015 | microRNA 4289 | *MIR4289* | ↑ | 1.58 | 0.0041 |  |  |
| miR-578 | 693163 | microRNA 578 | *MIR578* | ↑ | 1.58 | 0.0054 |  |  |
| miR-138 | 406929 | microRNA 138-1 | *MIR138-1* | ↑ | 1.50 | 0.0286 |  |  |
|  | 406930 | microRNA 138-2 | *MIR138-2* |  |  |  |  |  |
| miR-380* | 494329 | microRNA 380 | *MIR380* | ↑ | 1.41 | 0.0227 |  |  |
| miR-1201 | 100113391 | small nucleolar RNA, C/D box 126 | *SNORD126* | ↑ | 1.37 | 0.0485 |  |  |
| miR-4295 | 100422909 | microRNA 4295 | *MIR4295* | ↑ | 1.34 | 0.0040 |  |  |
| miR-3167 | 100422918 | microRNA 3167 | *MIR3167* | ↑ | 1.32 | 0.0200 |  |  |
| miR-518d-5p | 574489 | microRNA 518d | *MIR518D* | ↑ | 1.32 | 0.0121 |  |  |
| miR-139-3p | 406931 | microRNA 139 | *MIR139* | ↑ | 1.22 | 0.0412 |  |  |
| miR-337-5p | 442905 | microRNA 337 | *MIR337* | ↑ | 1.06 | 0.0441 |  |  |
| miR-373* | 442918 | microRNA 373 | *MIR373* | ↓ | -1.60 | 0.0109 |  |  |
| miR-636 | 693221 | microRNA 636 | *MIR636* | ↓ (qRT-PCR validated) | -1.52 | 0.0489 |  |  |
| miR-935 | 100126325 | microRNA 935 | *MIR935* | ↓ (qRT-PCR validated) | -1.48 | 0.0154 |  |  |
| miR-556-3p | 693141 | microRNA 556 | *MIR556* | ↓ (qRT-PCR validated) | -1.42 | 0.0011 |  |  |
| miR-892b | 100126307 | MicroRNA 892b | *MIR892B* | ↓ | -1.37 | 0.0114 |  |  |
| miR-4312 | 100422971 | microRNA 4312 | *MIR4312* | ↓ | -1.34 | 0.0207 |  |  |
| miR-1224-3p | 100187716 | microRNA 1224 | *MIR1224* | ↓ | -1.30 | 0.0096 |  |  |
| miR-4260 | 100422894 | microRNA 4260 | *MIR4260* | ↓ | -1.28 | 0.0107 |  |  |
| miR-3130-5p | 100422993 | microRNA 3130-1 | *MIR3130-1* | ↓ | -1.27 | 0.0304 |  |  |
|  | 100423002 | microRNA 3130-2 | *MIR3130-2* |  |  |  |  |  |
| hsa-miR-196a-5p | 406972 | microRNA 196a-1 | *MIR196A1* | ↑ (qPCR validated) | 16.85 | 0.0070 | (MS) phase | [29]  ncRNomics level |
|  | 406973 | microRNA 196a-2 | *MIR196A2* |  | 16.85 | 0.0070 |  |  |
| hsa-miR-3196 | 100423014 | microRNA 3196 | *MIR3196* | ↑ | 12.03 | 0.0406 |  |  |
| hsa-miR-135a-3p | 406925 | microRNA 135a-1 | *MIR135A1* | ↑ | 9.14 | 0.0168 |  |  |
| hsa-miR-5585-3p | 100847018 | microRNA 5585 | *MIR5585* | ↑ | 7.09 | 0.0094 |  |  |
| hsa-miR-194-3p | 406970 | microRNA 194-2 | *MIR194-2* | ↑ | 7.07 | 0.0258 |  |  |
| hsa-miR-1915-5p | 100302129 | microRNA 1915 | *MIR1915* | ↑ | 6.48 | 0.0008 |  |  |
| hsa-miR-138-5p | 406929 | microRNA 138-1 | *MIR138-1* | ↑ | 6.08 | 0.0106 |  |  |
|  | 406930 | microRNA 138-2 | *MIR138-2* |  |  |  |  |  |
| hsv1-miR-H7-3p | / | / | */* | ↑ | 5.86 | 0.0322 |  |  |
| hsa-miR-1273a | / | / (withdrawn) | */* | ↑ | 5.71 | 0.0022 |  |  |
| hsv1-miR-H14-3p | / | / | */* | ↑ | 5.29 | 0.0028 |  |  |
| hsa-miR-921 | 100126349 | microRNA 921 | *MIR921* | ↑ | 4.98 | 0.0304 |  |  |
| hsa-miR-4425 | 100616365 | microRNA 4425 | *MIR4425* | ↑ | 4.92 | 0.0330 |  |  |
| hsa-miR-365b-5p | 100126356 | microRNA 365b | *MIR365B* | ↑ | 4.64 | 0.0225 |  |  |
| hsa-miR-542-5p | 664617 | microRNA 542 | *MIR542* | ↑ | 4.59 | 0.0234 |  |  |
| hsa-miR-4251 | 100422968 | microRNA 4251 | *MIR4251* | ↑ | 4.56 | 0.0160 |  |  |
| hsa-miR-339-5p | 442907 | microRNA 339 | *MIR339* | ↓ | 0.16 | 0.0374 |  |  |
| hsa-miR-3686 | 100500839 | microRNA 3686 | *MIR3686* | ↓ | 0.31 | 0.0396 |  |  |
| hsa-miR-4254 | 100423028 | microRNA 4254 | *MIR4254* | ↓ | 0.34 | 0.0247 |  |  |
| hsa-miR-26b-3p | 407017 | microRNA 26b | *MIR26B* | ↓ | 0.35 | 0.0473 |  |  |
| kshv-miR-K12-1-3p | / | / | */* | ↓ | 0.35 | 0.0299 |  |  |
| hsa-miR-374b-5p | 100126317 | microRNA 374b | *MIR374B* | ↓ | 0.38 | 0.0450 |  |  |
| hsa-miR-4252 | 100422975 | microRNA 4252 | *MIR4252* | ↓ | 0.38 | 0.0277 |  |  |
| hsa-miR-22-5p | 407004 | microRNA 22 | *MIR22* | ↓ | 0.39 | 0.0139 |  |  |
| hsa-miR-548aa/hsa-miR-548ap-3p/hsa-miR-548t-3p | 100500895 | microRNA 548aa-2 | *MIR548AA2* | ↓ | 0.43 | 0.0283 |  |  |
|  | 100847084 | microRNA 548ap | *MIR548AP* |  |  |  |  |  |
|  | 100422849 | microRNA 548t | *MIR548T* |  |  |  |  |  |
| hsa-miR-219b-5p | 100616335 | microRNA 219b | *MIR219B* | ↓ | 0.44 | 0.0251 |  |  |
| hsa-miR-505-3p | 574508 | microRNA 505 | *MIR505* | ↓ | 0.45 | 0.0345 |  |  |
| hsa-miR-4723-5p | 100616388 | microRNA 4723 | *MIR4723* | ↓ | 0.47 | 0.0127 |  |  |
| chromatin accessibility complex protein 1 (DNA polymerase epsilon subunit p15) | 54108 | chromatin accessibility complex subunit 1 | *CHRAC1* | ↑ | 77.3 (ration between E *vs*. C) | N/S | (S) phase | [48]  Proteomics level |
| actin-like-7-alpha | 10881 | actin like 7A | *ACTL7A* | ↑ | 22.5 |  |  |  |
| cyclin A1 | 8900 | cyclin A1 | *CCNA1* | ↑ | Expressed only in E. |  |  |  |
| 14-3-3 protein sigma | 2810 | stratifin | *SFN* | ↑ | Expressed only in E. |  |  |  |
| protein C14 orf 48 | 256369 | coiled-coil domain containing 197 | *CCDC197* | ↓ | 18.3 |  |  |  |
| cytidine deaminase (Cytidine aminohydrolase) | 978 | cytidine deaminase | *CDA* | ↓ | 15.8 |  |  |  |
| CD166 antigen (activated leukocyte-cell adhesion molecule) | 214 | activated leukocyte cell adhesion molecule | *ALCAM* | ↓ | 12.9 |  |  |  |
| protein C14 orf 138 | 79609 | valosin containing protein lysine methyltransferase | *VCPKMT* | ↓ | 6.41 |  |  |  |
| NTF2-related export protein 2 | 55916 | nuclear transport factor 2 like export factor 2 | *NXT2* | ↓ | Absent in E |  |  |  |
| mitochondrial import receptor subunit TOM22 homolog (translocase of outer membrane 22 kDa subunit homolog) | 56993 | translocase of outer mitochondrial membrane 22 | *TOMM22* | ↓ | Absent in E. |  |  |  |
| polyadenylate-binding protein-interacting protein 2 (poly(A) binding protein-interacting protein 2) | 51247 | poly(A) binding protein interacting protein 2 | *PAIP2* | ↓ | Absent in E. |  |  |  |
| tubulin α-1c chain | 84790 | tubulin alpha 1c | *TUBA1C* | ↑ | 2.10 | All statistically significant (p ≤ 0.05) | (MS) phase | [46]  Proteomics level |
| histone-binding protein RBBP4 | 5928 | RB binding protein 4, chromatin remodeling factor | *RBBP4* | ↑ | 1.90 |  |  |  |
| pyruvate dehydrogenase E1 component subunit β | 5162 | pyruvate dehydrogenase E1 beta subunit | *PDHB* | ↑ | 1.90 |  |  |  |
| ribonuclease/angiogenin inhibitor 1 | 6050 | ribonuclease/angiogenin inhibitor 1 | *RNH1* | ↑ and ↓ (IHC validated) | 1.20 |  |  |  |
| vimentin | 7431 | vimentin | *VIM* | ↑ | 1.20 |  |  |  |
| peroxiredoxin-6 | 9588 | peroxiredoxin 6 | *PRDX6* | ↑ in DIGE and ↓ (Western blotting validated) | 1.40; -2.70 |  |  |  |
| myosin regulatory light chain 12A | 10627 | myosin light chain 12A | *MYL12A* | ↓ | -10.40 |  |  |  |
| transgelin-2 | (8407) | transgelin 2 | *(TAGLN2)* | ↓ (ns by IHC validation) | -4.50 |  |  |  |
| annexin A2 | 302 | annexin A2 | *ANXA2* | ↓ | -3.10 |  |  |  |
| vimentin | 7431 | vimentin | *VIM* | ↓ (validated by Western blot) | -3.0 |  |  |  |
| eukaryotic initiation factor 4A-I | 1973 | eukaryotic translation initiation factor 4A1 | *EIF4A1* | ↓ | -2.20 |  |  |  |
| Eukaryotic initiation factor 4A-II | 1974 | eukaryotic translation initiation factor 4A2 | *EIF4A2* | ↓ | -2.20 |  |  |  |
| annexin A5 | 308 | annexin A5 | *ANXA5* | ↓ | -2.10 |  |  |  |
| keratin 18 | 3875 | keratin 18 | *KRT18* | ↓ | -2.10 |  |  |  |
| endoplasmin | 7184 | heat shock protein 90 beta family member 1 | *HSP90B1* | ↓ | -2.00 |  |  |  |
| 14-3-3 protein epsilon | 7531 | tyrosine 3-monooxygenase/tryptophan 5-monooxygenase activation protein epsilon | *YWHAE* | ↓ | -1.90 |  |  |  |
| aconitase 2 precursor | 50 | aconitase 2 | *ACO2* | ↓ | -1.90 |  |  |  |
| gelsolin precursor | 2934 | gelsolin | *GSN* | ↓ | -1.80 |  |  |  |
| mitochondrial inner membrane protein | 10989 | inner membrane mitochondrial protein | *IMMT* | ↓ | -1.80 |  |  |  |
| coronin-1B | 57175 | coronin 1B | *CORO1B* | ↓ | -1.80 |  |  |  |
| T-complex protein 1 subunit theta | 10694 | chaperonin containing TCP1 subunit 8 | *CCT8* | ↓ | -1.80 |  |  |  |
| alpha 2 type VI collagen isoform 2C2 precursor | 1292 | collagen type VI alpha 2 chain | *COL6A2* | ↑ | 1.22 (sta. II); 1.67 (sta. III); 3.15 (sta. IV) | All statistically significant (p < 0.05) | (S) phase | [30]  Proteomics level |
| gelsolin isoform a precursor | 2934 | gelsolin | *GSN* | ↑ | 2.62 (sta.II); 1.22 (sta.III); 1.08 (sta.IV) |  |  |  |
| tubulin, beta (gi*\|57209813) | 2039068 | tubulin beta class I | *TUBB* | ↑ | 1.62 (sta.II); 1.86 (sta.III); 3.03 (sta.IV) |  |  |  |
| actin, beta (gi\|14250401) | 60 | actin beta | *ACTB* | ↑ | 1.35 (sta.II); 2.02 (sta. III); 2.14 (sta.IV) |  |  |  |
| tropomyosin 4 isoform 2 | 7171 | tropomyosin 4 | *TPM4* | ↑ | 1.41 (sta.II); 4.24 (sta.III); 5.20 (sta.IV) |  |  |  |
| GRP78 precursor. (pod gi\|386758) | 3309 | heat shock protein family A (Hsp70) member 5 | *HSPA5* | ↑ (immunoblot validated) | 1.56 (sta.II); 1.77 (sta.III); 2.46 (sta.IV) |  |  |  |
| heat shock 70 kDa protein 8 isoform 1 | 3312 | heat shock protein family A (Hsp70) member 8 | *HSPA8* | ↑ (immunoblot validated) | 1.70 (sta.II); 2.15 (sta.III); 2.35 (sta.IV) |  |  |  |
| mitochondrial heat shock 60 kDa protein 1 (gi\|189502784) | 3329 | heat shock protein family D (Hsp60) member 1 | *HSPD1* | ↑ (immunoblot validated) | 1.05 (sta.II); 1.53 (sta.III); 2.61 (sta.IV) |  |  |  |
| heat shock protein beta-1 | 3315 | heat shock protein family B (small) member 1 | *HSPB1* | ↑ (immunoblot validated) | 1.28 (sta.II); 2.79 (sta.III); 3.24 (sta.IV) |  |  |  |
| chain A, crystal structure of human DJ-1 (gi\|42543006) | 11315 | Parkinsonism associated deglycase | *PARK7* | ↑ (immunoblot validated) | 1.78 (sta.II); 2.42 (sta.III); 2.77 (sta.IV) |  |  |  |
| annexin A5 | 308 | annexin A5 | *ANXA5* | ↑ | 1.88 (sta.II); 2.64 (sta.III); 3.03 (sta. IV) |  |  |  |
| NADH dehydrogenase (ubiquinone) Fe-S protein 1, 75 kDa | 4719 | NADH:ubiquinone oxidoreductase core subunit S1 | *NDUFS1* | ↑ | 1.15 (sta.II); 1.33 (sta.III); 3.10 (sta.IV) |  |  |  |
| 14-3-3 Protein Epsilon (gi\|67464424) | 7531 | tyrosine 3-monooxygenase/tryptophan 5-monooxygenase activation protein epsilon | *YWHAE* | ↑ | 2.22 (sta.II); 2.34 (sta.III); 2.68 (sta.IV) |  |  |  |
| lamin B2 (gi\|27436951) | 84823 | lamin B2 | *LMNB2* | ↑ | 1.38 (sta.II); 1.72 (sta.III); 2.25 (sta.IV) |  |  |  |
| lamin B1 | 4001 | lamin B1 | *LMNB1* | ↑ | 1.12 (sta.II); 1.61 (sta.III); 2.21 (sta.IV) |  |  |  |
| vinculin, isoform CRA_a (gi\|119574932) | 7414 | vinculin | *VCL* | ↓ | -1.21 (sta.II); -1.80 (sta.III); -2.8 (sta.IV) |  |  |  |
| vimentin | 7431 | vimentin | *VIM* | ↓ | -1.08 (sta.II); -1.31 (sta.III);  -1.5 (sta.IV) |  |  |  |
| beta actin variant (gi\|62897409) | 60 | actin beta | *ACTB* | ↓ | -1.56 (sta.II);  -1.62 (sta.III);  -4.25 (sta.IV) |  |  |  |
| F-actin capping protein beta subunit | 832 | capping actin protein of muscle Z-line subunit beta | *CAPZB* | ↓ | -1.22 (sta.II);  1.49 (sta.III);  -2.93 (sta.IV) |  |  |  |
| heat shock 70 kDa protein 9B precursor (gi\|62897075) | 3313 | heat shock protein family A (Hsp70) member 9 | *HSPA9* | ↓ | -1.15 (sta.II);  -1.78 (sta.III);  -1.88 (sta.IV) |  |  |  |
| mitochondrial heat shock 60 kDa protein 1 | 3329 | heat shock protein family D (Hsp60) member 1 | *HSPD1* | ↓ | -1.03 (sta.II);  -1.94 (sta.III);  -2.57 (sta.IV) |  |  |  |
| chaperonin containing TCP1, subunit 8 (theta) (gi\|62896539) | 10694 | chaperonin containing TCP1 subunit 8 | *CCT8* | ↓ | -1.10 (sta.II);  -1.31 (sta.III);  -1.77 (sta.IV) |  |  |  |
| protein disulfide isomerase family A, member 3, isofrom cra_a (gi\|119597640) | 2923 | protein disulfide isomerase family A member 3 | *PDIA3* | ↓ (immunoblot validated) | -1.20 (sta.II);  -1.78 (sta.III);  -2.5 (sta.IV) |  |  |  |
| T-complex polypeptide 1 | 6950 | t-complex 1 | *TCP1* | ↓ | -1.10 (sta.II);  -1.39 (sta.III);  -2.37 (sta.IV) |  |  |  |
| peroxiredoxin 2 isoform a (gi\|32189392) | 7001 | peroxiredoxin 2 | *PRDX2* | ↓ | -1.14 (sta.II);  -2.10 (sta.III);  -3.40 (sta.IV) |  |  |  |
| peroxiredoxin 3, isoform CRA_c (gi\|119569783) | 10935 | peroxiredoxin 3 | *PRDX3* | ↓ | -1.10 (sta.II);  -1.53 (sta.III);  -1.94 (sta.IV) |  |  |  |
| annexin A4 (gi\|39645467) | 307 | annexin A4 | *ANXA4* | ↓ | -1.38 (sta.II);  -1.56 (sta.III);  -3.18 (sta.IV) |  |  |  |
| glucosidase, alpha; neutral AB, isoform CRA_a (gi\|119594451) | 23193 | glucosidase II alpha subunit | *GANAB* | ↓ | -1.04 (sta.II);  -1.30 (sta.III);  -2.20 (sta.IV) |  |  |  |
| mitochondrial ATP synthase, H+ transporting F1 complex beta subunit (gi\|89574029) | 506 | ATP synthase F1 subunit beta | *ATP5F1B* | ↓ | -1.07 (sta.II);  -1.19 (sta.III);  -1.96 (sta.IV) |  |  |  |
| guanine nucleotide binding protein (G protein), beta polypeptide | 2782 | G protein subunit beta 1 | *GNB1* | ↓ | -1.11 (sta.II);  -1.64 (sta.III);  -2.00 (sta.IV) |  |  |  |
| peroxiredoxin 3, isoform CRA_c (gi\|119569783) | 10935 | peroxiredoxin 3 | *PRDX3* | ↓ | -6.40 (sta.IV) | All statistically significant (p < 0.05) | (P) |  |
| T-complex polypeptide 1 | 6950 | t-complex 1 | *TCP1* | ↓ | -4.56 (sta.IV) |  |  |  |
| vimentin | 7431 | vimentin | *VIM* | ↓ | -3.85 (sta.IV) |  |  |  |
| F-actin capping protein beta subunit | 832 | capping actin protein of muscle Z-line subunit beta | *CAPZB* | ↓ | -3.05 (sta.IV) |  |  |  |
| mitochondrial ATP synthase, H+ transporting F1 complex beta subunit (gi\|89574029) | 506 | ATP synthase F1 subunit beta | *ATP5F1B* | ↓ | -2.52 (sta.IV) |  |  |  |
| protein disulfide isomerase family A, member 3, isofrom cra_a (gi\|119597640) | 2923 | protein disulfide isomerase family A member 3 | *PDIA3* | ↓ (IHC and immunoblot validated) | -2.36 (sta.IV) |  |  |  |
| chaperonin containing TCP1, subunit 8 (theta) (gi\|62896539) | 10694 | chaperonin containing TCP1 subunit 8 | *CCT8* | ↓ | -2.24 (sta.IV) |  |  |  |
| heat shock 70 kDa protein 9B precursor (gi\|62897075) | 3313 | heat shock protein family A (Hsp70) member 9 | *HSPA9* | ↓ | -1.72 (sta.IV) |  |  |  |
| vinculin, isoform CRA_a (gi\|119574932) | 7414 | vinculin | *VCL* | ↓ | -1.72 (sta.IV) |  |  |  |
| beta actin variant (gi\|62897409) | 60 | actin beta | *ACTB* | ↓ | -1.69 (sta.IV) |  |  |  |
| glucosidase, alpha; neutral AB, isoform CRA_a (gi\|119594451) | 23193 | glucosidase II alpha subunit | *GANAB* | ↓ | -1.64 (sta.IV) |  |  |  |
| annexin A4 (gi\|39645467) | 307 | annexin A4 | *ANXA4* | ↓ | -1.62 (sta.IV) |  |  |  |
| peroxiredoxin 2 isoform a (gi\|32189392) | 7001 | peroxiredoxin 2 | *PRDX2* | ↓ | -1.48 (sta.IV) |  |  |  |
| lamin B2 (gi\|27436951) | 84823 | lamin B2 | *LMNB2* | ↓ | -1.43 (sta.IV) |  |  |  |
| annexin A5 | 308 | annexin A5 | *ANXA5* | ↓ | -1.36 (sta.IV) |  |  |  |
| tropomyosin 4 isoform 2 (gi\|4507651) | 7171 | tropomyosin 4 | *TPM4* | ↑ | 4.14 (sta.IV) |  |  |  |
| GRP78 precursor. (pod gi\|386758) | 3309 | heat shock protein family A (Hsp70) member 5 | *HSPA5* | ↑ (IHC and immunoblot validated) | 3.46 (sta.IV) |  |  |  |
| NADH dehydrogenase (ubiquinone) Fe-S protein 1, 75 kDa (gi\|21411235) | 4719 | NADH:ubiquinone oxidoreductase core subunit S1 | *NDUFS1* | ↑ | 3.54 (sta.IV) |  |  |  |
| major vault protein (gi\|194389978) | 9961 | major vault protein | *MVP* | ↑ (IHC and immunoblot validated) | 2.86 (sta.IV) |  |  |  |
| valosin-containing protein (gi\|111305821) | 7415 | valosin containing protein | *VCP* | ↑ | 2.64 (sta.IV) |  |  |  |
| heat shock protein beta-1 | 3315 | heat shock protein family B (small) member 1 | *HSPB1* | ↑ (IHC and immunoblot validated) | 2.50 (sta.IV) |  |  |  |
| vimentin | 7431 | vimentin | *VIM* | ↑ | 2.26 (sta.IV) |  |  |  |
| Motor protein (gi\|516764) | 10989 | inner membrane mitochondrial protein | *IMMT* | ↑ | 2.13 (sta.IV) |  |  |  |
| mitochondrial heat shock 60 kDa protein 1 | 3329 | heat shock protein family D (Hsp60) member 1 | *HSPD1* | ↑ (IHC and immunoblot validated) | 2.07 (sta.IV) |  |  |  |
| actin, beta (gi\|14250401) | 60 | actin beta | *ACTB* | ↑ | 1.98 (sta.IV) |  |  |  |
| alpha 2 type VI collagen isoform 2C2 precursor | 1292 | collagen type VI alpha 2 chain | *COL6A2* | ↑ | 1.98 (sta.IV) |  |  |  |
| guanine nucleotide binding protein (G protein), beta polypeptide | 2782 | G protein subunit beta 1 | *GNB1* | ↑ | 1.92 (sta.IV) |  |  |  |
| tubulin, beta (gi\|57209813) | 203068 | tubulin beta class I | *TUBB* | ↑ | 1.89 (sta.IV) |  |  |  |
| B23 nucleophosmin (280 AA) (gi\|825671) | 4869 | nucleophosmin 1 | *NPM1* | ↑ | 1.82 (sta.IV) |  |  |  |
| Chain A, crystal structure of human DJ-1 | 11315 | Parkinsonism associated deglycase | *PARK7* | ↑ (IHC and immunoblot validated) | 1.78 (sta.IV) |  |  |  |
| serine/threonine kinase pisslre (S49330) | 8558 | cyclin dependent kinase 10 | *CDK10* | ↑ | Expressed greatly up-regulated in E | All statistically significant (p < 0.05) | (S) | [47]  Proteomics level |
| hypothetical protein ARPC2 (Q53R19_HUMAN) | / | (unreviewed ARPC2 in UniProt) | */* | ↑ |  |  |  |  |
| microtubule-associated protein 6 (Q6ZWB8_HUMAN) | 4135 | microtubule associated protein 6 | *MAP6* | ↑ |  |  |  |  |
| vimentin variant (VIME_HUMAN) | 7431 | vimentin | *VIM* | ↑ |  |  |  |  |
| prefoldin (Q5TBX4_HUMAN) | / | / | */* | ↑ |  |  |  |  |
| heat shock protein 70 (B48127) | / | / (uspecific) | */* | ↑ |  |  |  |  |
| heat shock protein HSP 90-beta | 3326 | heat shock protein 90 alpha family class B member 1 | *HSP90AB1* | ↑ |  |  |  |  |
| immunoglobulin heavy chain VHDJ region frag (BAC01520) | / | / | */* | ↑ |  |  |  |  |
| interferon stimulated exonuclease gene 20kDa-like 1 | 64782 | apoptosis enhancing nuclease | *AEN* | ↑ |  |  |  |  |
| tropomysin isoform mRNA (AAF87083) | 7170 | tropomyosin 3 | *TPM3* | ↑ |  |  |  |  |
| translation elongation factor EF-TU precursor (S62767) | 7284 | Tu translation elongation factor, mitochondrial | *TUFM* | ↑ |  |  |  |  |
| cDNA FLJ41346 fis, clone BRAWH2005315, moderately similar to neuronal STOP protein (Q62WB8_HUMAN) | / | /  UniProt: uncharacterized protein | */* | ↑ |  |  |  |  |
| FHA domain, Forkhead- associated (FHA) phosphopeptide binding domain 1 | 114827 | forkhead associated phosphopeptide binding domain 1 | *FHAD1* | ↑ |  |  |  |  |
| NUANCE (AAL33548) | 23224 | spectrin repeat containing nuclear envelope protein 2 | *SYNE2* | ↑ |  |  |  |  |
| bullous pemphigoid antigen 1, 230/240kDa (Q5TBT2_HUMAN) | 667 | dystonin | *DST* | ↑ |  |  |  |  |
| kelch-like ECH-associated protein1 (cytosolic inhibitor of Nrf2) (Q9BPY9_HUMAN) | 9817 | kelch like ECH associated protein 1 | *KEAP1* | ↓ | -11.50 |  |  |  |
| hypothetical protein fragment (Q961351_HUMAN) | / | / | */* | ↓ | -11.00 |  |  |  |
| H. Sapiens truncated nuclear factor of Kappa light chain polypeptide gene enhancer in B-cells inhibitor, epsilon (NKFBIE) gene (AAV31776) | 4794 | NFKB inhibitor epsilon | *NFKBIE* | ↓ | -1.60 |  |  |  |
| human centrosome protein (BAA32290) | 9696 | ciliary rootlet coiled-coil, rootletin | *CROCC* | ↓ | -1.40 |  |  |  |
| pregnancy-associated endometrial a2-globulin (glycodelin A, PP14) (P09466) | (5047) | progestagen associated endometrial protein | *(PAEP)* | ↑ (ns by western blot validation) | Expressed only in E | N/S | (S) | [44]  Proteomics level |
| vimentin (P08670) | 7431 | vimentin | *VIM* | ↓ (western blot validated) | Absent in E |  |  |  |
| heat shock protein HSP-90α (P07900) | 3320 | heat shock protein 90 alpha family class A member 1 | *HSP90AA1* | ↓ | Absent in E |  |  |  |
| heat shock protein HSP-90β (P08238) | 3326 | heat shock protein 90 alpha family class B member 1 | *HSP90AB1* | ↓ | -3.50 |  |  |  |
| albumin homolog, fragment (7441762 from NCBI) | / | / | */* | ↓ | Absent in E |  |  |  |
| β-Actin, cytoplasmic 1 (P60709) | 60 | actin beta | *ACTB* | ↓ | Absent in E |  |  |  |
| annexin A2 (P07355) | 302 | annexin A2 | *ANXA2* | ↓ | -5.00 |  |  |  |
| β-Actin (P60709) | 60 | actin beta | *ACTB* | ↑ | 5.00 | p < 0.05 | (P) |  |
| prolyl 4-hydroxylase b-subunit (PDI) (P07237) | 5034 | prolyl 4-hydroxylase subunit beta | *P4HB* | ↑ | 4.70 |  |  |  |
| prohibitin (P35232) | 5245 | prohibitin | *PHB* | ↑ | 3.90 |  |  |  |
| actin-related protein 3 homologue (P61158) | 10096 | actin related protein 3 | *ACTR3* | ↑ | 3.60 |  |  |  |
| haemoglobin Chain-D (NCBI: 999576) | / | / | */* | ↓ | -5.50 |  |  |  |
| vimentin (P08670) | 7431 | vimentin | *VIM* | ↓ | -3.70 |  |  |  |
| peroxiredoxin 2 isoform A (Prx-2) (P32119) | 7001 | peroxiredoxin 2 | *PRDX2* | ↓ | -3.30 |  |  |  |
| APOA1 (P02647) | 335 | apolipoprotein A1 | *APOA1* | ↓ | -3.10 |  |  |  |
| collapsin response mediator protein 2 (CRMP2), (Q16555) | 1808 | dihydropyrimidinase like 2 | *DPYSL2* | ↓ (Western blot validated) | 4.09 | N/S | (M) | [45] |
| ubiquitin carboxyl-terminal hydrolase isozyme L1 (UCH-L1), (P09936) | 7345 | ubiquitin C-terminal hydrolase L1 | *UCHL1* | ↓ | 3.10 |  |  |  |
| myosin regulatory light polypeptide 9 (MYL9), (P24844) | 10398 | myosin light chain 9 | *MYL9* | ↓ | 4.68 |  |  |  |

*Abbreviations: UG = NCBI UniGene; gb = NCBI GenBank; gi = NCBI protein, ns = not significant, N/S = not specified in source reference

In the 1^st^ column in the gene catalogue, extracted differentially expressed genes at the RNA (mRNAs, lncRNAs, sncRNAs) and protein levels are listed with the same names, symbols, synonym or accession identification numbers (IDs) as in the source reference. In 2^nd^, 3^rd^, and 4^th^ column, for each locus Gene ID number, gene symbol and corresponding name, respectively are listed. In the 5^th^ column, measured dysregulation from the source reference is marked with “↑” for up- and “↓” for down-regulated expression levels, respectively. Additionally, terms “hypometh” for hypomethylated or “hypermeth” for hypermethylated are used when dysregulated expression levels of extracted genes were detected by the epigenomic study approach. In addition, the information regarding additional validation analysis using qPCR, immunohistochemistry or western blot of genome-wide screening findings in the source reference is marked. Term “ns” refers to not significant after validation analysis. In the 6^th^ and 7^th^ column, reported fold change for expression (expre.) and methylation (methy.) and corresponding statistical value, respectively, are provided. Term “sta.” referes to stage of endometriosis. In the 8^th^ column, the phase of the menstrual cycle is listed when difference of eutopic endometrium between women with and without endometriosis was measured. Each phase of the cycle in gene catalogue is marked with a unique colour: M-phase with grey, P-phase with blue, S-phase with orange, ES-phase with green, MS-phase with red, LS-phase with violet, and N/S (not specified) with grey-white. In the 9^th^ column, the source reference is provided.
