# Supplementary material for: Molecular signature of eutopic endometrium in endometriosis based on the multi-omics integrative synthesis
Source: J Assist Reprod Genet. 2020 May 30;37(7):1593–611. doi: 10.1007/s10815-020-01833-3 (PMC7376782; doi:10.1007/s10815-020-01833-3)
Supplement: Supplementary file 2 — (DOCX 32 kb). [file 10815_2020_1833_MOESM2_ESM.docx]

**Supplementary file S2:** Excluded genome-wide studies from the present study.

| **Reference** | **Indication and clinical characteristicsof participating women in the studies** | **Number of endometriosis (E) samples used for genome-wide analysis in studied phase of the cycle. Stage and type of E.** | **Number of control samples used for genome-wide analysis in studied phase of the cycle, Phenotype.** | **-Endometrial tissue sample sampling**  **- Sample preservation**  **-Nucleic acid or protein isolation** | **Platform for genome-wide analysis** | **Genetic causes associated with E** | **Reason to be excluded** |
| --- | --- | --- | --- | --- | --- | --- | --- |
| **Genomics level** | | | | | | |  |
| [51] | Laparoscopy or laparotomy (cases), elective abortions (controls). | P = 4, S = 1. Stage II-IV: ovarian E (n=3, peritoneal E (n=1; bilateral ovarian E (n=1) | N/S = 4 | - N/S  - fresh-frozen  - Epithelial and stromal cells obtained by laser capture microdissection (LCM). Genomic DNA: phenol/chloroform | Human BAC array | Chromosome loss: 1p36, 1p12, and 22q12, Chromosome gains: 3p14, 10q26 and 13q33 | Expression analysis not performed. LCM-harvesting of cells. |
| [52] | N/S | N/S = 16. Laparoscopically confirmed ovarian E. | N/S = 5. Healthy. | - N/S  - fresh-frozen  - Epithelial and stromal cells obtained by laser capture microdissection (LCM). Genomic DNA: phenol/chloroform | Illumina Hiseq 2000 with 100 bp paired-ends | 6,421 synonymous and 12,458 nonsynonymous mutations in E. 1,437 synonymous and 3,215 nonsynonymous mutations in C. No common mutation between E and C. | Negative association between genetic changes in endometrium and E. LCM-harvesting of cells. |
| **Epigenomic level** | | | | | | |  |
| [53] | Laparoscopy with confirmed E (cases), laparoscopy for pelvic pain, subfertility or tubal sterilisation and confirmed non-E (controls) | M = 4, P = 2, ES = 7, MS = 9, LS = 9. Stage I-V E: ovarian E (n=4), only peritoneal lesions (n=17), peritoneal + ovarian E (n=10) | M = 1, P = 3, ES = 1, MS = 17, LS = 2. Self-reported healthy volunteers and | - Suction Pipelle catheter  - N/S  - N/S | Infinium HumanMethylation 450K BeadChip | 1 Hyper-methylation in MS | Negative association between altered methylome in eutopic endometrium and E. Expression analysis not performed. |
| **Epigenomic level with gene expression validation** | | | | | | | |
| [54] | Laparoscopy | N/S = 8 | N/S = 8. Symptomatic, non-endometriotic adhesion (n=4) and non-endometriotic cyst (n=1) with pelvic pain (n=4) and subfertility (n=4). | - N/S  - N/S  - genomic DNA: Qiagen DNeasy Kits. RNA: Qiagen RNeasy Kit. | Methylation 450K Array  HT12v4.0 Expression Array | - No differentially methylated probes  - 28 ↑ and 4 ↓ mRNAs | Negative association between altered methylome in endometrium and E.  Datasets of differentially expressed genes not provided. |
| **Transcriptomics level** | | | | | | | |
| [57] | Laparoscopy for infertility and/or pain | M = 14, ES = 17. Stage I-II = 16, stage III-IV = 15. | M = 8, ES = 10. Normal pelvis (n=9). Other pelvic pathology: hydrosalpinx (n=1), ovarian cyst (n=3), pelvic inflammatory disease related adhesions (n= 1), adhesions not related to pelvic inflammatory disease (n=2), parasalpingeale cysten (n=2) | - Pipelle samples  - –80°C  - Total RNA: TRIzol reagent | Affymetrix GeneChip Human Gene I.0 ST Array | No differentially expressed genes between E and C in the same phase of the menstrual cycle | Negative association between altered expression levels in endometrium and E |
| [55] | Laparoscopy or/and laparotomy (cases). Laparoscopic tubal ligation or reversal of tubal sterilization (controls). | P = 3, ES = 3, MS = 3, LS = 3  Stage: Np.  Deep E | P = 3, ES = 3, MS = 3, LS = 3  Fertile. | - Suction Pipelle catheter.  - RNA later, stored at -20°C.  - Epithelial and stromal cells obtained by laser capture microdissection (LCM). RNA: the Picopure RNA extraction kit. | The Clontech Atlashuman 1.2 cDNA expression array | Epithelial cells: P: 3↑ / 1↓. ES: 11↑ / 28↓. MS: 6↑ / 8↓. LS: 5↑ / 20↓  Stromal cells: P: 1↑ / 7↓. ES: 44↑ / 7↓.MS: 12↓. LS: 2↑ / 1↓ | LCM-harvesting of cells. No genome wide platform performed. |
| [56] | Laparoscopic removal of endometriosis (cases) or tubal ligation (controls). | S = 8  Stage III/IV  Pelvic pain and/or infertility: nulligravida (n=4), primigravida (n=3, multigravida (n=1) | S = 8  Healthy fertile with no history of pregnancy | - Pipelle catheter  - Snap frozen in liquid N_2_, stored at -80°C.  - Total RNA: Norgen Biotek Total RNA isolation Kit. | NAnostring nCounter GX Human Immunology V2 platform | 60↑ / 31↓ genes | No genome wide platform performed. |

N/S = not specified phase of the menstrual cycle

N_2_ = nitrogen
