# Supplementary material for: Molecular signature of eutopic endometrium in endometriosis based on the multi-omics integrative synthesis
Source: J Assist Reprod Genet. 2020 May 30;37(7):1593–611. doi: 10.1007/s10815-020-01833-3 (PMC7376782; doi:10.1007/s10815-020-01833-3)
Supplement: Supplementary file 3 — (DOCX 59 kb). [file 10815_2020_1833_MOESM3_ESM.docx]

Supplementary file S3.1: Developed gene lists, sorted according to phases of the menstrual cycle and used for GSEA by DAVID bioinformatics tool.

| **Developed gene list** | **Gene lists** |
| --- | --- |
| M-phase list (3 genes) | *DPYSL2*, *UCHL1,* *MYL9*. |
| P-phase list (188 genes) | *ABO, ACTA2, ACTB, ACTR3, ADCY7, ADH5, AFF4, AKAP12, ANXA4, ANXA5, APOA1, APOBEC3A, ATP5F1B, BPIFB1, CALB2, CALM1, CAPZB, CCL19, CCN1, CCT8, CD163, CD46, CDHR3, CDHR5, CDK10, CDK19, CHCHD5, CLEC18B, COL6A2, CP, CREB3L1, CRISP2, CRYZ, CXCL10, DDX17, DHFR, DIO2, EDN1, EGR1, EIF1, EPHX1, FAIM2, FAM118A, FAM157C, FAM76B, FBN1, FLRT2, FOS, FOSB, FXYD1, G6PC3, GANAB, GATM, GBP1, GNB1, GRAP, GRM4, GSTM1, GSTM5, HAPLN1, HBB, HLA-A, HOXA5, HSD17B2, HSP90AB1, HSPA5, HSPA9, HSPB1, HSPD1, IMMT, JCHAIN, JUNB, KCNK13, LCN2, LINC00645, LINC01016, LMNB2, LONRF2, LTF, MFAP4, MGP, MIR148B, MIR199B, MIR206, MIR210HG, MIR23B, MIR27A, MIR3124, MIR3156-1, MIR3157, MIR363, MIR3685, MIR374B, MIR4515, MIR4639, MIR4689, MIR4690, MIR4717, MIR4730, MIR4740, MIR4764, MIR483, MIR5188, MIR548AO, MIR548G, MIR549A, MIR5581, MIR5703, MIR5704, MIR629, MIR668, MIR922, MLLT3, MTSS2, MUC5B, MVP, MYH10, NDUFS1, NEDD1, NEFM, NFAT5, NKX6-1, NPM1, NR1H3, NTM, OLFML1, OPRK1, OR1L4, OVGP1, P4HB, PAG1, PALLD, PARK7, PCSK5, PCYOX1, PDE5A, PDIA3, PENK, PHB, PIGC, PIGP, PKP4, PPFIA4, PPP1R12B, PQBP1, PRDX2, PRDX3, PTCH1, RAVER1, RIOK3, RNU1-1, RNU12, RPLP2, RPS17, RSAD2, S100A2, S100A8, S100A9, SCNN1A, SF3A2, SH3TC1, SLC26A4, SMC3, SNORD38B, SNORD3A, SNORD3B-2, SNORD44, SOX13, ST8SIA2, TAF1D, TCP1, TDRD7, TEK, TJP2, TMSB4X, TOP2A, TPM1, TPM4, TUBB, VCAN, VCL, VCP, VIM, WFDC1, ZBTB38, ZFP36, ZFP36L2.* |
| S-phase list (81 genes) | *ACTB, ACTL7A, AEN, AHCY, ALCAM, AMY2B, ANXA2, ANXA4, ANXA5, ATP5F1B, CAPZB, CCDC197, CCN1, CCN2, CCNA1, CCT8, CDA, CDK10, CHRAC1, COL18A1, COL4A2, COL5A2, COL6A2, CROCC, DST, DUSP1, DUSP2, EGR1, ELN, FHAD1, FN1, FOS, GANAB, GNB1, GSN, HSP90AA1, HSP90AB1, HSPA5, HSPA8, HSPA9, HSPB1, HSPD1, IFI27, IGFBP2, ITGB4, JUNB, KEAP1, LAMA2, LMNB1, LMNB2, MAP6, NDUFS1, NFKBIE, NR4A1, NUS1P3, NXT2, PAIP2, PARK7, PCSK6, PDGFRA, PDIA3, PRDX2, PRDX3, PTK7, PTP4A1, RPS26, SFN, SYNE2, TCP1, TOMM22, TPM3, TPM4, TUBB, TUFM, TYRO3, UGT2B7, VCL, VCPKMT, VIM, WEE1, YWHAE.* |
| ES-phase list (82 genes) | *ABRAXAS1, ALG13, ANKRD20A11P, ANLN, APOBEC3B, APOD, ASPM, BDH2, CAPN6, CDK1, CFLAR, CPM, CST1, CST4, CTNNB1, CXCL13, CYP4B1, CYTOR, DNAJC19, EGR1, ELN, EPHX1, FAM216B, FAM76B, FOS, FOSB, FZD2, KBTBD6, KCNAB1, KDSR, KIF20A, MALAT1, MCCC2, MCTP2, MED12, MIR34B, MIR34C, MIR9-1, MRPL51, MRPS18C, MT1F, MT1G, MT1L, MT1X, NAA38, NAMPT, NDUFA1, NMT2, NUB1, NUF2, OLFM4, OVGP1, PARVA, PBK, PPP1R10, PTEN, PTK2, RNF7, RRM2, SAP18, SCGB2A2, SCN11A, SDF2, SEC24A, SET, SFRP4, SNTN, SPTAN1, SPTLC1, SPTLC2, TCF12, TMED4, TMEM50A, TOB1, TOP2A, TRPM6, TYMS, U2SURP, UCP2, USP36, WNK2, ZNF827.* |
| MS-phase list (173 genes) | *ABCB11, ABCC3, ACKR1, ACO2, ADGRF1, AFF4, AGT, AIMP1, ALPI, AMY1A, AMY2A, AMY2B, ANXA2, ANXA5, AOC1, ATF3, BST2, C1QA, C1QTNF6, CA1, CA12, CASP5, CCBE1, CCL3, CCL3L1, CCL3L3, CCL8, CCN1, CCT8, CDA, CDK5R1, CELF1, COL12A1, CORO1B, CRABP1, CRISP3, CST7, CTSW, CWH43, CXCL2, CYP3A5, DDIT4L, DDX17, DEPP1, DLG5, DNAJC3, DST, EDNRB, EGR1, EGR2, EGR3, EIF1, EIF4A1, EIF4A2, ENPP3, FMN2, FOS, FOSB, GALP, GSN, GUCY1B1, GZMA, HACD1, HOXA9, HPCAL4, HSP90B1, FNA21, IL6, IMMT, JUNB, KCNK2, KRIT1, KRT18, KRT5, KRTAP19-2, LAMA3, LCK, LONRF2, LPP, LRRD1, LTB4R2, LUZP1, MALL, MAP4, MAPK8, MET, MIR135A1, MIR138-1, MIR138-2, MIR1915, MIR194-2, MIR196A1, MIR196A2, MIR219B, MIR22, MIR26B, MIR3196, MIR339, MIR365B, MIR3686, MIR374B, MIR4251, MIR4252, MIR4254, MIR4425, MIR4723, MIR505, MIR542, MIR548AA2, MIR548AP, MIR548T, MIR5585, MIR921, MMP26, MUC7, MYL12A, NCR1, NEAT1, NFAT5, NR4A1, NR4A3, PAX8, PCSK5, PCYOX1, PDHB, PER1, PITX1, PLEK, PLEKHA2, POMZP3, PRDX6, PRIM2, PRRC2C, PTAFR, RAB9BP1, RBBP4, RGS1, RIF1, RIN1, RNF150, RNH1, RSRP1, S100A3, S100A8, SAP30L, SCG2, SCGB2A2, SEMA3C, SERPINB8, SHB, SLA, SLC15A4, SLC1A1, SLC44A2, SMG1, SOCS3, SON, SP3P, TAF6L, TGFB3, THRAP3, TRIM15, TRPM6, TUBA1C, VDAC1P1, VEGFA, VHL, VIM, YBX1, YBX1P2, YWHAE, ZFP36, ZIC2.* |
| LS-phase list (36 genes) | *BAIAP2, BHLHE40, CDK6, CHRM3-AS2, CPE, CYP2J2, DEFB1, FAM118A, FN1, FTX, GBP1P1, GLI1, HPSE, HPSE2, IL6ST, ITGA2B, JAKMIP1, KMO, LINC01510, LINC02303, LRRN1, MAP3K2-DT, MAP4K3-DT, OGN, ORM1, PCDH17, PTPRR, RDM1, RXFP1, S100P, SCGB1D4, SCGB2A1, SCGB2A2, SHISA6, SNCG, STC1.* |
| N/S-phase list (28 genes) | *CDCA2, DUSP22, ID2, IGSF21, MGMT, MIR1224, MIR138-1, MIR138-2, MIR139, MIR3130-1, MIR3130-2, MIR3167, MIR337, MIR373, MIR380, MIR411, MIR4260, MIR4289, MIR4295, MIR4312, MIR518D, MIR556, MIR578, MIR636, MIR892B, MIR935, SNORD126, ZNF681.* |

Supplementary file S3.2: The main results (top 10 enriched GO-BP, GO-CC, GO-MF terms, and KEGG pathways) after GSEA associated with each phase of the menstrual cycle.

| **Top enriched terems** | | | **Functional term** | **Genes annotated** | ***P* value** | **Bonferroni correction** |
| --- | --- | --- | --- | --- | --- | --- |
| **M-phase gene list:** | | | | | | |
| GO-BP: | | 1. | No output | */* | / | / |
| GO-CC: | | 1. | GO:0043209~myelin sheath | *UCHL1, DPYSL2* | 0.016612 | 0.308255 |
|  | | 2. | GO:0005829~cytosol | *UCHL1, DPYSL2, MYL9* | 0.033081 | 0.522925 |
|  | | 3. | GO:0043025~neuronal cell body | *UCHL1, DPYSL2* | 0.034272 | 0.535692 |
| GO-MF | | 1. | No output | */* | / | / |
| KEGG | | 1. | No output | */* | / | / |
| **P-phase gene list:** | | | | | | |
| GO-BP | 1. | | GO:0006928~movement of cell or subcellular component | *ACTB, ACTR3, TUBB, VIM, HSPB1, TPM1, CAPZB, TPM4, VCL* | 5.25E-07 | 6.69E-04 |
|  | 2. | | GO:0032496~response to lipopolysaccharide | *FOS, S100A8, PENK, EDN1, ADH5, PRDX3, HSPD1, JUNB, CXCL10* | 6.32E-05 | 0.077379743 |
|  | 3. | | GO:0043066~negative regulation of apoptotic process | *PRDX2, PRDX3, ANXA5, ANXA4, PARK7, NPM1, TEK, LTF, HSPB1, HSPA5, HSPD1, FAIM2, HSPA9* | 3.78E-04 | 0.382469055 |
|  | 4. | | GO:0045454~cell redox homeostasis | *P4HB, PDIA3, DIO2, LTF, PRDX2, PRDX3* | 4.29E-04 | 0.421040941 |
|  | 5. | | GO:0043523~regulation of neuron apoptotic process | *GRM4, NPM1, PARK7, FAIM2* | 4.78E-04 | 0.456342303 |
|  | 6. | | GO:0050821~protein stabilization | *HSP90AB1, TCP1, APOA1, PHB, CCT8, HSPD1, PARK7* | 9.06E-04 | 0.685177925 |
|  | 7. | | GO:0010870~positive regulation of receptor biosynthetic process | *HOXA5, EDN1, NR1H3* | 0.001370557 | 0.825992842 |
|  | 8. | | GO:0001649~osteoblast differentiation | *PENK, PHB, CREB3L1, VCAN, JUNB, TPM4* | 0.001671895 | 0.881572045 |
|  | 9. | | GO:0006936~muscle contraction | *FXYD1, ACTA2, TPM1, TPM4, CALM1, VCL* | 0.001895263 | 0.910967446 |
|  | 10. | | GO:0045647~negative regulation of erythrocyte differentiation | *ZFP36, HOXA5, HSPA9* | 0.002324308 | 0.948539577 |
| GO-CC | 1. | | GO:0070062~extracellular exosome | *HSP90AB1, S100A8, PDIA3, S100A9, RPLP2, PRDX2, PRDX3, VCL, ACTR3, APOA1, CD46, LTF, GATM, ACTA2, HLA-A, MGP, CDHR5, SLC26A4, RPS17, GNB1, HSPB1, PCYOX1, MFAP4, MVP, BPIFB1, GANAB, VIM, JCHAIN, ADH5, CAPZB, TPM4, TUBB, COL6A2, HSPA5, SCNN1A, HBB, HSPA9, MLLT3, ACTB, FLRT2, P4HB, TCP1, PHB, FBN1, ANXA5, CRYZ, ANXA4, PARK7, NKX6-1, LCN2, VCP, CCT8, CP, HSPD1, MUC5B, MYH10, CALM1* | 1.15E-12 | 3.30E-10 |
|  | 2. | | GO:0005925~focal adhesion | *ACTB, P4HB, FLRT2, PDIA3, VIM, AKAP12, RPLP2, ANXA5, PALLD, TPM4, VCL, ACTR3, RPS17, CD46, TEK, NPM1, HSPB1, HSPA5, HSPA9* | 1.09E-09 | 3.13E-07 |
|  | 3. | | GO:0031012~extracellular matrix | *P4HB, HAPLN1, GANAB, FBN1, VIM, S100A9, MGP, TUBB, RPS17, COL6A2, HSPB1, VCAN, HSPA5, HSPD1, MFAP4, HSPA9* | 8.13E-09 | 2.33E-06 |
|  | 4. | | GO:0043209~myelin sheath | *ACTB, TCP1, PDIA3, VCP, GNB1, IMMT, PHB, PRDX3, HSPD1, HSPA5, NDUFS1, HSPA9* | 2.36E-08 | 6.78E-06 |
|  | 5. | | GO:0005576~extracellular region | *BPIFB1, S100A8, EDN1, S100A9, JCHAIN, CXCL10, VCL, OLFML1, TUBB, APOA1, TEK, COL6A2, LTF, HBB, P4HB, HAPLN1, OVGP1, CRISP2, FBN1, CCL19, CD163, LCN2, CLEC18B, PENK, TMSB4X, VCAN, CP, MFAP4, ABO, GBP1, CALM1* | 3.45E-06 | 9.89E-04 |
|  | 6. | | GO:0005856~cytoskeleton | *ACTB, TUBB, S100A8, S100A9, VIM, PKP4, TEK, AKAP12, HSPB1, TPM1, CAPZB, TPM4, MVP, VCL* | 5.39E-06 | 0.001546836 |
|  | 7. | | GO:0005829~cytosol | *CDK19, HSP90AB1, PPFIA4, S100A8, GRAP, PPP1R12B, S100A9, VIM, ADH5, RPLP2, PRDX2, PRDX3, TPM1, CAPZB, GSTM5, TPM4, CALB2, VCL, GSTM1, ACTR3, FOS, ZFP36L2, APOA1, NPM1, HBB, ACTB, ZFP36, OVGP1, TCP1, ACTA2, CRYZ, PARK7, SMC3, LCN2, NEDD1, DHFR, VCP, RPS17, RIOK3, GNB1, CCT8, PDE5A, HSPB1, HSPD1, TJP2, GBP1, MYH10, CALM1* | 7.29E-06 | 0.002090073 |
|  | 8. | | GO:0005623~cell | *LCN2, P4HB, PDIA3, DIO2, PRDX2, CP, VCL* | 1.17E-04 | 0.033023478 |
|  | 9. | | GO:0044297~cell body | *TUBB, TCP1, ACTA2, GNB1, CCT8, PARK7* | 1.18E-04 | 0.033394533 |
|  | 10. | | GO:0005615~extracellular space | *ACTB, FLRT2, BPIFB1, S100A8, ACTA2, CRISP2, FBN1, EDN1, S100A9, JCHAIN, CCL19, CXCL10, LCN2, APOA1, COL6A2, LTF, HSPB1, WFDC1, VCAN, CP, HSPD1, PCSK5, MUC5B* | 5.47E-04 | 0.145214942 |
| GO-MF | 1. | | GO:0051082~unfolded protein binding | *HSP90AB1, TCP1, CCT8, NPM1, HSPD1, HSPA5, HSPA9* | 2.75E-04 | 0.097399182 |
|  | 2. | | GO:0005515~protein binding | *HSP90AB1, PDIA3, S100A8, TAF1D, ZNF580, EDN1, S100A9, RPLP2, PRDX3, GSTM5, ZBTB38, CXCL10, VCL, ACTR3, FOS, APOBEC3A, DDX17, APOA1, CD46, PQBP1, CREB3L1, LTF, CDK10, TOP2A, NDUFS1, PAG1, S100A2, EGR1, ZFP36, SOX13, HLA-A, FAM118A, MGP, CDHR5, FAM76B, PALLD, JUNB, CD163, GNB1, RIOK3, HSPB1, VCAN, TMSB4X, MFAP4, GBP1, MVP, PPFIA4, GRAP, TDRD7, OPRK1, PPP1R12B, VIM, AKAP12, RSAD2, TPM1, TPM4, ZFP36L2, TUBB, HOXA5, TEK, NPM1, COL6A2, NFAT5, HSPA5, SCNN1A, PCSK5, HBB, NEFM, NR1H3, MLLT3, HSPA9, ACTB, P4HB, TCP1, IMMT, PHB, FBN1, ANXA5, SF3A2, ANXA4, SMC3, PARK7, VCP, PKP4, CCT8, PTCH1, HSPD1, TJP2, MUC5B, NTM, CALM1, MYH10* | 4.35E-04 | 0.14974498 |
|  | 3. | | GO:0044822~poly(A) RNA binding | *ZFP36, HSP90AB1, P4HB, TCP1, GANAB, PDIA3, IMMT, HLA-A, SF3A2, ZFP36L2, DDX17, VCP, RPS17, RAVER1, NPM1, HSPB1, EIF1, TMSB4X, HSPD1, TOP2A, HSPA9* | 7.81E-04 | 0.252833145 |
|  | 4. | | GO:0019899~enzyme binding | *GSTM1, ZFP36, CDK19, P4HB, APOA1, PHB, HSPA5, TOP2A, PARK7, GBP1* | 0.00159644 | 0.44896009 |
|  | 5. | | GO:0005200~structural constituent of cytoskeleton | *ACTB, ACTR3, TUBB, VIM, TPM1, NEFM* | 0.002026046 | 0.530685327 |
|  | 6. | | GO:0005201~extracellular matrix structural constituent | *HAPLN1, FBN1, MGP, VCAN, MUC5B* | 0.002142524 | 0.550679418 |
|  | 7. | | GO:0001077~transcriptional activator activity, RNA polymerase II core promoter proximal region sequence-specific binding | *EGR1, FOS, HOXA5, NFAT5, CREB3L1, FOSB, JUNB, NR1H3* | 0.003159298 | 0.692808379 |
|  | 8. | | GO:0005509~calcium ion binding | *S100A8, FBN1, S100A9, CDHR3, MGP, CDHR5, ANXA5, ANXA4, CALB2, TPM4, VCAN, HSPA5, CALM1, S100A2* | 0.005577067 | 0.875825652 |
|  | 9. | | GO:0031625~ubiquitin protein ligase binding | *TUBB, TCP1, DIO2, VCP, HSPD1, HSPA5, HSPA9, VCL* | 0.009004929 | 0.965749462 |
|  | 10. | | GO:0019901~protein kinase binding | *ZFP36, HSP90AB1, ACTA2, PPP1R12B, NPM1, HSPB1, PRDX3, MVP, CALM1* | 0.011697724 | 0.987586516 |
| KEGG | 1. | | hsa04915:Estrogen signaling pathway | *HSP90AB1, FOS, ADCY7, CREB3L1, CALM1* | 0.031243339 | 0.995168635 |
|  | 2. | | hsa04668:TNF signaling pathway | *FOS, EDN1, CREB3L1, JUNB, CXCL10* | 0.039873336 | 0.998925533 |
|  | 3. | | hsa05031:Amphetamine addiction | *FOS, CREB3L1, FOSB, CALM1* | 0.045021447 | 0.99956456 |
|  | 4. | | hsa04918:Thyroid hormone synthesis | *SLC26A4, ADCY7, CREB3L1, HSPA5* | 0.052062354 | 0.999874397 |
|  | 5. | | hsa04141:Protein processing in endoplasmic reticulum | *HSP90AB1, P4HB, GANAB, PDIA3, VCP, HSPA5* | 0.05285968 | 0.999890955 |
|  | 6. | | hsa05206:MicroRNAs in cancer | *MIR206, MIR363, VIM, MIR27A, TPM1, MIR483, MIR23B, MIR199B* | 0.056417293 | 0.999942052 |
|  | 7. | | hsa00980:Metabolism of xenobiotics by cytochrome P450 | *GSTM1, ADH5, EPHX1, GSTM5* | 0.059603972 | 0.999967174 |
|  | 8. | | hsa05204:Chemical carcinogenesis | *GSTM1, ADH5, EPHX1, GSTM5* | 0.07182017 | 0.999996351 |
|  | 9. | | hsa05414:Dilated cardiomyopathy | *ACTB, ADCY7, TPM1, TPM4* | 0.080540621 | 0.999999253 |
|  | 10. | | hsa04261:Adrenergic signaling in cardiomyocytes | *ADCY7, CREB3L1, TPM1, TPM4, CALM1* | 0.084865086 | 0.999999662 |
| **S-phase gene list:** | | | | | | |
| GO-BP | 1. | | GO:0006928~movement of cell or subcellular component | *ACTB, TUBB, VIM, HSPB1, CAPZB, TPM4, TPM3, VCL* | 8.11E-08 | 5.82E-05 |
|  | 2. | | GO:0006457~protein folding | *HSP90AB1, TCP1, HSP90AA1, GANAB, PDIA3, GNB1, CCT8, HSPA8, HSPA9* | 9.95E-07 | 7.13E-04 |
|  | 3. | | GO:0098609~cell-cell adhesion | *HSP90AB1, RPS26, CCT8, HSPA5, SFN, YWHAE, CAPZB, PARK7, HSPA8, ANXA2* | 2.28E-06 | 0.0016316 |
|  | 4. | | GO:0030198~extracellular matrix organization | *LAMA2, COL18A1, COL4A2, ELN, COL6A2, ITGB4, COL5A2, FN1* | 2.06E-05 | 0.0146427 |
|  | 5. | | GO:0070527~platelet aggregation | *ACTB, TYRO3, PDGFRA, HSPB1, VCL* | 2.79E-05 | 0.0198098 |
|  | 6. | | GO:0006986~response to unfolded protein | *HSP90AB1, HSP90AA1, HSPB1, HSPD1, HSPA8* | 3.07E-05 | 0.0218022 |
|  | 7. | | GO:0043066~negative regulation of apoptotic process | *DUSP1, HSPB1, PRDX2, HSPD1, PRDX3, HSPA5, ANXA5, ANXA4, PARK7, HSPA9* | 1.36E-04 | 0.0931412 |
|  | 8. | | GO:0007155~cell adhesion | *LAMA2, ALCAM, COL18A1, TYRO3, COL6A2, ITGB4, PTK7, DST, FN1, VCL* | 1.46E-04 | 0.0991565 |
|  | 9. | | GO:0050821~protein stabilization | *HSP90AB1, TCP1, HSP90AA1, CCT8, HSPD1, PARK7* | 2.93E-04 | 0.1895852 |
|  | 10. | | GO:0032870~cellular response to hormone stimulus | *FOS, DUSP1, IGFBP2, JUNB* | 9.43E-04 | 0.4914273 |
| GO-CC | 1. | | GO:0070062~extracellular exosome | *HSP90AB1, TUFM, AHCY, GANAB, PDIA3, CROCC, VIM, ITGB4, PRDX2, PRDX3, SFN, CAPZB, TPM4, TPM3, VCL, ALCAM, RPS26, TUBB, GSN, COL6A2, HSPA5, AMY2B, HSPA8, HSPA9, FN1, ACTB, COL18A1, COL4A2, TCP1, HSP90AA1, ANXA5, YWHAE, ANXA4, PARK7, ANXA2, LAMA2, SYNE2, GNB1, PTP4A1, CCT8, HSPB1, HSPD1, IGFBP2, DST, UGT2B7* | 2.27E-18 | 4.64E-16 |
|  | 2. | | GO:0031012~extracellular matrix | *COL18A1, COL4A2, GANAB, HSP90AA1, VIM, COL5A2, ANXA2, LAMA2, TUBB, COL6A2, HSPB1, HSPA5, HSPD1, PCSK6, HSPA8, FN1, HSPA9* | 3.59E-14 | 7.35E-12 |
|  | 3. | | GO:0043209~myelin sheath | *ACTB, TUFM, TCP1, HSP90AA1, PDIA3, GNB1, HSPD1, PRDX3, HSPA5, NDUFS1, HSPA8, HSPA9* | 2.15E-11 | 4.41E-09 |
|  | 4. | | GO:0005925~focal adhesion | *ACTB, PDIA3, VIM, PTK7, ANXA5, YWHAE, TPM4, VCL, ALCAM, SYNE2, GSN, HSPB1, HSPA5, DST, HSPA8, HSPA9* | 3.37E-11 | 6.92E-09 |
|  | 5. | | GO:0042470~melanosome | *HSP90AB1, HSP90AA1, AHCY, GANAB, PDIA3, HSPA5, YWHAE, HSPA8, ANXA2* | 6.71E-09 | 1.38E-06 |
|  | 6. | | GO:0005913~cell-cell adherens junction | *HSP90AB1, RPS26, CCT8, HSPA5, SFN, YWHAE, CAPZB, PARK7, HSPA8, ANXA2, VCL* | 6.16E-07 | 1.26E-04 |
|  | 7. | | GO:0005856~cytoskeleton | *ACTB, TUBB, ACTL7A, VIM, HSPB1, DST, CAPZB, TPM4, TPM3, VCL* | 1.68E-05 | 0.0034339 |
|  | 8. | | GO:0005829~cytosol | *HSP90AB1, AHCY, NFKBIE, VIM, PRDX2, KEAP1, PRDX3, SFN, CAPZB, TPM4, TPM3, VCL, RPS26, FOS, GSN, CDA, CCNA1, HSPA8, ACTB, TCP1, HSP90AA1, YWHAE, PARK7, ANXA2, GNB1, CCT8, HSPB1, HSPD1, DST* | 3.99E-05 | 0.0081458 |
|  | 9. | | GO:0016020~membrane | *ACTB, HSP90AB1, TUFM, HSP90AA1, GANAB, LMNB1, ANXA5, CAPZB, YWHAE, TPM4, ANXA2, FOS, RPS26, GNB1, PDGFRA, TOMM22, HSPA5, HSPD1, PCSK6, UGT2B7, HSPA8, NDUFS1* | 9.91E-05 | 0.0201096 |
|  | 10. | | GO:0044297~cell body | *TUBB, TCP1, GNB1, CCT8, PARK7* | 1.18E-04 | 0.0239088 |
| GO-MF | 1. | | GO:0005515~protein binding | *HSP90AB1, PDIA3, CROCC, PRDX3, VCL, FOS, GSN, AEN, CDK10, CCNA1, NDUFS1, EGR1, TYRO3, HSP90AA1, JUNB, WEE1, CHRAC1, ACTL7A, GNB1, PDGFRA, HSPB1, TOMM22, DST, TUFM, AHCY, LMNB1, NFKBIE, VIM, ELN, PTK7, ITGB4, KEAP1, VCPKMT, SFN, TPM4, TPM3, NXT2, ALCAM, RPS26, TUBB, COL6A2, CDA, HSPA5, HSPA8, HSPA9, FN1, ACTB, TCP1, COL4A2, PAIP2, NR4A1, ANXA5, YWHAE, ANXA4, PARK7, ANXA2, SYNE2, DUSP2, DUSP1, CCT8, MAP6, HSPD1, IGFBP2* | 9.88E-09 | 2.24E-06 |
|  | 2. | | GO:0051082~unfolded protein binding | *HSP90AB1, TCP1, HSP90AA1, CCT8, HSPD1, HSPA5, HSPA8, HSPA9* | 5.17E-07 | 1.17E-04 |
|  | 3. | | GO:0098641~cadherin binding involved in cell-cell adhesion | *HSP90AB1, RPS26, CCT8, HSPA5, SFN, YWHAE, CAPZB, PARK7, HSPA8, ANXA2, VCL* | 5.22E-07 | 1.19E-04 |
|  | 4. | | GO:0023026~MHC class II protein complex binding | *HSP90AB1, HSP90AA1, YWHAE, HSPA8* | 4.35E-05 | 0.0098181 |
|  | 5. | | GO:0005198~structural molecule activity | *LAMA2, COL18A1, TUBB, LMNB1, CROCC, LMNB2, VIM, VCL* | 1.03E-04 | 0.0231231 |
|  | 6. | | GO:0031625~ubiquitin protein ligase binding | *TUBB, TCP1, HSPD1, HSPA5, YWHAE, HSPA8, HSPA9, VCL* | 2.60E-04 | 0.0572296 |
|  | 7. | | GO:0004859~phospholipase inhibitor activity | *ANXA5, ANXA4, ANXA2* | 0.001016318 | 0.2061188 |
|  | 8. | | GO:0042802~identical protein binding | *ACTB, COL18A1, HSP90AA1, AHCY, VIM, HSPB1, PRDX3, SFN, ANXA4, PARK7, FN1* | 0.001531175 | 0.2937904 |
|  | 9. | | GO:0001948~glycoprotein binding | *HSP90AB1, HSP90AA1, VIM, HSPA5* | 0.002906708 | 0.4835534 |
|  | 10. | | GO:0044822~poly(A) RNA binding | *HSP90AB1, TUFM, TCP1, GANAB, HSP90AA1, PDIA3, YWHAE, ANXA2, RPS26, HSPB1, HSPD1, HSPA8, HSPA9* | 0.003504219 | 0.5492556 |
| KEGG | 1. | | hsa04151:PI3K-Akt signaling pathway | *LAMA2, HSP90AB1, COL4A2, HSP90AA1, GNB1, PDGFRA, COL6A2, ITGB4, NR4A1, YWHAE, COL5A2, FN1* | 1.47E-05 | 0.0017927 |
|  | 2. | | hsa04510:Focal adhesion | *ACTB, LAMA2, COL4A2, PDGFRA, COL6A2, ITGB4, COL5A2, FN1, VCL* | 6.50E-05 | 0.0078941 |
|  | 3. | | hsa04512:ECM-receptor interaction | *LAMA2, COL4A2, COL6A2, ITGB4, COL5A2, FN1* | 2.91E-04 | 0.0348944 |
|  | 4. | | hsa05146:Amoebiasis | *LAMA2, COL4A2, HSPB1, COL5A2, FN1, VCL* | 7.25E-04 | 0.0846538 |
|  | 5. | | hsa04974:Protein digestion and absorption | *COL18A1, COL4A2, ELN, COL6A2, COL5A2* | 0.002932616 | 0.30114 |
|  | 6. | | hsa05200:Pathways in cancer | *LAMA2, HSP90AB1, FOS, COL4A2, HSP90AA1, GNB1, PDGFRA, FN1, TPM3* | 0.004655639 | 0.4340867 |
|  | 7. | | hsa04141:Protein processing in endoplasmic reticulum | *HSP90AB1, HSP90AA1, GANAB, PDIA3, HSPA5, HSPA8* | 0.005620189 | 0.4972172 |
|  | 8. | | hsa04010:MAPK signaling pathway | *FOS, DUSP2, DUSP1, PDGFRA, HSPB1, NR4A1, HSPA8* | 0.007064752 | 0.578931 |
|  | 9. | | hsa04810:Regulation of actin cytoskeleton | *ACTB, GSN, PDGFRA, ITGB4, FN1, VCL* | 0.013688172 | 0.8139038 |
|  | 10. | | hsa04612:Antigen processing and presentation | *HSP90AB1, HSP90AA1, PDIA3, HSPA8* | 0.014835857 | 0.838546 |
| **ES-phase gene list:** | | | | | | |
| GO-BP | 1. | | GO:0071294~cellular response to zinc ion | *MT1L, MT1X, MT1G, MT1F* | 6.41E-05 | 0.044236037 |
|  | 2. | | GO:0045926~negative regulation of growth | *MT1L, MT1X, MT1G, MT1F* | 6.41E-05 | 0.044236037 |
|  | 3. | | GO:0042493~response to drug | *CDK1, TYMS, FOS, APOD, SCN11A, FOSB, PTEN, CTNNB1* | 2.69E-04 | 0.172852943 |
|  | 4. | | GO:0009636~response to toxic substance | *CDK1, TYMS, FOS, TRPM6, EPHX1* | 4.35E-04 | 0.264339494 |
|  | 5. | | GO:0007565~female pregnancy | *NAMPT, FOS, OVGP1, UCP2, FOSB* | 5.18E-04 | 0.306218805 |
|  | 6. | | GO:0071276~cellular response to cadmium ion | *MT1X, MT1G, MT1F* | 0.002237333 | 0.794297984 |
|  | 7. | | GO:0000083~regulation of transcription involved in G1/S transition of mitotic cell cycle | *CDK1, TYMS, RRM2* | 0.004095469 | 0.944830112 |
|  | 8. | | GO:0007568~aging | *TYMS, FOS, APOD, UCP2, PTEN* | 0.004981914 | 0.970578278 |
|  | 9. | | GO:0060070~canonical Wnt signaling pathway | *MED12, FZD2, PTEN, CTNNB1* | 0.005022905 | 0.97142169 |
|  | 10. | | GO:0046511~sphinganine biosynthetic process | *SPTLC1, SPTLC2* | 0.008320173 | 0.997256759 |
| GO-CC | 1. | | GO:0048471~perinuclear region of cytoplasm | *CAPN6, MT1L, OLFM4, SEC24A, SET, APOD, MT1X, MT1G, CTNNB1, MT1F* | 7.33E-04 | 0.107501251 |
|  | 2. | | GO:0005737~cytoplasm | *NAMPT, KCNAB1, NAA38, PTEN, CTNNB1, TYMS, PTK2, MCTP2, TMEM50A, SET, BDH2, TOP2A, MT1G, ASPM, MT1F, EGR1, CFLAR, CDK1, OVGP1, MT1L, NUB1, SAP18, WNK2, FZD2, MT1X, NMT2, RNF7, RRM2, SFRP4, TCF12, PARVA, TOB1* | 0.006243103 | 0.621187612 |
|  | 3. | | GO:0005634~nucleus | *U2SURP, ZNF827, NAA38, PTEN, CTNNB1, APOBEC3B, FOS, TYMS, PTK2, SET, USP36, TOP2A, MT1G, ASPM, MT1F, EGR1, CDK1, MT1L, NUB1, MED12, NUF2, PPP1R10, FAM76B, FOSB, PBK, MT1X, RNF7, RRM2, SFRP4, TCF12, PARVA, TOB1* | 0.010949106 | 0.818494744 |
|  | 4. | | GO:0017059~serine C-palmitoyltransferase complex | *SPTLC1, SPTLC2* | 0.019600829 | 0.953499784 |
|  | 5. | | GO:0005789~endoplasmic reticulum membrane | *TMED4, SPTLC1, SEC24A, SPTLC2, EPHX1, KDSR, ALG13, SDF2, CYP4B1* | 0.020073572 | 0.956849294 |
|  | 6. | | GO:0005743~mitochondrial inner membrane | *TYMS, MRPL51, MRPS18C, UCP2, NDUFA1, DNAJC19* | 0.030241633 | 0.991432621 |
|  | 7. | | GO:0005739~mitochondrion | *CDK1, MCCC2, TYMS, OLFM4, MRPS18C, SPTLC2, ELN, BDH2, PTEN, NDUFA1, DNAJC19* | 0.035788922 | 0.996478902 |
|  | 8. | | GO:0005654~nucleoplasm | *EGR1, NAMPT, CDK1, U2SURP, MED12, SAP18, PPP1R10, ANLN, PTEN, CTNNB1, FOS, TYMS, RNF7, MCTP2, SET, RRM2, TOP2A, KIF20A* | 0.041049992 | 0.998492092 |
|  | 9. | | GO:0005829~cytosol | *NAMPT, CFLAR, CDK1, OVGP1, SEC24A, KCNAB1, NUF2, WNK2, PTEN, CTNNB1, NMT2, TYMS, FOS, MCCC2, PTK2, SET, RRM2, BDH2, PARVA, SPTAN1* | 0.053810208 | 0.999810927 |
|  | 10. | | GO:0005615~extracellular space | *NAMPT, CPM, OLFM4, APOD, CXCL13, CST4, SFRP4, KDSR, CST1, SDF2* | 0.082688513 | 0.999998451 |
| GO-MF | 1. | | GO:0008144~drug binding | *NAMPT, TYMS, TOP2A, CYP4B1* | 0.00402393 | 0.549926948 |
|  | 2. | | GO:0046332~SMAD binding | *TCF12, CTNNB1, TOB1* | 0.014092018 | 0.939798992 |
|  | 3. | | GO:0004758~serine C-palmitoyltransferase activity | *SPTLC1, SPTLC2* | 0.020855856 | 0.98459602 |
|  | 4. | | GO:0002020~protease binding | *CFLAR, CST4, CST1* | 0.067499605 | 0.999999022 |
|  | 5. | | GO:0001077~transcriptional activator activity, RNA polymerase II core promoter proximal region sequence-specific binding | *EGR1, FOS, FOSB, TCF12* | 0.077251531 | 0.999999878 |
|  | 6. | | GO:0042813~Wnt-activated receptor activity | *SFRP4, FZD2* | 0.088608789 | 0.999999989 |
|  | 7. | | GO:0000982~transcription factor activity, RNA polymerase II core promoter proximal region sequence-specific binding | *FOS, FOSB* | 0.092447022 | 0.999999995 |
|  | 8. | | GO:0045296~cadherin binding | *OLFM4, CTNNB1* | 0.092447022 | 0.999999995 |
| KEGG | 1. | | hsa04978:Mineral absorption | *TRPM6, MT1X, MT1G, MT1F* | 0.001630151 | 0.153301924 |
|  | 2. | | hsa00600:Sphingolipid metabolism | *SPTLC1, SPTLC2, KDSR* | 0.026145327 | 0.932949265 |
|  | 3. | | hsa04115:p53 signaling pathway | *CDK1, RRM2, PTEN* | 0.050022763 | 0.994669766 |
|  | 4. | | hsa04510:Focal adhesion | *PTK2, PTEN, CTNNB1, PARVA* | 0.097913088 | 0.999972752 |
| **MS-phase gene list:** | | | | | | |
| GO-BP | 1. | | GO:0035767~endothelial cell chemotaxis | *CORO1B, EGR3, VEGFA, NR4A1* | 3.85E-05 | 0.043268516 |
|  | 2. | | GO:0006954~inflammatory response | *CCL3, IL6, AIMP1, S100A8, CXCL2, ACKR1, CCL8, FOS, CCL3L1, CCL3L3, LTB4R2, PTAFR, SCG2* | 4.50E-05 | 0.050312962 |
|  | 3. | | GO:0070098~chemokine-mediated signaling pathway | *CCL3, CCL3L1, CCL3L3, CXCL2, ACKR1, CCL8* | 2.39E-04 | 0.240009153 |
|  | 4. | | GO:0043066~negative regulation of apoptotic process | *IL6, EGR3, SOCS3, VHL, ANXA5, FMN2, EDNRB, HSP90B1, SON, KRT18, VEGFA, MAPK8, DNAJC3* | 2.51E-04 | 0.250072259 |
|  | 5. | | GO:0050729~positive regulation of inflammatory response | *CCL3, S100A8, CCL3L1, AGT, CCL3L3, CCL8* | 2.72E-04 | 0.268515007 |
|  | 6. | | GO:0002548~monocyte chemotaxis | *IL6, CCL3, CCL3L1, CCL3L3, CCL8* | 3.24E-04 | 0.310624374 |
|  | 7. | | GO:0035914~skeletal muscle cell differentiation | *EGR1, FOS, ATF3, EGR2, NR4A1* | 5.88E-04 | 0.490811473 |
|  | 8. | | GO:0045944~positive regulation of transcription from RNA polymerase II promoter | *EGR1, IL6, EGR2, MET, TGFB3, NR4A1, FOSB, NR4A3, YBX1, JUNB, FOS, DDX17, ATF3, PAX8, VEGFA, THRAP3, NFAT5, PER1, PITX1* | 6.69E-04 | 0.535926315 |
|  | 9. | | GO:0048247~lymphocyte chemotaxis | *CCL3, CCL3L1, CCL3L3, CCL8* | 0.001347142 | 0.787233832 |
|  | 10. | | GO:0071356~cellular response to tumor necrosis factor | *ZFP36, IL6, CCL3, CCL3L1, CCL3L3, CCL8* | 0.001761131 | 0.867815987 |
| GO-CC | 1. | | GO:0005615~extracellular space | *IFNA21, CCL3, S100A8, CXCL2, TGFB3, CCL8, C1QTNF6, GSN, CCL3L1, AGT, CCL3L3, CCBE1, SEMA3C, COL12A1, AMY2A, PCSK5, SCG2, CRISP3, IL6, AIMP1, ANXA2, CTSW, CST7, PRDX6, SERPINB8, KRIT1, VEGFA, AOC1, AMY1A* | 3.68E-07 | 7.10E-05 |
|  | 2. | | GO:0070062~extracellular exosome | *SLC44A2, S100A8, ENPP3, VIM, RNH1, LUZP1, YBX1, PDHB, KRT5, GSN, AGT, COL12A1, SEMA3C, DNAJC3, AMY2B, AMY2A, SLC1A1, CRISP3, BST2, ABCB11, MUC7, MYL12A, ANXA5, TAF6L, YWHAE, ANXA2, C1QA, CORO1B, HSP90B1, LAMA3, KRT18, PRDX6, SERPINB8, LCK, CCT8, EIF4A1, THRAP3, MAP4, PCYOX1, CA1, DST, AOC1, AMY1A* | 2.20E-06 | 4.25E-04 |
|  | 3. | | GO:0005576~extracellular region | *IFNA21, CRISP3, IL6, CCL3, ADGRF1, S100A8, PLEK, GZMA, MET, CXCL2, TGFB3, CTSW, C1QA, HSP90B1, LAMA3, GSN, CCL3L1, AGT, CCL3L3, VEGFA, GALP, CDA, COL12A1, AMY2A* | 0.001341942 | 0.228307251 |
|  | 4. | | GO:0016020~membrane | *CDK5R1, LUZP1, PRRC2C, FOS, DDX17, KRT5, DNAJC3, SLC1A1, PLEK, BST2, AIMP1, ABCB11, IMMT, VHL, ANXA5, YWHAE, ANXA2, CTSW, HSP90B1, PRDX6, VEGFA, EIF4A1, LTB4R2, ABCC3, CELF1, PTAFR, PLEKHA2* | 0.00859722 | 0.811081026 |
|  | 5. | | GO:0005882~intermediate filament | *KRT18, KRT5, VIM, DST, KRTAP19-2* | 0.009464366 | 0.840437818 |
|  | 6. | | GO:0005667~transcription factor complex | *FOS, HOXA9, NR4A1, NR4A3, JUNB, PITX1* | 0.013673983 | 0.929861838 |
|  | 7. | | GO:0030141~secretory granule | *VEGFA, TGFB3, PCSK5, SCG2* | 0.016905525 | 0.962770753 |
|  | 8. | | GO:0031012~extracellular matrix | *HSP90B1, VIM, POMZP3, EIF4A1, TGFB3, COL12A1, ANXA2* | 0.021565961 | 0.985120451 |
|  | 9. | | GO:0005925~focal adhesion | *CORO1B, HSP90B1, LPP, GSN, VIM, ANXA5, DST, YWHAE* | 0.024715532 | 0.99201376 |
|  | 10. | | GO:0005581~collagen trimer | *C1QA, C1QTNF6, CCBE1, COL12A1* | 0.029811934 | 0.997094679 |
| GO-MF | 1. | | GO:0001077~transcriptional activator activity, RNA polymerase II core promoter proximal region sequence-specific binding | *EGR1, FOS, EGR2, PAX8, NFAT5, NR4A1, NR4A3, FOSB, YBX1, JUNB, PITX1* | 1.55E-05 | 0.004998397 |
|  | 2. | | GO:0000978~RNA polymerase II core promoter proximal region sequence-specific DNA binding | *FOS, ATF3, RBBP4, EGR2, PAX8, NFAT5, PER1, NR4A3, FOSB, YBX1, JUNB, PITX1* | 1.02E-04 | 0.032382994 |
|  | 3. | | GO:0008009~chemokine activity | *CCL3, CCL3L1, CCL3L3, CXCL2, CCL8* | 5.60E-04 | 0.165509075 |
|  | 4. | | GO:0005515~protein binding | *CRABP1, S100A8, RSRP1, TGFB3, YBX1, PDHB, SLA, SHB, EDNRB, FOS, DDX17, MALL, GSN, PAX8, CCBE1, SAP30L, DLG5, SLC1A1, PITX1, TUBA1C, EGR1, ZFP36, TRPM6, RBBP4, EGR2, BST2, GZMA, SOCS3, TAF6L, JUNB, C1QA, KRT18, PRDX6, CST7, SERPINB8, EIF4A2, KRIT1, VEGFA, LCK, EIF4A1, RIN1, CELF1, MAPK8, CA1, DST, AMY1A, SCGB2A2, CDK5R1, CCL3, ALPI, VIM, CXCL2, RNH1, TRIM15, C1QTNF6, KRT5, AGT, NFAT5, CDA, HOXA9, DDIT4L, PCSK5, SCG2, IL6, AIMP1, PLEK, VHL, LPP, IMMT, HPCAL4, MET, AFF4, SMG1, NR4A1, MUC7, MYL12A, NR4A3, ANXA5, YWHAE, ANXA2, CORO1B, HSP90B1, SON, ATF3, THRAP3, CCT8, MAP4, PLEKHA2* | 5.80E-04 | 0.170851982 |
|  | 5. | | GO:0004556~alpha-amylase activity | *AMY2B, AMY2A, AMY1A* | 5.89E-04 | 0.173174591 |
|  | 6. | | GO:0097110~scaffold protein binding | *CASP5, KRT18, KRT5, VIM* | 0.006123468 | 0.862476549 |
|  | 7. | | GO:0005509~calcium ion binding | *S100A3, CDK5R1, HSP90B1, S100A8, GSN, HPCAL4, CCBE1, MYL12A, ANXA5, AOC1, DST, AMY2A, ANXA2* | 0.009926877 | 0.960140807 |
|  | 8. | | GO:0005125~cytokine activity | *IFNA21, IL6, AIMP1, VEGFA, TGFB3, SCG2* | 0.012071529 | 0.980214959 |
|  | 9. | | GO:0048020~CCR chemokine receptor binding | *CCL3L1, CCL3L3, CCL8* | 0.013591213 | 0.987966577 |
|  | 10. | | GO:0000982~transcription factor activity, RNA polymerase II core promoter proximal region sequence-specific binding | *FOS, ATF3, FOSB* | 0.013591213 | 0.987966577 |
| KEGG | 1. | | hsa05142:Chagas disease (American trypanosomiasis) | *C1QA, FOS, IL6, CCL3, CCL3L1, CCL3L3, TGFB3, MAPK8* | 2.05E-04 | 0.033430146 |
|  | 2. | | hsa05132:Salmonella infection | *FOS, IL6, CCL3, CCL3L1, CCL3L3, CXCL2, MAPK8* | 4.05E-04 | 0.06510747 |
|  | 3. | | hsa05323:Rheumatoid arthritis | *FOS, IL6, CCL3, CCL3L1, VEGFA, CCL3L3, TGFB3* | 5.56E-04 | 0.08813421 |
|  | 4. | | hsa04620:Toll-like receptor signaling pathway | *IFNA21, FOS, IL6, CCL3, CCL3L1, CCL3L3, MAPK8* | 0.001478947 | 0.21783295 |
|  | 5. | | hsa00500:Starch and sucrose metabolism | *ENPP3, AMY2B, AMY2A, AMY1A* | 0.006654704 | 0.669904363 |
|  | 6. | | hsa05161:Hepatitis B | *IFNA21, FOS, EGR3, IL6, EGR2, TGFB3, MAPK8* | 0.007015806 | 0.689238136 |
|  | 7. | | hsa04668:TNF signaling pathway | *FOS, IL6, SOCS3, CXCL2, MAPK8, JUNB* | 0.008331705 | 0.750639809 |
|  | 8. | | hsa05200:Pathways in cancer | *EDNRB, FOS, HSP90B1, IL6, LAMA3, VHL, PAX8, VEGFA, MET, TGFB3, MAPK8* | 0.016395912 | 0.935705483 |
|  | 9. | | hsa04380:Osteoclast differentiation | *FOS, SOCS3, LCK, MAPK8, FOSB, JUNB* | 0.0187632 | 0.956902172 |
|  | 10. | | hsa05144:Malaria | *IL6, MET, TGFB3, ACKR1* | 0.019649188 | 0.962903652 |
| **LS-phase gene list:** | | | | | | |
| GO-BP | 1. | | GO:0030198~extracellular matrix organization | *HPSE2, RXFP1, HPSE, FN1, ITGA2B* | 1.88E-04 | 0.042841796 |
|  | 2. | | GO:0030200~heparan sulfate proteoglycan catabolic process | *HPSE2, HPSE* | 0.00594246 | 0.75060657 |
|  | 3. | | GO:0007409~axonogenesis | *OGN, BAIAP2, LRRN1* | 0.009267784 | 0.885760969 |
|  | 4. | | GO:0002576~platelet degranulation | *ORM1, FN1, ITGA2B* | 0.010196366 | 0.908182126 |
|  | 5. | | GO:0008284~positive regulation of cell proliferation | *HPSE2, IL6ST, FN1, GLI1* | 0.031055968 | 0.999357911 |
|  | 6. | | GO:0009617~response to bacterium | *BAIAP2, DEFB1* | 0.033709241 | 0.999661059 |
|  | 7. | | GO:0006027~glycosaminoglycan catabolic process | *HPSE2, HPSE* | 0.03802483 | 0.999880552 |
|  | 8. | | GO:0010575~positive regulation of vascular endothelial growth factor production | *HPSE, IL6ST* | 0.039459244 | 0.999915631 |
|  | 9. | | GO:0006953~acute-phase response | *ORM1, FN1* | 0.056512843 | 0.999998701 |
|  | 10. | | GO:0048146~positive regulation of fibroblast proliferation | *CDK6, FN1* | 0.077421366 | 0.999999993 |
| GO-CC | 1. | | GO:0005615~extracellular space | *SCGB2A1, OGN, ORM1, CPE, IL6ST, PTPRR, STC1, DEFB1, FN1* | 5.43E-04 | 0.039916763 |
|  | 2. | | GO:0070062~extracellular exosome | *OGN, ORM1, SNCG, CYP2J2, S100P, CPE, IL6ST, BAIAP2, KMO, DEFB1, FN1, ITGA2B* | 0.00135932 | 0.09698699 |
|  | 3. | | GO:0005578~proteinaceous extracellular matrix | *OGN, HPSE2, HPSE, FN1* | 0.007086146 | 0.413365241 |
|  | 4. | | GO:0043025~neuronal cell body | *SNCG, CPE, IL6ST, BAIAP2* | 0.011006323 | 0.563972744 |
|  | 5. | | GO:0072562~blood microparticle | *ORM1, FN1, ITGA2B* | 0.021163496 | 0.79896957 |
|  | 6. | | GO:0031093~platelet alpha granule lumen | *ORM1, FN1* | 0.078421689 | 0.997812649 |
|  | 7. | | GO:0005576~extracellular region | *OGN, ORM1, SCGB1D4, IL6ST, DEFB1, FN1* | 0.084445491 | 0.998662433 |
| GO-MF | 1. | | GO:0030305~heparanase activity | *HPSE2, HPSE* | 0.002959804 | 0.247656696 |
|  | 2. | | GO:0004566~beta-glucuronidase activity | *HPSE2, HPSE* | 0.005911197 | 0.433998652 |
|  | 3. | | GO:0005515~protein binding | *SNCG, S100P, RXFP1, IL6ST, BAIAP2, PTPRR, FAM118A, CDK6, PCDH17, GLI1, OGN, ORM1, HPSE, BHLHE40, DEFB1, SCGB2A2, FN1, JAKMIP1, RDM1, ITGA2B* | 0.012506562 | 0.701265773 |
|  | 4. | | GO:0016798~hydrolase activity, acting on glycosyl bonds | *HPSE2, HPSE* | 0.01471514 | 0.759046234 |
|  | 5. | | GO:0043395~heparan sulfate proteoglycan binding | *HPSE2, HPSE* | 0.026337358 | 0.922871182 |
|  | 6. | | GO:0003705~transcription factor activity, RNA polymerase II distal enhancer sequence-specific binding | *BHLHE40, GLI1* | 0.093355104 | 0.99991797 |
|  | 7. | | GO:0042802~identical protein binding | *BAIAP2, FAM118A, FN1, ITGA2B* | 0.09712198 | 0.999944996 |
| KEGG | 1. | | hsa05222:Small cell lung cancer | *CDK6, FN1, ITGA2B* | 0.010774277 | 0.38582372 |
|  | 2. | | hsa05200:Pathways in cancer | *CDK6, FN1, ITGA2B, GLI1* | 0.034481475 | 0.793829039 |
|  | 3. | | hsa00531:Glycosaminoglycan degradation | *HPSE2, HPSE* | 0.035347652 | 0.801989984 |
|  | 4. | | hsa05205:Proteoglycans in cancer | *HPSE2, HPSE, FN1* | 0.053150816 | 0.914367935 |
|  | 5. | | hsa04810:Regulation of actin cytoskeleton | *BAIAP2, FN1, ITGA2B* | 0.057994781 | 0.932017222 |
| **N/S-phase gene list:** | | | | | | |
| GO-BP: | | 1. | No output | */* | / | / |
| GO-CC: | | 1. | No output | */* | / | / |
| GO-MF: | | 1. | No output | */* | / | / |
| KEGG | | 1. | No output | */* | / | / |
