# Supplementary material for: Molecular signature of eutopic endometrium in endometriosis based on the multi-omics integrative synthesis
Source: J Assist Reprod Genet. 2020 May 30;37(7):1593–611. doi: 10.1007/s10815-020-01833-3 (PMC7376782; doi:10.1007/s10815-020-01833-3)
Supplement: Supplementary file 5 — (DOCX 141 kb). [file 10815_2020_1833_MOESM5_ESM.docx]

**Supplementary file S5:** Repeated genes in the catalogue with associated phase of the menstrual cycle, omics level and source reference.

| **Gene ID from NCBI** | **Gene symbol according to the HGNC nomenclature** | **Phase of the menstrual cycle** | **Comments** | **Omics level** | **Source reference** |
| --- | --- | --- | --- | --- | --- |
| 60 | *ACTB* | (P) | Reported up- and  down-regulated (multiple protein isoforms) | Proteomics | [30] |
| 60 | *ACTB* | (P) |  | Proteomics | [30] |
| 60 | *ACTB* | (P) | / | Proteomics | [44] |
| 60 | *ACTB* | (S) | Reported up- and  down-regulated (multiple protein isoforms) | Proteomics | [30] |
| 60 | *ACTB* | (S) |  | Proteomics | [30] |
| 60 | *ACTB* | (S) | / | Proteomics | [44] |
| 27125 | *AFF4* | (MS) | / | Transcriptomics | [37] |
| 27125 | *AFF4* | (P) | / | Transcriptomics | [37] |
| 280 | *AMY2B* | (MS) | / | Transcriptomics | [37] |
| 280 | *AMY2B* | (S) | / | Transcriptomics | [34] |
| 54443 | *ANLN* | (ES) | / | Transcriptomics | [37] |
| 54443 | *ANLN* | (ES) | / | Transcriptomic | [25] |
| 302 | *ANXA2* | (MS) | / | Proteomics | [46] |
| 302 | *ANXA2* | (S) | / | Proteomics | [44] |
| 307 | *ANXA4* | (P) | / | Proteomics | [30] |
| 307 | *ANXA4* | (P) | / | Transcriptomics | [34] |
| 307 | *ANXA4* | (S) | / | Proteomics | [30] |
| 308 | *ANXA5* | (MS) | / | Proteomics | [46] |
| 308 | *ANXA5* | (P) | / | Proteomics | [30] |
| 308 | *ANXA5* | (S) | / | Proteomics | [30] |
| 506 | *ATP5F1B* | (P) | / | Proteomics | [30] |
| 506 | *ATP5F1B* | (S) | / | Proteomics | [30] |
| 92747 | *BPIFB1* | (P) | / | Transcriptomic | [25] |
| 92747 | *BPIFB1* | (P) | / | Transcriptomics | [38] |
| 832 | *CAPZB* | (P) | / | Proteomics | [30] |
| 832 | *CAPZB* | (S) | / | Proteomics | [30] |
| 3491 | *CCN1* | (MS) | / | Transcriptomics | [37] |
| 3491 | *CCN1* | (MS) | / | Transcriptomics | [29] |
| 3491 | *CCN1* | (P) | / | Transcriptomics | [37] |
| 3491 | *CCN1* | (S) | / | Transcriptomics | [34] |
| 10694 | *CCT8* | (MS) | / | Proteomics | [46] |
| 10694 | *CCT8* | (P) | / | Proteomics | [30] |
| 10694 | *CCT8* | (S) | / | Proteomics | [30] |
| 978 | *CDA* | (MS) | / | Transcriptomics | [37] |
| 978 | *CDA* | (S) | / | Proteomics | [48] |
| 8558 | *CDK10* | (P) | / | Transcriptomics | [34] |
| 8558 | *CDK10* | (S) | / | Proteomics | [47] |
| 1292 | *COL6A2* | (P) | / | Proteomics | [30] |
| 1292 | *COL6A2* | (S) | / | Proteomics | [30] |
| 10321 | *CRISP3* | (MS) | / | Transcriptomic | [25] |
| 10321 | *CRISP3* | (MS) | / | Transcriptomics | [37] |
| 10521 | *DDX17* | (MS) | / | Transcriptomics | [37] |
| 10521 | *DDX17* | (P) | / | Transcriptomic | [25] |
| 667 | *DST* | (MS) | / | Transcriptomics | [37] |
| 667 | *DST* | (S) | / | Proteomics | [47] |
| 1958 | *EGR1* | (ES) | / | Transcriptomics | [37] |
| 1958 | *EGR1* | (MS) | / | Transcriptomics | [37] |
| 1958 | *EGR1* | (MS) | / | Transcriptomics | [29] |
| 1958 | *EGR1* | (P) | / | Transcriptomics | [37] |
| 1958 | *EGR1* | (S) | / | Transcriptomics | [34] |
| 10209 | *EIF1* | (MS) | / | Transcriptomics | [37] |
| 10209 | *EIF1* | (P) | / | Transcriptomic | [25] |
| 2006 | *ELN* | (ES) | / | Transcriptomics | [37] |
| 2006 | *ELN* | (S) | / | Transcriptomics | [34] |
| 2052 | *EPHX1* | (ES) | / | Transcriptomics | [37] |
| 2052 | *EPHX1* | (P) | / | Transcriptomics | [34] |
| 2052 | *EPHX1* | (P) | / | Transcriptomics | [37] |
| 55007 | *FAM118A* | (LS) | / | Transcriptomics | [75] |
| 55007 | *FAM118A* | (P) | / | Transcriptomics | [38] |
| 143684 | *FAM76B* | (ES) | / | Transcriptomics | [37] |
| 143684 | *FAM76B* | (P) | / | Transcriptomics | [37] |
| 2335 | *FN1* | (LS) | / | Transcriptomics | [36] |
| 2335 | *FN1* | (S) | / | Transcriptomics | [34] |
| 2353 | *FOS* | (ES) | / | Transcriptomics | [37] |
| 2353 | *FOS* | (MS) | / | Transcriptomics | [37] |
| 2353 | *FOS* | (MS) | / | Transcriptomics | [29] |
| 2353 | *FOS* | (P) | / | Transcriptomics | [37] |
| 2353 | *FOS* | (S) | / | Transcriptomics | [34] |
| 2354 | *FOSB* | (ES) | / | Transcriptomics | [37] |
| 2354 | *FOSB* | (MS) | / | Transcriptomics | [37] |
| 2354 | *FOSB* | (P) | / | Transcriptomics | [37] |
| 2354 | *FOSB* | (MS) | / | Transcriptomics | [29] |
| 23193 | *GANAB* | (P) | / | Proteomics | [30] |
| 23193 | *GANAB* | (S) | / | Proteomics | [30] |
| 2782 | *GNB1* | (P) | / | Proteomics | [30] |
| 2782 | *GNB1* | (S) | / | Proteomics | [30] |
| 2934 | *GSN* | (MS) | / | Proteomics | [46] |
| 2934 | *GSN* | (S) | / | Proteomics | [30] |
| 3326 | *HSP90AB1* | (P) | / | Transcriptomics | [37] |
| 3326 | *HSP90AB1* | (S) | / | Proteomics | [47] |
| 3326 | *HSP90AB1* | (S) | / | Proteomics | [44] |
| 3309 | *HSPA5* | (P) | / | Proteomics | [30] |
| 3309 | *HSPA5* | (S) | / | Proteomics | [30] |
| 3313 | *HSPA9* | (P) | / | Proteomics | [30] |
| 3313 | *HSPA9* | (S) | / | Proteomics | [30] |
| 3315 | *HSPB1* | (P) | / | Proteomics | [30] |
| 3315 | *HSPB1* | (S) | / | Proteomics | [30] |
| 3329 | *HSPD1* | (P) | / | Proteomics | [30] |
| 3329 | *HSPD1* | (S) | Reported up- and  down-regulated | Proteomics | [30] |
| 3329 | *HSPD1* | (S) |  | Proteomics | [30] |
| 10989 | *IMMT* | (MS) | / | Proteomics | [46] |
| 10989 | *IMMT* | (P) | / | Proteomics | [30] |
| 3726 | *JUNB* | (MS) | / | Transcriptomics | [37] |
| 3726 | *JUNB* | (P) | / | Transcriptomics | [37] |
| 3726 | *JUNB* | (S) | / | Transcriptomics | [34] |
| 105370479 | *LINC02303* | (LS) | Two transcripts of the same gene: ENST00000554711 and ENST00000554810 | ncRNomics | [75] |
| 105370479 | *LINC02303* | (LS) |  | ncRNomics | [75] |
| 84823 | *LMNB2* | (P) | / | Proteomics | [30] |
| 84823 | *LMNB2* | (S) | / | Proteomics | [30] |
| 164832 | *LONRF2* | (MS) | / | Transcriptomics | [37] |
| 164832 | *LONRF2* | (P) | / | Transcriptomics | [37] |
| 406929 | *MIR138-1* | (MS) | / | ncRNomics | [29] |
| 406929 | *MIR138-1* | N/S | / | ncRNomics | [39] |
| 406930 | *MIR138-2* | (MS) | / | ncRNomics | [29] |
| 406930 | *MIR138-2* | N/S | / | ncRNomics | [39] |
| 407042 | *MIR34C* | (ES) | Dysregulated both, miRNA-5p and miRNA-3p strands. | ncRNomics | [40] |
| 407042 | *MIR34C* | (ES) |  | ncRNomics | [40] |
| 100126317 | *MIR374B* | (MS) | / | ncRNomics | [29] |
| 100126317 | *MIR374B* | (P) | / | ncRNomics | [43] |
| 4494 | *MT1F* | (ES) | Locus name: *metallothionein 1F (functional)* and locus name: *MT1F: Metallothionein 1F (functional)* resulted in the same HGNC symbol. | Transcriptomic | [25] |
| 4494 | *MT1F* | (ES) |  | Transcriptomic | [25] |
| 727897 | *MUC5B* | (P) | / | Transcriptomics | [34] |
| 727897 | *MUC5B* | (P) | / | Transcriptomics | [37] |
| 727897 | *MUC5B* | (P) | / | Transcriptomics | [38] |
| 4719 | *NDUFS1* | (P) | / | Proteomics | [30] |
| 4719 | *NDUFS1* | (S) | / | Proteomics | [30] |
| 10725 | *NFAT5* | (MS) | / | Transcriptomics | [37] |
| 10725 | *NFAT5* | (P) | / | Transcriptomics | [37] |
| 3164 | *NR4A1* | (MS) | / | Transcriptomics | [29] |
| 3164 | *NR4A1* | (S) | / | Transcriptomics | [34] |
| 5016 | *OVGP1* | (ES) | / | Transcriptomics | [37] |
| 5016 | *OVGP1* | (P) | / | Transcriptomic | [25] |
| 11315 | *PARK7* | (P) | / | Proteomics | [30] |
| 11315 | *PARK7* | (S) | / | Proteomics | [30] |
| 5125 | *PCSK5* | (MS) | / | Transcriptomics | [37] |
| 5125 | *PCSK5* | (P) | / | Transcriptomics | [37] |
| 51449 | *PCYOX1* | (MS) | / | Transcriptomics | [37] |
| 51449 | *PCYOX1* | (P) | / | Transcriptomics | [37] |
| 2923 | *PDIA3* | (P) | / | Proteomics | [30] |
| 2923 | *PDIA3* | (S) | / | Proteomics | [30] |
| 22932 | *POMZP3* | (MS) | Locus name: *POM (POM121 homolog, rat)* *and ZP3 fusion* reported as down-regulated twice | Transcriptomic | [25] |
| 22932 | *POMZP3* | (MS) |  | Transcriptomic | [25] |
| 7001 | *PRDX2* | (P) | / | Proteomics | [30] |
| 7001 | *PRDX2* | (P) | / | Proteomics | [44] |
| 7001 | *PRDX2* | (S) | / | Proteomics | [30] |
| 10935 | *PRDX3* | (P) | / | Proteomics | [30] |
| 10935 | *PRDX3* | (S) | / | Proteomics | [30] |
| 6241 | *RRM2* | (ES) | The same locus ribonucleotide reductase M2 polypeptide reported as up-regulated twice. | Transcriptomic | [25] |
| 6241 | *RRM2* | (ES) |  | Transcriptomic | [25] |
| 6279 | *S100A8* | (MS) | / | Transcriptomic | [25] |
| 6279 | *S100A8* | (P) | / | Transcriptomic | [25] |
| 4250 | *SCGB2A2* | (ES) | / | Transcriptomic | [25] |
| 4250 | *SCGB2A2* | (LS) | / | Transcriptomics | [75] |
| 4250 | *SCGB2A2* | (MS) | / | Transcriptomic | [25] |
| 780851 | *SNORD3A* | (P) | Two transcripts of the gene, ENST00000365494.1 and ENST00000584923.1 | ncRNomics | [38] |
| 780851 | *SNORD3A* | (P) |  | ncRNomics | [38] |
| 6709 | *SPTAN1* | (ES) | Down-regulated expression when E *vs*. non-E, but other uterine/pelvic pathologies.  Up-regulated expression when compared E *vs*. healthy non-E | Transcriptomics | [37] |
| 6709 | *SPTAN1* | (ES) |  | Transcriptomics | [37] |
| 6950 | *TCP1* | (P) | / | Proteomics | [30] |
| 6950 | *TCP1* | (S) | / | Proteomics | [30] |
| 7153 | *TOP2A* | (ES) | / | Transcriptomic | [25] |
| 7153 | *TOP2A* | (P) | / | Transcriptomics | [37] |
| 7171 | *TPM4* | (P) | / | Proteomics | [30] |
| 7171 | *TPM4* | (S) | / | Proteomics | [30] |
| 140803 | *TRPM6* | (ES) | / | Transcriptomic | [25] |
| 140803 | *TRPM6* | (MS) | / | Transcriptomics | [37] |
| 140803 | *TRPM6* | (MS) | / | Transcriptomic | [25] |
| 203068 | *TUBB* | (P) | / | Proteomics | [30] |
| 203068 | *TUBB* | (S) | / | Proteomics | [30] |
| 1462 | *VCAN* | (P) | The same locus *versican* reported as down-regulated twice. | Transcriptomics | [37] |
| 1462 | *VCAN* | (P) |  | Transcriptomics | [37] |
| 7414 | *VCL* | (P) | / | Proteomics | [30] |
| 7414 | *VCL* | (S) | / | Proteomics | [30] |
| 7431 | *VIM* | (MS) | Reported up- and down-regulated (multiple protein isoforms) | Proteomics | [46] |
| 7431 | *VIM* | (MS) |  | Proteomics | [46] |
| 7431 | *VIM* | (P) | Reported up- and down-regulated (multiple protein isoforms) | Proteomics | [30] |
| 7431 | *VIM* | (P) |  | Proteomics | [30] |
| 7431 | *VIM* | (P) | / | Proteomics | [44] |
| 7431 | *VIM* | (S) | / | Proteomics | [30] |
| 7431 | *VIM* | (S) | / | Proteomics | [47] |
| 7431 | *VIM* | (S) | / | Proteomics | [44] |
| 7531 | *YWHAE* | (MS) | / | Proteomics | [46] |
| 7531 | *YWHAE* | (S) | / | Proteomics | [30] |
| 7538 | *ZFP36* | (MS) | / | Transcriptomics | [37] |
| 7538 | *ZFP36* | (P) | / | Transcriptomics | [37] |

Legend: / = the locus not repeated in the same phase of the menstrual cycle within the same study. P = proliferative phase. S = secretory phase. ES = early-secretory phase. MS = mid-secretory phase. LS = late-secretory phase. HGNC = Hugo Gene Nomenclature Committee. NCBI = National Centre for Biotechnology Information
